# Addictive behavior and incident gallstone disease: A dose–response meta-analysis and Mendelian randomization study

**DOI:** 10.3389/fnut.2022.940689

**Published:** 2022-10-10

**Authors:** Ye Bai, Min Zhang, Huijie Cui, Xuefeng Shan, Dongqing Gu, Yutong Wang, Mingshuang Tang, Xin Wang, Xia Jiang, Ben Zhang

**Affiliations:** ^1^Department of Epidemiology and Health Statistics, School of Public Health and Management, Chongqing Medical University, Chongqing, China; ^2^West China School of Public Health and West China Fourth Hospital, Sichuan University, Chengdu, China; ^3^Department of Pharmacy, The First Affiliated Hospital of Chongqing Medical University, Chongqing, China; ^4^Division of Non-Communicable Disease Epidemiology, The First Affiliated Hospital of Army Military Medical University, Chongqing, China; ^5^Department of Clinical Neuroscience, Center for Molecular Medicine, Karolinska Institute, Stockholm, Sweden

**Keywords:** addictive behavior, gallstone, cholecystectomy, meta-analyses, Mendelian randomization

## Abstract

**Background:**

Previous studies have suggested associations between addictive behavior and gallstone disease (GSD) risk, yet conflicting results exist. It also remains unclear whether this association is causal or due to confounding or reverse associations. The present study aims to systematically analyze the epidemiological evidence for these associations, as well as estimate the potential causal relationships using Mendelian randomization (MR).

**Methods:**

We analyzed four common addictive behaviors, including cigarette smoking, alcohol intake, coffee, and tea consumption (*N* = 126,906–4,584,729 participants) in this meta-analysis based on longitudinal studies. The two-sample MR was conducted using summary data from genome-wide associations with European ancestry (up to 1.2 million individuals).

**Results:**

An observational association of GSD risk was identified for smoking [RR: 1.17 (95% CI: 1.06–1.29)], drinking alcohol [0.84 (0.78–0.91)], consuming coffee [0.86 (0.79–0.93)], and tea [1.08 (1.04–1.12)]. Also, there was a linear relationship between smoking (pack-years), alcohol drinking (days per week), coffee consumption (cups per day), and GSD risk. Our MRs supported a causality of GSD incidence with lifetime smoking [1.008 (1.003–1.013), *P* = 0.001], current smoking [1.007 (1.002–1.011), *P* = 0.004], problematic alcohol use (PAU) [1.014 (1.001–1.026), *P* = 0.029], decaffeinated coffee intake (1.127 [1.043–1.217], *P* = 0.002), as well as caffeine-metabolism [0.997 (0.995–0.999), *P* = 0.013], and tea consumption [0.990 (0.982–0.997), *P* = 0.008], respectively.

**Conclusion:**

Our study suggests cigarette smoking, alcohol abuse, and decaffeinated coffee are causal risk factors for GSD, whereas tea consumption can decrease the risk of gallstones due to the effect of caffeine metabolism or polyphenol intake.

## Introduction

Addictive behavior causes a major public health concern, and it has a massive, long-term impact on human suffering and societal costs (1). Gallstone disease (GSD) is one of the most common problems in the digestive tract and a major public health issue worldwide. The incidence of GSD continues to rise (around 10–20% of all adults in Europe), and its etiology remains to be understood (2). The pathogenesis of GSD involves environmental triggers, genetic predispositions, and behavioral factors; and the major pathogenetic factors, including abnormal cholesterol metabolism and slow intestinal motility are related to metabolic syndrome (3). Addictive behavior is of increasing interest as it is one of the leading contributors to the global burden of GSD and can be modified to achieve a desired preventive effect (4). Therefore, it is imperative to understand the relationship between common addictive behaviors and incident GSD, including cigarette smoking, alcohol drinking, coffee intake, and tea consumption.

Epidemiological investigations have consistently shown that current smoking, alcohol drinking, and coffee consumption play a key role in the incidence of GSD (5–7). A previous meta-analysis of 10 studies (*N* = 4,213,482) has provided evidence that smokers have an estimated 11% increased risk of GSD per 10 cigarettes per day compared to non-smokers (8). Another meta-analysis conducted by Wang et al. (9) that involved 14 studies (*N* = 316,028) has identified a significant non-linear trend of GSD risk reduction associated with the increment of drinking alcohol (up to about 30 g per day). In addition, one meta-analysis based on six studies (*N* = 227,749) has observed a dose-dependent association of coffee consumption with GSD [0.95 (0.91–1.00), *P* = 0.049] (10). These meta-analyses, despite their large sample sizes, have several limitations. First, there lack analyses for different types of alcohol (liquor, beer, and wine) and coffee (caffeinated and decaffeinated). Also, not all addictive behaviors have been comprehensively examined (e.g., pack-year smoking and drinking days per week). Second, the majority of evidence is cross-sectional, and the observational nature of conventional epidemiological studies hinders causal inference hampered by confounding or reverse causality (11).

Mendelian randomization (MR) fills the gap of making causal inferences by using single nucleotide polymorphism (SNP) as an instrumental variable (IV) since SNPs are usually established before the development of disease and therefore independent of confounders (12). Indeed, Yuan et al. have found that smoking is causally associated with GSD risk (13). However, tobacco smoking is a highly addictive behavior that contains large amounts of substances, such as nicotine, cannabis, and exposure to tobacco smoke (ETS) the causality of them with GSD has not yet been investigated. As for drinking alcohol, although common alcohol use was not significantly causally associated with the GSD risk, we additionally analyzed the causality between problematic alcohol use (PAU) and the risk of GSD. Moreover, a recent genome-wide association study (GWAS) of caffeine intake has identified additional SNPs associated with coffee or tea consumption, which can be used as IVs for further MR (14). Note that the effect of consuming tea on the GSD risk lacks systematic evaluation.

The current study aims to comprehensively evaluate the relationship between these common addictive behaviors and the GSD risk. We first summarized the evidence in one updated meta-analysis only including a longitudinal study. Data from the meta-analysis was further tested for potential dose–response relationships and by trial sequential analysis (TSA) to check if the present evidence is conclusive. We then explored a putative causal association of tobacco smoking, alcohol use, caffeine intake, and tea consumption with the risk of GSD using a two-sample MR design.

## Materials and methods

### Search strategy and meta-analysis

Our meta-analysis has been registered at PROSPERO (CRD42020179076) and following PRISMA checklists. We searched PubMed and Embase databases for studies published before January 2021, and references to the retrieved articles were manually searched for additional information (Supplementary Table 1). The flow chart is presented in Supplementary Figure 1. GSD was defined as gallstones diagnosed by ultrasonography or a history of cholecystectomy; participants without gallstones or cholecystectomy were considered as the control group (15). Longitudinal studies, including nested case-control, cohort, and randomized controlled trials, provided sufficient data for calculating the effect sizes with 95% confidence intervals (CI) and were eligible for our analysis (see Supplementary Table 2). If the person-years of subgroup GSD cases were not reported, we calculated the proportion of new total cases for each group (dividing the exact number of GSD by RR) and multiplied the proportion by total person-years as described previously (16). Two authors (Y.B. and X.W.) extracted data back-to-back from identified articles in current research, and disagreement was solved by consensus.

DerSimonian and Laird’s random-effect meta-analysis was applied to summarize the association between addictive behaviors and GSD when *I*^1^ exceeded 50%; otherwise, a fixed-effect meta-analysis was conducted (17, 18). Heterogeneity sources were explored by conducting subgroup analyses. Funnel plots were drawn to demonstrate the possible publication bias if asymmetry were observed, and the bias would be further tested after combining with Egger’s and Begg’s test results (19). The pooled effect was adjusted by Duval and Tweedie’s trim-and-fill method to account for publication bias (20). Sensitivity was evaluated by omitting each estimate at one time to see to what extent a single study could influence the overall risk estimate. Pooled analyses were done using Comprehensive Meta-Analysis version 3.0 (Biostat, Englewood, NJ, USA).

### Dose–response analysis

To investigate whether the dose of addictive substances intake was associated with GSD, we conducted Greenland and Longnecker’s method using linear and non-linear models (21). The mean amount was used to assign the exposure levels for each risk estimate. For the open-ended lower boundary, the level was assumed to be zero, and non-taken was considered as the reference category. For the open-ended upper boundary, the highest level was assigned to 1.5 or 1.2 times the lower boundary of the category (22). In this study, we further tested a dose-dependent association of GSD with smoking status (cigarettes per day and pack-year smoking), consuming alcohol (drinking grams per day, alcohol intake times per week, and drinking days per week), and intaking caffeine (coffee or tea consumption-cups per day). These statistical analyses were done with the use of STATA 16.0 (StataCorp. College Station, TX, USA).

### Trial sequential analysis

TSA was applied to evaluate the sufficiency of the total sample size of a meta-analysis to investigate the associations. A cumulative *Z*-curve exceeds the trial sequential monitoring limit or the required information size, suggesting conclusive evidence (23). TSA was conducted by the program version 0.9 beta.^2^ All statistical significance were determined by *P* < 0.05.

### Genetic instruments selection and outcome data sources

SNPs showing genome-wide significance (*P* < 5.0 × 10^–8^) and with *R*^2^ < 0.1 identified by *LDlink*^2^ were used as IVs for lifetime smoking (i.e., ever and never smokers, smoking duration, heaviness, and cessation in ever smokers were taken into account) (24). The selection of IVs for smoking initiation (including ever-smoking, current-smoking, and smoking cessation) and common alcohol drinking, for (PAU, considering both alcohol use disorder and measures of problematic drinking), and for caffeine intake (the caffeine content per cup was multiplied by the number of cups of tea or coffee) were retrieved from three GWASs, respectively (14, 25, 26). All study populations were European descendants. The strength of instruments used in this study has been previously described, and an F-statistic larger than 10 was regarded as a strong instrument (27). Details are available in Supplementary Tables 3, 4.

GSD cases and controls were obtained from the UK Biobank, a cohort of about 500,000 adults recruited during 2006–2010 in the United Kingdom (28). Three sources of case-control GWAS were used. First, data containing 337,199 individuals (6,986 cases and 330213 controls, all patients with definite diagnoses, i.e., ICD10: K80_cholelithiasis) with GWAS performed by the Neale Lab (id: ukb-a-559). Second, self-reported gallstones (*N*_*g*_ = 462,933, 7682/455251) obtained from UKB, MRC-IEU (id: ukb-b-18700). Third, the symptomatic GSD with a history of cholecystectomy from the UKB (id: ukb-b-6235, N_*c*_ = 462,933, 18319/444614).

### Mendelian randomization analysis

For our MR study, the multiplicative random-effect inverse variance weighted (IVW) method was used to estimate the causal associations between addictive behaviors and GSD risk. In sensitivity analysis, the MR-Egger regression was used to identify and correct for the horizontal pleiotropy, the weighted median method provides the estimates when SNPs accounting for more than half of the weight are valid, and the maximum likelihood method maximizes the likelihood of the model based on the causal association (29–31). The *p-*value of the MR-Egger intercept was used to indicate potential horizontal pleiotropy, and Cochrane’s *Q*-value was used to evaluate the heterogeneity among those SNPs for each addictive behavior (32). In this study, the large sample size allowed us to gain sufficient power (all were greater than 80%) for conclusive estimation of the associations between addictive behaviors and incident GSD. The analyses were performed *via* the MR-Base^3^ using the R package “TwoSampleMR” (version 4.0.3, R Foundation for Statistical Computing, Vienna, Austria). Here, the causal association would be considered statistically significant when a Bonferroni corrected *P-*value was less than 0.013 (correcting for four exposures, including tobacco smoking, alcohol drinking, coffee, and tea consumption). A *p*-value < 0.05 was regarded as the marginal significance.

## Results

### Meta-analysis

In our meta-analysis, a total of 27 longitudinal studies with 43 datasets were included in the pooled analysis, incorporating cigarette smoking (*N* = 4,584,729), alcohol intake (*N* = 1,819,052), coffee consumption (*N* = 333,773), and tea consumption (*N* = 126,906). A positive significant association of incident GSD was observed with smoking [RR: 1.17 (95% CI: 1.06–1.29)] and tea consumption [1.08 (1.04–1.12)]; while a negative significant association of intaking alcohol [0.84 (0.78–0.91)] or coffee [0.86 (0.79–0.93)] with GSD was found (Table 1 and Supplementary Figure 2). The associations were directionally consistent when stratified by sex, ethnicity, underwent cholecystectomy, or according to different types of addictive behaviors (Supplementary Figures 3–7). Of note, a significant increment in GSD risk was associated with smoking only in males (1.15 [1.11–1.20]), and current smokers increased about 7% risk of GSD compared to former smokers. Consuming coffee was significantly associated with a decrement of GSD risk [0.87 (0.79–0.96)] in females only. Although an association with GSD [0.84 (0.82–0.87)] was found in caffeinated coffee, it was not statistically significant in decaffeinated coffee (Table 1). We also assessed the potential publication bias, and the adjusted funnel plot is shown in Supplementary Figure 8. Then a sensitivity analysis suggested that one of each included study did not influence the overall estimate of the meta-analysis (Supplementary Figure 9). Moreover, there were significant differences across all dose levels of cigarette smoking and alcohol intake with the risk of GSD.

**TABLE 1:** Addictive behaviors risks for gallstone disease included in meta-analysis.

	No. of studies	Sample size	*I*^2^ (%)	Pooled RR (95% *CI*)	*P*_*between*_
**Cigarette smoking**	**17**	**4,584,729**	**96.98**	**1.17 (1.06–1.29)**	
Female	8	4,377,535	98.65	1.16 (0.99–1.35)	0.95
Male	4	94,985	0.00	1.15 (1.11–1.20)	
America	8	3,136,107	97.82	1.18 (1.01–1.37)	0.82
Europe	7	1,412,481	71.91	1.14 (1.06–1.22)	
Asia	2	36,141	0.00	1.19 (1.03–1.38)	
With cholecystectomy	3	2,858,469	99.52	1.28 (0.87–1.88)	0.55
Without cholecystectomy	2	102,284	0.00	1.13 (1.02–1.25)	
Smoking status	9	1,753,267			0.02
Former smoker			0.00	1.11 (1.09–1.14)	
Current smoker			46.68	1.18 (1.13–1.24)	
Smoking pack-years	2	152,240			0.01
≤ 20			0.00	1.15 (1.08–1.23)	
> 20			0.00	1.31 (1.21–1.40)	
**Alcohol intake**	**17**	**1,819,052**	**97.94**	**0.84 (0.78–0.91)**	
Female	8	1,566,845	94.29	0.85 (0.79–0.91)	0.38
Male	8	142,364	82.55	0.89 (0.82–0.97)	
America	7	391,307	69.50	0.85 (0.81–0.89)	0.21
Europe	8	1,391,604	99.22	0.79 (0.68–0.91)	
Asia	2	36,141	24.35	0.97 (0.81–1.17)	
With cholecystectomy	1	139,272	0.00	0.87 (0.84–0.89)	0.88
Without cholecystectomy	2	92,880	95.62	0.84 (0.58–1.22)	
Type of drinks	3	106,342			0.60
Beer			20.42	0.84 (0.73–0.96)	
Wine			0.00	0.87 (0.81–0.93)	
Liquor			0.00	0.82 (0.75–0.90)	
Frequency of intake (days/week)	2	104,380			0.02
1–2			0.00	0.94 (0.88–1.02)	
3–4			0.00	0.85 (0.77–0.95)	
5–7			62.49	0.77 (0.66–0.88)	
Grams intake per day	2	104,380			0.00
< 15			0.00	0.91 (0.85–0.96)	
≥ 15			1.47	0.71 (0.64–0.78)	
**Coffee Consumption**	**7**	**333,773**	**86.88**	**0.86 (0.79**–**0.93)**	
Female	4	127,384	78.11	0.87 (0.79–0.96)	0.98
Male	4	99,452	77.25	0.87 (0.74–1.02)	
America	2	126,906	73.51	0.83 (0.68–1.00)	0.80
Europe	5	206,867	85.43	0.85 (0.75–0.96)	
Type of Coffee	2	126,906			0.00
Caffeinated coffee Caffeinated coffee intake			29.61	0.84 (0.82–0.87)	
Decaffeinated coffee intake			0.00	1.00 (0.96–1.05)	
Cups of Coffee per day	3	231,399			0.17
≤ 1			0.00	0.92 (0.87–0.97)	
2–3			65.03	0.81 (0.77–0.86)	
3–6			0.00	0.83 (0.71–0.97)	
≥ 6			0.00	0.77 (0.61–0.97)	
**Tea consumption**	**2**	**126,906**	**3.30**	**1.08 (1.04–1.12)**	
Female	1	80,898	0.00	1.07 (1.03–1.12)	0.71
Male	1	46,008	52.90	1.10 (0.98–1.23)	
America	2	126,906	0.00	1.07 (1.04–1.11)	**—**
Cups of tea per day	2	126,906			0.41
≤ 1			0.00	1.08 (1.03–1.13)	
2–3			44.76	1.05 (0.97–1.12)	
≥ 4			0.00	1.15 (1.02–1.29)	

The bold values here are meant to indicate the overall estimate of total studies.

**FIGURE 1:**
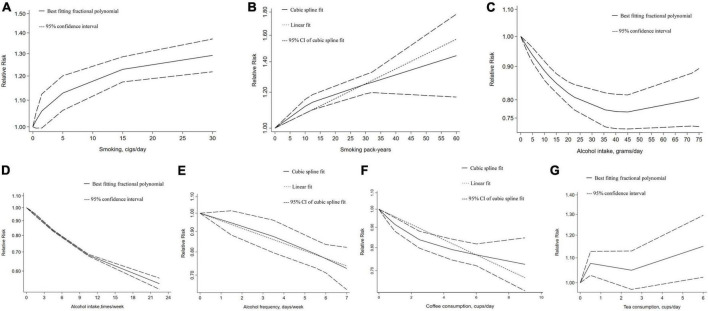
Dose–response relationship between addictive substance intake and the gallstone risk. **(A)** Smoking cigarettes per day, **(B)** smoking pack-years, **(C)** alcohol intake grams per day, **(D)** alcohol intake times per week, **(E)** alcohol drinking days per week, **(F)** coffee consumption cups per day, **(G)** tea consumption cups per day. Two-term best fitting fractional polynomial regression model indicated that a potential non-linear model fitting the observed outcomes is identified. A single cubic spline curve is fitted to the data and the goodness of non-linear fit is calculated. Since the non-linear fit was not significant and it was similar to the linear fit model, so linear regression is used.

Here, our dose–response meta-analysis showed a non-linear relationship between GSD risk with daily smoking per 10 cigarettes [1.10 (1.08–1.12), *P*_nonlinearity_ ≤ 0.01]. We further detected a linear association that an increment of pack-years of smoking increased the risk of GSD [1.01 (1.01–1.01), *P* = 0.08] (Figure 1). However, there was no significant association between alcohol consumed grams per day and GSD, despite a non-linear relationship being found. A significant non-linear association of alcohol intake times per week was observed for GSD risk reduction with an RR of [0.81 (0.67–0.99), *P* ≤ 0.01)] per 5 units. We further found an increment of days per week of alcohol drinking decreased the GSD risk with a linear inverse association [0.96 (0.94–0.97), *P* = 0.89]. As for caffeine consumption, a potential linear association was detected between coffee cups per day and GSD risk [0.95 (0.94–0.96), per 1 cup]. Despite a non-linear relationship between GSD risk with consuming tea-cups per day (*P* = 0.01), we found no significant association. In addition, the risk of GSD increased by 4% and 8% with every 5 and 10 pack-years increments in cigarettes-smoking; while the risk was reduced by 20% and 23% per five units increment in alcohol-drinking days per week and coffee-cups per day (Supplementary Table 5).

### Trial sequential analysis

In the TSA of our meta-analysis, the cumulative *Z*-curve crossed trial sequential monitoring and/or conventional boundary and penalized tests adjusted *Z*-curves also presented similar results, denoting that this evidence was robust and conclusive (Supplementary Figure 10). Compared with the control, the adjusted RR of GSD was 1.13 (1.06–1.20) in smoking, 0.73 (0.67–0.80) in alcohol, or 0.82 (0.75–0.90) in coffee. For the subgroup analyses, the adjusted RR of GSD was 1.10 (1.07–1.14) in former-smoking and 1.16 (1.12–1.20) in current-smoking. For the different types of alcohol, the adjusted RR of GSD was 0.70 (0.62–0.78) for beer, 0.72 (0.66–0.77) for wine, and 0.78 (0.70–0.87) for liquor, respectively.

### Mendelian randomization analyses

**FIGURE 2:**
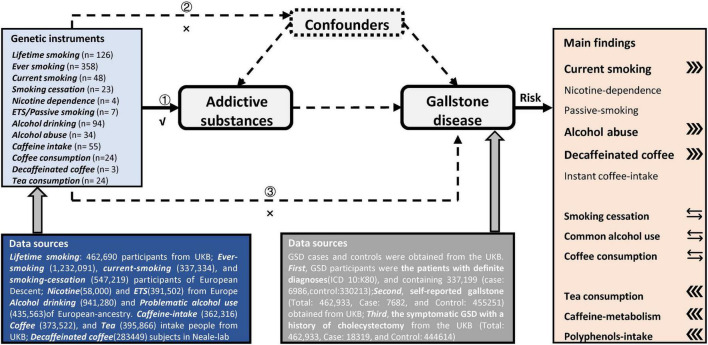
Overview of the design and main findings in this Mendelian-randomization study. Assumption 1 indicates that the genetic instruments are significantly genome-wide associated with these addictive substances of interest. Assumption 2 indicates that our genetic instruments should not be associated with confounders. Assumption 3 indicates that genetic instrument affect these outcomes only *via* the exposures.

As shown in Figure 2 and Supplementary Figure 11, our MR found that genetically predicted current smoking and PAU both were associated with an increased risk of GSD, while genetically predicted tea consumption was associated with a decreased risk of GSD. However, there was no genetic association between smoking cessation, common alcohol use, coffee consumption, and the risk of GSD.

#### The causal association between cigarette smoking and incident gallstone disease

In the IVW method, using lifetime-smoking associated 120 independent SNPs as IVs, we found that it had a causal effect on diagnosed cholelithiasis (OR: 1.008, 95% CI: 1.003–1.013, *P* = 0.001) and patients underwent cholecystectomy [1.015 (1.008–1.023), *P* = 6.9 × 10^–5^], but not for self-reported gallstones [1.005 (1.001–1.009), *P* = 0.024] when compared with *P*_adjusted_ < 0.013. The estimates remained directional and consistent observed in MR-Egger regression, despite the causalities were not significant. Then, by using the intercept of MR-Egger, we observed no evidence of horizontal pleiotropy (*P*_pleiotropy_ for the diagnosed cholelithiasis, self-reported gallstones, and cholecystectomy: 0.399, 0.658, and 0.693), and the weighted median and maximum likelihood methods yielded similar results, which illustrated the high stability of this causality.

This positive association of all outcomes was further confirmed by smoking initiation associated SNPs (*N*_IV_ = 345, 338, and 342). As for ever-smoking, it was associated with an increased risk of diagnosed cholelithiasis [1.006 (1.003–1.008), *P* = 2.2 × 10^–6^], self-reported gallstones [1.003 (1.001–1.005), *P* = 0.001], and cholecystectomy [1.009 (1.005–1.012), *P* = 4.1 × 10^–8^] in IVW. There was no detected horizontal pleiotropy by using MR-Egger (all *P*_pleiotropy_ ≥ 0.05), and similar effects were observed using the weighted median or maximum likelihood method. For the subgroup analyses, current-smoking also significantly increased the risk of diagnosed cholelithiasis [1.007 (1.002–1.011), *P* = 0.004], whereas it disappeared in smoking-cessation [1.000 (0.994–1.007), *P* = 0.905]. This study, for the first time, provided an explanation of the pathogeny of GSD with addictive behavior, the results of our MR showed that nicotine dependence was a major risk factor for GSD [1.012 (1.002–1.022), *P* = 0.017], and ETS was also causally associated with the risk of cholecystectomy [1.043 (1.002–1.086), *P* = 0.038] (see online Supplementary Table 6).

#### A potential relationship of the risk of gallstones with alcohol drinking

Regarding common alcohol use (*N*_IV_ = 88, 84, and 89), no causal association of GSD risk was found, and this result might be due to the presence of heterogeneity (all *P*_*Q*_ ≤ 0.001). In this MR, we further found that PAU was potentially associated with the risk of diagnosed cholelithiasis (*N*_IV_ = 28, 1.014 [1.001–1.026], *P* = 0.029) and self-reported gallstones (*N*_IV_ = 28, 1.012 [1.001–1.023], *P* = 0.028), but not cholecystectomy [*N*_*IV*_ = 29, 1.019 (0.996–1.042), *P* = 0.104] in IVW. Moreover, the effects attenuated slightly in MR-Egger regression with the intercept (0.613, 0.204, and 0.170) confirming that pleiotropy was not detected in the three outcomes. Similarly, the positive estimate was identified for the risk of GSD with PAU in the maximum likelihood method.

#### A highly debated association between coffee consumption and the gallstone risk

No significant causal association of GSD risk with coffee consumption was found in this IVW. Even though removing an SNP (rs2472297), the null effect on GSD with consuming coffee was not altered [0.996 (0.987–1.005) for cholelithiasis, 0.999 (0.989–1.008) for gallstones, and 0.998 (0.980–1.017) for cholecystectomy]. While genetically predicted caffeine-intake significantly decreased the GSD risk in the weighted median [0.994 (0.989–0.999), *P* = 0.012] for self-reported gallstones; [0.991 (0.984–0.998), *P* = 0.016] for cholecystectomy. Although all of the *P*_Q_ ≤ 0.001 and heterogeneity existed, no horizontal pleiotropy was detected by MR-Egger with the intercept of 0.136, 0.164, and 0.113 in our outcomes. Furthermore, we analyzed the associations between the metabolism of caffeine and the incidence of GSD (Supplementary Table 7). There was a negative causal association between habitual caffeine-intake [0.993 (0.988–0.997), *P* = 0.003], caffeine-metabolism [0.997 (0.995–0.999), *P* = 0.013], and the GSD risk; whereas decaffeinated coffee [1.127 (1.043–1.217), *P* = 0.002] or instant coffee [1.074 (1.016–1.135), *P* = 0.012] was related with the GSD risk increased.

#### The directly protective effect on the risk of gallstone disease with tea consumption

Consuming tea (an additional source of caffeine mainly in black tea) was significantly negatively associated with GSD risk in IVW (Table 2). For diagnosed cholelithiasis [*N*_*IV*_ = 19, 0.990 (0.982–0.997), *P* = 0.008], self-reported gallstones [*N*_*IV*_ = 18, 0.993 (0.987–0.999), *P* = 0.014] and cholecystectomy [*N*_*IV*_ = 18, 0.983 (0.974–0.992), *P* = 0.000]. Although an association was not significant, the estimate remained directional and consistent as observed in MR-Egger; and similar estimates were also obtained in the weighted median and maximum likelihood methods. For other sensitivity analyses, no heterogeneity existed with the funnel plot presented symmetrically in Supplementary Figure 12.

**TABLE 2:** Mendelian randomization estimate of a causal association between addictive behaviors and the risk of gallstones.

Outcome disease	Exposure phenotype	Number of IVs	Inverse variance weighted	MR egger	Weighted median	Maximum likelihood	Horizontal pleiotropy	Q_pval
			OR (95% CI), *P*-value	OR (95% CI), *P*-value	OR (95% CI), *P*-value	OR (95% CI), *P*-value		
Diagnoses ICD10 K80: Cholelithiasis (Neale Lab)	Lifetime smoking ^ac^	120	1.008 (1.003–1.013), 0.001	1.000 (0.982–1.020), 0.965	1.008 (1.001–1.015), 0.030	1.008 (1.004–1.013), 0.000	0.399	0.244
	Ever smoking ^acd^	345	1.006 (1.003–1.008), 2.2E–06	1.000 (0.991–1.010), 0.954	1.004 (1.001–1.008), 0.007	1.006 (1.003–1.008), 4.2E–07	0.263	0.025
	Current smoking ^ac^	47	1.007 (1.002–1.011), 0.004	1.004 (0.996–1.011), 0.353	1.006 (0.999–1.012), 0.096	1.007 (1.002–1.011), 0.004	0.382	0.719
	Smoking cessation	21	1.000 (0.994–1.007), 0.905	0.993 (0.977–1.010), 0.441	0.998 (0.991–1.005), 0.598	1.000 (0.995–1.005), 0.877	0.375	0.025
	Common alcohol use	88	0.997 (0.989–1.006), 0.531	0.992 (0.976–1.008), 0.344	0.999 (0.987–1.011), 0.885	0.997 (0.991–1.004), 0.420	0.466	0.000
	Problematic alcohol use ^bc^	28	1.014 (1.001–1.026), 0.029	1.002 (0.959–1.048), 0.918	1.009 (0.998–1.020), 0.115	1.014 (1.007–1.022), 0.000	0.613	5.0E–07
	Caffeine intake	42	0.998 (0.992–1.004), 0.530	0.992 (0.983–1.002), 0.119	0.995 (0.989–1.002), 0.154	0.998 (0.994–1.002), 0.369	0.136	0.000
	Coffee consumption	18	0.996 (0.987–1.005), 0.418	0.989 (0.971–1.006), 0.228	0.993 (0.982–1.004), 0.199	0.996 (0.989–1.003), 0.272	0.344	0.017
	Tea consumption^ac^	19	0.990 (0.982–0.997), 0.008	0.986 (0.969–1.002), 0.113	0.992 (0.982–1.003), 0.159	0.990 (0.982–0.997), 0.009	0.601	0.733
Self–reported: Gallstones (MRC-IEU)	Lifetime smoking ^bc^	117	1.005 (1.001–1.009), 0.024	1.009 (0.991–1.027), 0.327	1.004 (0.998–1.009), 0.175	1.005 (1.001–1.009), 0.007	0.658	0.002
	Ever smoking ^ac^	338	1.003 (1.001–1.005), 0.001	0.998 (0.991–1.006), 0.675	1.003 (1.001–1.006), 0.017	1.003 (1.002–1.005), 0.000	0.218	0.023
	Current smoking	46	1.001 (0.997–1.005), 0.554	0.999 (0.992–1.007), 0.820	1.000 (0.995–1.006), 0.973	1.001 (0.998–1.005), 0.473	0.508	0.022
	Smoking cessation	20	1.002 (0.997–1.006), 0.477	0.990 (0.979–1.001), 0.101	1.002 (0.996–1.007), 0.597	1.002 (0.998–1.006), 0.417	0.044	0.157
	Common alcohol use	84	0.997 (0.990–1.005), 0.463	1.013 (0.992–1.035), 0.236	1.004 (0.995–1.014), 0.357	0.997 (0.992–1.003), 0.363	0.130	0.001
	Problematic alcohol use ^bc^	28	1.012 (1.001–1.023), 0.028	0.988 (0.951–1.026), 0.533	1.003 (0.994–1.012), 0.537	1.012 (1.007–1.018), 2.4E–05	0.204	0.000
	Caffeine intake ^d^	41	1.000 (0.994–1.005), 0.899	0.994 (0.985–1.004), 0.237	0.994 (0.989–0.999), 0.012	1.000 (0.996–1.003), 0.822	0.164	0.000
	Coffee consumption	17	0.999 (0.989–1.008), 0.809	0.993 (0.974–1.012), 0.477	0.994 (0.986–1.002), 0.131	0.999 (0.993–1.004), 0.665	0.490	1.3E–05
	Tea consumption ^b^	18	0.993 (0.987–0.999), 0.014	0.989 (0.976–1.002), 0.124	0.991 (0.982–0.999), 0.024	0.993 (0.987–0.999), 0.015	0.558	0.614
Operation code: Cholecystectomy (UKBiobank)	Lifetime smoking ^acd^	120	1.015 (1.008–1.023), 6.9E–05	1.021 (0.992–1.051), 0.165	1.014 (1.006–1.023), 0.001	1.016 (1.010–1.021), 3.6E–08	0.693	1.4E-08
	Ever smoking ^acd^	342	1.009 (1.005–1.012), 4.1E-08	1.012 (0.999–1.025), 0.081	1.009 (1.005–1.013), 3.9E-06	1.009 (1.006–1.011), 5.1E-11	0.628	4.8E-08
	Current smoking ^c^	48	1.007 (1.000–1.014), 0.068	0.998 (0.986–1.010), 0.749	1.004 (0.996–1.012), 0.282	1.007 (1.002–1.012), 0.011	0.098	0.000
	Smoking cessation	23	1.004 (0.998–1.010), 0.221	0.991 (0.975–1.006), 0.240	1.003 (0.995–1.011), 0.463	1.004 (0.998–1.010), 0.172	0.077	0.187
	Common alcohol use	89	0.994 (0.979–1.009), 0.410	0.991 (0.963–1.019), 0.535	1.001 (0.986–1.016), 0.943	0.993 (0.986–1.001), 0.088	0.832	1.0E–36
	Problematic alcohol use ^c^	29	1.019 (0.996–1.042), 0.104	0.980 (0.925–1.039), 0.506	1.007 (0.993–1.020), 0.331	1.020 (1.011–1.029), 5.2E–06	0.170	0.000
	Caffeine intake	41	0.999 (0.988–1.010), 0.859	0.987 (0.970–1.005), 0.172	0.991 (0.984–0.998), 0.016	0.999 (0.994–1.004), 0.692	0.113	0.000
	Coffee consumption	17	0.998 (0.980–1.017), 0.854	0.979 (0.945–1.015), 0.269	0.988 (0.977–0.999), 0.027	0.998 (0.990–1.006), 0.664	0.240	2.6E–11
	Tea consumption ^acd^	18	0.983 (0.974–0.992), 0.000	0.977 (0.956–0.999), 0.054	0.979 (0.967–0.991), 0.000	0.983 (0.974–0.992), 0.000	0.559	0.300

Significant associations reported are adjusted p-values at a Bonferroni corrected and p-values directly, ^a^IVW method: P_adjusted_ < 0.013, ^b^IVW method: P_val_ < 0.050, ^c^Maximum Likelihood: P_adjusted_ < 0.013, ^d^Weighted Median: P_adjusted_ < 0.013, If significant heterogeneity existed P_Q_ < 0.05, a random-effects model (IVW) was selected.

## Discussion

As summarized in this study, the meta-analysis based on a longitudinal study indicates that addictive behaviors are significantly associated with the incidence of GSD. Compared with never smokers, current smokers have a positive dose-dependent response to GSD risk, and the evidence is further verified by the MR analysis. There are negative dose–response relationships between common alcohol use, coffee intake, and the GSD risk. However, the results of MR do not confirm the causal relationship between them. The novel finding of this study is that alcohol abuse may be causally associated with an increment in GSD risk, whereas tea consumption has a protective effect on the GSD risk in Europe.

Smoking has been shown to alter lipid metabolism, and the abnormal synthesis of bile may cause cholesterol supersaturation for the formation of gallstones (33). Consistent with a previous dose-response meta-analysis (8), the risk of GSD is found to be increased by smoking (cigarettes per day) with a non-linear relationship in our study. Moreover, a finding suggests that it is a linear dose-response association between pack years of smoking and risk of GSD and is further subjected to causality. It is also notable that the causal association of GSD is not significant in smoking cessation.

To date, evidence linking alcohol drinking with GSD is controversial (9). In this meta-analysis, we verify a negatively non-linear dose–response association between drinking alcohol grams per day and GSD risk, consistent with Cha et al. (34), but the strict study design and dose definition are used in our study. We conclude that the association of GSD risk reduction appears to reach the limit when the dose is higher than 45 g/day, and this finding (J-shaped) is similar to that of Figueiredo et al. (5), while the appropriate dose of alcohol-intake protects against GSD awaits future study. Concerning the types of alcohol, an RR of GSD is 0.82, 0.84, and 0.87 (liquor, beer, and wine) as the alcohol concentration decreased. One possible explanation is that alcohol may reduce cholesterol levels, improve HDL-C levels, and promote the secretion of bile acid, which in turn may inhibit gallstone formation (35). Meanwhile, we also propose two possible explanations for a non-causality found between common alcohol use and GSD risk in this MR. One possibility is that there may be a mediation effect for liver cirrhosis in the relationship. Some studies indicate that alcohol drinking increases the risk of liver cirrhosis, which has a close correlation with incident GSD (36, 37). Second, it may have a potential non-linear relationship between them that moderate drinking decreases the risk of GSD, whereas problematic drinking increases the GSD risk.

Interestingly, high coffee consumption was associated with a decrement in GSD risk. An MR also suggested a causal relationship between them in the Danish cohort (38). But, our results of MRs do not support such putative causality from a larger sample size in UKB, which agrees with a finding reported by Yuan et al. (13). In addition to population differences, one intriguing possibility is that self-reported coffee consumption includes decaffeinated coffee, coffee beverages, and others, which may weaken the effect of caffeine (39). Furthermore, this study provides the first report, to our knowledge, of a negative correlation of the GSD risk with drinking tea in Europe.

Tea consumption as another addictive behavior with caffeine intake (including black and green tea) has been associated with a GSD risk decreased in both genders within the population of Asia, and caffeine can stimulate cholecystokinin secretion and release bile acids into the intestine (40, 41). Our MR verified this causal association in a European population, and tea polyphenol (mainly found in green tea) was also found to be causally associated with GSD risk, despite only one instrument being used. Certainly, more studies need to be done in the future. Current knowledge shows that polyphenols may accelerate bowel movements, and promote lipolysis and absorption, which in turn decreases morbidity in GSD (42).

Here, some plausible mechanisms are explored for the causal associations between addictive substance use and GSD risk. For the association between active smoking or ETS and the risk of GSD, the nicotine-dependence may be a key factor in this relationship. Of note, electronic cigarette has not been reported in GSD-related research. In addition, caffeine and tea polyphenols are the most commonly consumed psychostimulants, and they both causally decrease the risk of GSD in our MR. However, coffee consumption (including decaffeinated coffee or other beverage) is not associated with GSD, which may weaken the effect of caffeine.

There is high heterogeneity in our meta-analysis, and existing research regarding this topic is relatively fewer, which may have yielded publication bias. For example, the positive association between tea consumption and GSD in the American population might be due to the smaller number of studies included, which is an important limitation of our study. The second limitation is vertical pleiotropy, which could be shown to mediate the effect within a relationship between exposure and outcome. Another limitation is that we could not explore a non-linear relationship using this MR approach. As for the heterogeneity in a different population, our IVs were all identified in GWAS of a European-origin sample, although these instruments can only explain the percent of 0.24–1.72 (smoking cessation, caffeine-intake, etc.) in total estimated heritability, which limits the generalizability of our finding to diverse populations.

## Conclusion

In conclusion, tobacco smoking, PAU, and decaffeinated coffee directly confer high risks of GSD; nonetheless, habitual caffeine intake and tea consumption may have a protective role against GSD due to an effect of caffeine metabolism or polyphenol intake. Accordingly, we infer that changing addictive behavior may be necessary for reducing the risk of GSD.

## Data availability statement

The original contributions presented in this study are included in the article/Supplementary material, further inquiries can be directed to the corresponding authors.

## Author contributions

BZ managed the project and study design. XW and YB read and abstracted the studies included in the meta-analysis. XJ and YB analyzed the data in the Mendelian randomization study. MZ and YB prepared the tables and figures. DG, MT, and YB did the statistical analyses. YB drafted the manuscript with HC, XS, YW, XW, and XJ. All authors reviewed and approved the article.

## Footnotes

1
http://www.ctu.dk/tsa

2
https://analysistools.cancer.gov

3
http://www.mrbase.org/
