# Supplementary material for: Addictive behavior and incident gallstone disease: A dose–response meta-analysis and Mendelian randomization study
Source: Front Nutr. 2022 Oct 10;9:940689. doi: 10.3389/fnut.2022.940689 (PMC9589252; doi:10.3389/fnut.2022.940689)
Supplement: Supplementary file 1 [file Data_Sheet_1.PDF]

Appendix

Table S1 – Literature search terms and syntax…………………………………………………………………....1

Table S2 – Longitudinal studies of addictive behaviors associated with GSD risk included in meta-analysis……...........2

Table S3 – Characteristic information for instrumental variables of the addictive behaviors…………………………......7

Table S4 – Characteristics of genetic instruments associated with the addictive behaviors………………………………8

Table S5 – Dose-response relationship between addictive behaviors and GSD risk with linear or spline model...............39

Table S6 – Mendelian randomisation analysis for the causal association between smoking substance-use and GSD.......40

Table S7 – Mendelian randomisation analysis for the causal association between caffeine consumption and GSD…….40

Figure S1–Flowchart of study selection for inclusion in meta-analysis……………………………………………....42

Figure S2–Addictive behaviors overall pooled effect for GSD risk…………………………………………………..43

Figure S3–Forest plots of studies on addictive behaviors and the risk of GSD with subgroup-analysis by gender............44

Figure S4–Forest plots of studies on addictive behaviors and the GSD risk with subgroup-analysis by geographic…….45

Figure S5–Forest plots of subgroup-analysis for studies on smoking status and GSD risk……………………………...46

Figure S6–Forest plots of subgroup-analysis for studies on alcohol intake and GSD risk………………………………47

Figure S7–Forest plots of subgroup-analysis for studies on coffee or tea consumption and GSD risk…………………..48

Figure S8–Funnel plots of studies on addictive behaviors and the risk of GSD…………………………………………49

Figure S9–Forest plots of studies with removed each one and cumulative effects on addictive behaviors and GSD risk..50

Figure S10–Trial sequential analysis in meta-analysis of the addictive behaviors and the risk of GSD………………..52

Figure S11–Scatter plots of MR-analysis in addictive behaviors and the risk of GSD……………………………......55

Figure S12–Funnel plots of MR-analysis in addictive behaviors and the risk of GSD………………………………...60

PRISMA_2020_checklist………………………………………………………………………………….65

Table 1. Literature search terms and syntax

(((“gallstone” OR “gallstones” OR “gallstone disease” OR “cholelithiasis” OR “gallbladder stones” OR “gallbladder calculus” OR “biliary tract stones” OR “bile duct stones” OR “choledocholithiasis”) AND (“odds ratio” OR “OR” OR “hazard risk” OR “hazard ratio” OR “HR” OR “relative risk” OR “RR” OR “rate ratio” OR “P” OR “P value” OR “P=” OR “association” OR “associated” OR “confidence interval” OR “CI” OR “censor” OR “Kaplan-Meier” OR “Cox model” OR “Proportional hazard model” OR “log-rank” OR “survival analysis”)) AND (“risk” OR “cohort” OR “nested case-control” OR “trial” OR “prospective” OR “follow-up” OR “registry” OR “record linkage” OR “longitudinal” OR “incidence”))

Table 2.1. Longitudinal studies of smoking status associated with gallstones risk

study ID	PMID	Author	Year	Subjects source	Country	Gender	Period	Expusure group	Control group	Disease	Diagnosis	Follow-up	Person years	Total (n_i)	Mean age	Effect	Effect size	LCI	UCI
Stampfer-1992-1	1550039	Stampfer	1992	the Nurses' Health Study cohort II	American	Female	1980-1988	Former smoker	Never smoking	Gallstones disease	follow-up questionnaires,self-reported or ultrasound records	6.7	607104	90302	34-59 y	RR	**1.06**	**0.96**	**1.17**
Stampfer-1992-2								Current smoker									**1.17**	**1.07**	**1.27**
Stampfer-1992-2-1								1-14 c/d									1.10	0.93	1.29
Stampfer-1992-2-2								15-24 c/d									1.03	0.90	1.19
Stampfer-1992-2-3								25-34 c/d									1.31	1.09	1.58
Stampfer-1992-2-4								35+ c/d									1.51	1.20	1.89
Stampfer-1992-3								Former smoker		Gallstones							**1.09**	**0.89**	**1.33**
Stampfer-1992-4								Current smoker									**1.08**	**0.89**	**1.30**
Stampfer-1992-4-1								1-14 c/d									1.06	0.74	1.50
Stampfer-1992-4-2								15-24 c/d									0.93	0.68	1.29
Stampfer-1992-4-3								25-34 c/d									1.21	0.81	1.31
Stampfer-1992-4-4								35+ c/d									1.30	0.78	2.16
Stampfer-1992-5								Former smoker		Cholecystectomy							**1.06**	**0.94**	**1.18**
Stampfer-1992-6								Current smoker									**1.15**	**1.11**	**1.20**
Stampfer-1992-6-1								1-14 c/d									1.13	0.94	1.18
Stampfer-1992-6-2								15-24 c/d									1.21	1.04	1.42
Stampfer-1992-6-3								25-34 c/d									1.36	1.11	1.67
Stampfer-1992-6-4								35+ c/d									1.59	1.24	2.05
Kato-1992-1	1563324	Kato	1992	the Honolulu Heart Program	American	Male	1965-1990	Former smoker	Never smoking	Gallstones disease	histology or radiology	19.5	152831	7831	45-65+ y	RR	**1.10**	**0.90**	**1.50**
Kato-1992-2								Current smoker									**1.30**	**1.00**	**1.60**
Kato-1992-2-1								1-24 pack-years	Nonsmoker								1.00	0.80	1.30
Kato-1992-2-2								24-40 pack-years									1.30	1.00	1.70
Kato-1992-2-3								40+ pack-years									1.40	1.10	1.80
Murray-1994	8307429	Murray	1994	the Royal College of General Practitioners study	European	Female	1968-1987	smoker	Non-smoker	Gallstones disease	physician-diagnosed	19	NA	46000	NA	RR	**1.19**	**1.06**	**1.34**
Grodstein-1994-1	8041531	Grodstein	1994	the Nurses' Health Study cohort III	American	Female	1989-1991	Former smoker	Never smoking	Gallstones disease	NA	2	NA	96211	25-42 y	RR	**1.10**	**0.80**	**1.40**
Grodstein-1994-2								Current smoker									**1.30**	**1.00**	**1.70**
Misciagna-1996	8823575	Misciagna	1996	Epidemiology of cholelithiasis in southern Italy.II	European	Both	1985-1993	smoker	Non-smoker	Gallstones disease	ultrasonography	7	NA	1962	30-69 y	OR	**2.15**	**1.31**	**3.54**
Sahi-1998-1	9554603	Sahi	1998	the Harvard Alumni Health Study	American	Male	1962-1977	Former smoker	Never smoking	Gallstones disease	physician-diagnosed	13	174427	16414	32-74 y	RR	**1.28**	**0.89**	**1.85**
Sahi-1998-2								Current smoker									**1.47**	**1.13**	**1.92**
Sahi-1998-2-1								< 1pack/day									1.43	1.00	2.06
Sahi-1998-2-2								≥ 1pack/day									1.52	1.03	2.24
Syngal-1999	10075614	syngal	1999	the Nurses' Health Study cohort IV	American	Female	1988-1994	smoker	Non-smoker	Cholecystectomy	self-report and medica	6	NA	47153	30-55 y	RR	**1.05**	**0.92**	**1.20**
Boland-2002-1	11880221	Boland	2002	the Atherosclerosis Risk in Communities Study	American	Male	1987-1996	Former smoker	Never smoking	Gallstones disease	self-report and follow-up medical history phone interview	9	104872	5839	45-64 y	RR	**1.08**	**0.70**	**1.60**
Boland-2002-2								Current smoker									**1.13**	**0.70**	**1.80**
Boland-2002-3						Female		Former smoker						6934			**0.89**	**0.70**	**1.20**
Boland-2002-4								Current smoker									**0.78**	**0.60**	**1.10**
Yamada-2005	16024928	Yamada	2005	the Adult Health Study collected biennially	Asian	Both	1958-1998	smoker	Non-smoker	Cholelithiasis	medical records	23.6	282585	11982	13-98 y	RR	**1.19**	**1.02**	**1.40**
Gonzalez-2007-1	17103483	Gonzalez	2007	the General Practitioner Research Database in the UK	European	Both	1996	Former smoker	Never smoking	Gallstones disease	medical records	0.9	1253188	12353	20-79 y	OR	**1.18**	**0.99**	**1.41**
Gonzalez-2007-2								Current smoker									**1.05**	**0.94**	**1.19**
Katsika-2007-1	17908165	Katsika	2007	the Swedish Hospital Discharge and Causes of Death Registries for GD and GD-surgery [illegible]	European	Both	1970-2002	Former smoker	Never smoking	Gallstones disease	from questionnaires	~40	NA	58402	NA	RR	**1.15**	**0.95**	**1.39**
Katsika-2007-2								Current smoker									**0.99**	**0.78**	**1.27**
Liu-2009-1	19033524	Liu	2009	the Million Women Study recruited through the United Kingdom Breast Screening Service National Health Service	European	Female	1996-2005	Former smoker	Never smoking	Gallstones disease	ICD-10 diagnosis codes	6.1	NA	1290413	56 y	RR	**1.10**	**1.06**	**1.13**
Liu-2009-2								Current smoker									**1.22**	**1.18**	**1.26**
Liu-2009-2-1								1-9 c/d	Nonsmoker								1.12	1.05	1.19
Liu-2009-2-2								10-19 c/d									1.23	1.17	1.28
Liu-2009-2-3								20+ c/d									1.29	1.22	1.37
Halldestam-2009	19847878	Halldestam	2009	the general population who had been screened previously with ultrasonography and found to have no gallbladder stones	European	Both	NA	smoker	Non-smoker	Gallstones disease	ultrasonography	5	3025.8	503	58 y	OR	**1.18**	**0.43**	**3.25**
Etminan-2011	21502354	Etminan	2011	the IMS LifeLink Health Plan Claims Database	American	Female	1997-2009	smoker	Non-smoker	Cholecystectomy	ICD-9 codes	NA	2460094	2721014	~28.4 y	RR	**2.06**	**1.99**	**2.14**
Shabanzadeh-2016	27232657	Shabanzadeh	2016	the Danish MONICA I study and the 10-year follow-up of the cohort	European	Both	1982-1994	smoker	Non-smoker	Gallstones disease	ultrasonography	11.6	NA	2848	NA	RR	**0.84**	**0.63**	**1.12**

Figueiredo-2017-1	29221432	Figueiredo	2017	the Multiethnic Cohort study	American	Male	1993-2012	Former smoker	Never smoking	Gallstones disease	ICD-9 codes or hospitalization records	10.7	NA	64901	73.5 y	HR	**1.12**	**1.06**	**1.18**
Figueiredo-2017-1-1								Past 20-									1.09	1.01	1.16
Figueiredo-2017-1-2								Past 20+									1.16	1.07	1.27
Figueiredo-2017-2								Current smoker									**1.19**	**1.10**	**1.29**
Figueiredo-2017-2-1								Current 20-									1.16	1.04	1.30
Figueiredo-2017-2-2								Current 20+									1.22	1.09	1.37
Figueiredo-2017-3						Female		Former smoker						79508			**1.14**	**1.09**	**1.20**
Figueiredo-2017-3-1								Past 20-									1.10	1.04	1.16
Figueiredo-2017-3-2								Past 20+									1.30	1.17	1.44
Figueiredo-2017-4								Current smoker									**1.23**	**1.16**	**1.31**
Figueiredo-2017-4-1								Current 20-									1.17	1.08	1.28
Figueiredo-2017-4-2								Current 20+									1.37	1.23	1.53
Kim-2018	Abstract	Kim	2018	NA	Asian	Both	NA	smoker	Non-smoker	Gallstones disease	NA	NA	NA	24159	NA	HR	**1.18**	**0.79**	**1.75**

Table 2.2. Longitudinal studies of alcohol intake associated with gallstones risk

study ID	PMID	Author	Year	Subjects source	Country	Gender	Period	Expusure group	Control group	Disease	Diagnosis	Follow-up	Person years	Total (n_i)	Mean age	Effect	Effect size	LCI	UCI
Maclure-1989	2761600	Maclure	1989	the Nurses' Health Study cohort I	American	Female	1976-1984	Alcohol intake	Non-intake	Gallstones disease	follow-up questionnaires,self-reported or ultrasound records	4	None	88837	34-59 y	RR	**0.89**	**0.81**	**0.98**
Maclure-1989-1								0.1-1.49 g/d									1.00	0.80	1.30
Maclure-1989-2								1.5-4.99 g/d									0.90	0.80	1.20
Maclure-1989-3								5-14.99 g/d									0.80	0.60	1.00
Maclure-1989-4								15+ g/d									0.70	0.50	1.00
Sichieri-1990-1	2321626	Sichieri	1990	the first National Health and Nutrition Examination Survey	American	Male	1971-1984	Alcohol intake	Non-intake	Gallstones disease	self-report and medical records	9	None	4512	25-74 y	HR	**0.89**	**0.69**	**1.14**
Sichieri-1990-2						Female								6041			**0.98**	**0.81**	**1.17**
Kato-1992	1563324	Kato	1992	the Honolulu Heart Program	American	Male	1965-1990	Alcohol intake	Non-intake	Gallstones disease	histology or radiology	19.5	152831	7831	45-65+ y	RR	**0.80**	**0.60**	**1.00**
Moerman-1994	8055126	Moerman	1994	The Zutphen Study, a cross-cultural longitudinal study on diet, risk factors, and complications of atherosclerosis	European	Male	1960-1985	Alcohol intake	Non-intake	Gallstones disease	self-report and medical records	25	None	860	63 y	HR	**0.70**	**0.44**	**1.11**
Moerman-1994-1								1-10 g/d									0.70	0.40	1.30
Moerman-1994-2								10+ g/d									0.70	0.30	1.80
Misciagna-1996	8823575	Misciagna	1996	Epidemiology of cholelithiasis in southern Italy.II	European	Both	1985-1993	Alcohol intake	Non-intake	Gallstones disease	ultrasonography	7	NA	1962	30-69 y	OR	**0.71**	**0.54**	**0.92**
Misciagna-1996-1								Wine intake									**0.79**	**0.61**	**1.03**
Misciagna-1996-1-1								0.25 L/d									0.94	0.69	1.27
Misciagna-1996-1-2								0.5 L/d									0.58	0.31	1.08
Misciagna-1996-1-3								1+ L/d									0.28	0.10	0.76
Misciagna-1996-2								Beer intake									**1.11**	**0.60**	**2.05**
Misciagna-1996-3								Spirits intake	< 1 time/week								**0.78**	**0.45**	**1.34**
Misciagna-1996-3-1								1-5 times/week									0.68	0.36	1.30
Misciagna-1996-3-2								5+ times/week									1.11	0.38	3.19
Leitzmann-1999-1	10371403	Leitzmann	1999	The Health Professionals Follow-up Study,between 40-75 years of age,returned a questionnaire by mail that concerned diet, medical history, and medications.	American	Male	1986-1996	Alcohol intake I	Abstainers	Gallstones disease	follow-up questionnaires,self-reported or ultrasound records	10	404166	46006	54 y	RR	**0.83**	**0.76**	**0.91**
Leitzmann-1999-1-1								0.1-1.49 g/d									0.97	0.76	1.22
Leitzmann-1999-1-2								1.5-4.99 g/d									0.95	0.79	1.14
Leitzmann-1999-1-3								5-14.99 g/d									0.83	0.69	0.99
Leitzmann-1999-1-4								15-29.9 g/d									0.75	0.60	0.93
Leitzmann-1999-1-5								30+ g/d									0.64	0.50	0.81
Leitzmann-1999-2								Alcohol intake II									**0.82**	**0.74**	**0.91**
Leitzmann-1999-2-1								1-2 days/week									0.96	0.82	1.11
Leitzmann-1999-2-2								3-4 days/week									0.79	0.65	0.97
Leitzmann-1999-2-3								5-7 days/week									0.69	0.58	0.82
Leitzmann-1999-3								Wine intake									**0.86**	**0.74**	**1.01**
Leitzmann-1999-3-1								< 15 grams/day									0.98	0.81	1.17
Leitzmann-1999-3-2								≥ 15 grams/day									0.59	0.43	0.81
Leitzmann-1999-4								Beer intake									**0.75**	**0.62**	**0.91**
Leitzmann-1999-4-1								< 15 grams/day									0.79	0.62	1.00
Leitzmann-1999-4-2								≥ 15 grams/day									0.68	0.49	0.92
Leitzmann-1999-5								Liquor intake									**0.79**	**0.68**	**0.93**
Leitzmann-1999-5-1								< 15 grams/day									0.84	0.68	1.05
Leitzmann-1999-5-2								≥ 15 grams/day									0.75	0.61	0.94

Boland-2002-1								Former drunker									**1.52**	**0.90**	**2.70**
Boland-2002-2								Current drinking									**0.98**	**0.71**	**1.36**
Boland-2002-2-1						Male		< 1 drink/week						5839			1.16	0.60	2.20
Boland-2002-2-2								1-7 drinks/week									1.06	0.60	1.90
Boland-2002-2-3	11880221	Boland	2002	the Atherosclerosis Risk in Communities Study	American		1987-1996	7+ drinks/week	Never drinking	Gallstones disease	self-report and follow-up medical history phone interview	9	104872		45-64 y	RR	0.84	0.50	1.50
Boland-2002-3								Former drunker									**0.76**	**0.50**	**1.10**
Boland-2002-4								Current drinking									**0.88**	**0.73**	**1.06**
Boland-2002-4-1						Female		< 1 drink/week						6934			1.02	0.80	1.40
Boland-2002-4-2								1-7 drinks/week									0.74	0.50	1.10
Boland-2002-4-3								7+ drinks/week									0.53	0.30	0.90
Leitzmann-2003-1								Alcohol intake I									**0.70**	**0.65**	**0.75**
Leitzmann-2003-1-1								0.1-4.9 g/d									0.81	0.73	0.89
Leitzmann-2003-1-2							1980-1986	5.0-14.9 g/d		Gallstones							0.67	0.59	0.76
Leitzmann-2003-1-3								15-29.9 g/d									0.56	0.45	0.70
Leitzmann-2003-1-4								30+ g/d									0.46	0.36	0.59
Leitzmann-2003-2								Alcohol intake II				20	1393256	80898			**0.86**	**0.83**	**0.90**
Leitzmann-2003-2-1								0.1-4.9 g/d									0.95	0.89	1.00
Leitzmann-2003-2-2							1980-2000	5.0-14.9 g/d		Cholecystectomy							0.86	0.80	0.93
Leitzmann-2003-2-3								15-29.9 g/d									0.80	0.72	0.89
Leitzmann-2003-2-4								30-49.9 g/d									0.67	0.57	0.78
Leitzmann-2003-2-5								50+ g/d									0.62	0.49	0.79
Leitzmann-2003-3								frequency of intake									0.88	0.83	0.93
Leitzmann-2003-3-1	12885719	Leitzmann	2003	the Nurses' Health Study cohort V	American	Female		1-2 days/week	Non-intake		self-report and medical records				30-55 y	RR	0.94	0.86	1.01
Leitzmann-2003-3-2								3-4 days/week									0.88	0.77	0.99
Leitzmann-2003-3-3								5-6 days/week									0.87	0.76	0.99
Leitzmann-2003-3-4								7 days/week									0.73	0.63	0.84
Leitzmann-2003-4								Wine intake									**0.88**	**0.81**	**0.94**
Leitzmann-2003-4-1								< 15 grams/day									0.89	0.82	0.98
Leitzmann-2003-4-2							1986-2000	≥ 15 grams/day		cholecystectomy		14	NA	58374			0.79	0.65	0.96
Leitzmann-2003-5								Beer intake									**0.88**	**0.76**	**1.02**
Leitzmann-2003-5-1								< 15 grams/day									1.03	0.87	1.23
Leitzmann-2003-5-2								≥ 15 grams/day									0.57	0.43	0.76
Leitzmann-2003-6								Liquor intake									**0.84**	**0.75**	**0.94**
Leitzmann-2003-6-1								< 15 grams/day									0.90	0.79	1.03
Leitzmann-2003-6-2								≥ 15 grams/day									0.72	0.59	0.87
Yamada-2005	16024928	Yamada	2005	the Adult Health Study collected biennially	Asian	Both	1958-1998	Ever drinking	Never drinking	Cholelithiasis	medical records	23.6	282585	11982	13-98 y	RR	**1.02**	**0.89**	**1.17**
Gonzalez-2007								Alcohol intake									**0.79**	**0.73**	**0.86**
Gonzalez-2007-1	17103483	Gonzalez	2007	the General Practitioner Research Database in the UK	European	Both	1996	2-20 U	< 2 U	Gallstones disease	medical records	0.9	1253188	12353	20-79 y	OR	0.81	0.73	0.91
Gonzalez-2007-2								20-35 U									0.69	0.49	0.97
Gonzalez-2007-3								35+ U									0.59	0.35	0.98
Katsika-2007-1	17908165	Katsika	2007	the Swedish Hospital Discharge and Causes of Death Registries for GD and GD-surgery	European	Both	1970-2002	Moderate consumption	Never consumption	Gallstones disease	from questionnaires	~40	NA	58402	NA	RR	**0.93**	**0.83**	**1.04**
Katsika-2007-2								High consumption									**0.57**	**0.53**	**0.62**
Liu-2009								Alcohol intake									**0.76**	**0.75**	**0.77**
Liu-2009-1								1-2 units/week									0.92	0.88	0.94
Liu-2009-2	19033524	Liu	2009	the Million Women Study recruited through the United Kingdom Breast Screening Service National Health Service	European	Female	1996-2005	3-6 units/week	Abstainers	Gallstones disease	ICD-10 diagnosis codes	6.1	NA	1290413	56 y	RR	0.83	0.83	0.84
Liu-2009-3								7-14 units/week									0.68	0.67	0.68
Liu-2009-4								≥ 15 units/week									0.54	0.52	0.57
Halldestam-2009	19847878	Halldestam	2009	the general population who had been screened previously with ultrasonography and found to have	European	Both	NA	Alcohol consumption	Never consumption	Gallstones disease	ultrasonography	5	3025.8	503	58 y	OR	**0.29**	**0.09**	**0.98**
Banim-2011-1								Alcohol intake									**0.89**	**0.60**	**1.33**
Banim-2011-1-1								0.1-7 units/week									1.10	0.58	2.14
Banim-2011-1-2						Male		7-14 units/week						11188	69.1 y		1.20	0.58	2.46
Banim-2011-1-3								14-21 units/week									0.58	0.21	1.58
Banim-2011-1-4	21623190	Banim	2011	the European Prospective Investigation of Cancer-Norfolk (EPIC-Norfolk)	European		1993-2007	21+ units/week	Non-intake	Gallstones disease	histology or radiology,ICD-10 diagnosis codes	14	279504			HR	0.46	0.17	1.25
Banim-2011-2								Alcohol intake									**0.93**	**0.71**	**1.22**
Banim-2011-2-1								0.1-7 units/week									1.01	0.70	1.46
Banim-2011-2-2						Female		7-14 units/week						13075	65.9 y		0.72	0.42	1.24
Banim-2011-2-3								14-21 units/week									0.99	0.48	2.05
Banim-2011-2-4								21+ units/week									1.10	0.39	3.11

Shabanzadeh-2016	27232657	Shabanzadeh	2016	the Danish MONICA I study and the 10-year follow-up of the cohort	European	Both	1982-1994	Alcohol consumption	Non-consumption	Gallstones disease	ultrasonography	11.6	NA	2848	30-60 y	RR	**0.99**	**0.98**	**1.00**
Shabanzadeh-2017-1	28704597		2017			Male								1227	43.5 y		1.00	0.98	1.02
Shabanzadeh-2017-2						Female								1139	42.8 y		0.94	0.90	0.98
Figueiredo-2017-1	29221432	Figueiredo	2017	the Multiethnic Cohort study	American	Male	1993-2012	Alcohol intake	Non-intake	Gallstones disease	ICD-9 codes or hospitalization records	10.7	NA	64901	73.5 y	HR	**0.89**	**0.85**	**0.94**
Figueiredo-2017-1-1								< 24 g/d									0.92	0.86	0.97
Figueiredo-2017-1-2								24-48 g/d									0.85	0.77	0.94
Figueiredo-2017-1-3								48 + g/d									0.86	0.76	0.97
Figueiredo-2017-2						Female		Alcohol intake						79508			**0.86**	**0.82**	**0.90**
Figueiredo-2017-2-1								< 24 g/d									0.86	0.82	0.91
Figueiredo-2017-2-2								24-48 g/d									0.80	0.69	0.93
Figueiredo-2017-2-3								48 + g/d									0.92	0.74	1.14
Kim-2018	Abstract	Kim	2018	NA	Asian	Both	NA	Alcohol intake	Non-intake	Gallstones disease	NA	NA	NA	24159	NA	HR	**0.81**	**0.56**	**1.17**

Table 2.3. Longitudinal studies of coffee consumption (caffeine intake) associated with gallstones risk

study ID	PMID	Author	Year	Subjects source	Country	Gender	Period	Expusure group	Control group	Disease	Diagnosis	Follow-up	Person years	Total (n_i)	Mean age	Effect	Effect size	LCI	UCI
Misciagna-1996	8823575	Misciagna	1996	Epidemiology of cholelithiasis in southern Italy.II	European	Both	1985-1993	Coffee consumption	Non-consumption	Gallstones disease	ultrasonography	7	NA	1962	30-69 y	OR	**0.62**	**0.40**	**0.98**
Leitzmann-1999-1	10367821	Leitzmann	1999	The Health Professionals Follow-up Study	American	Male	1986-1996	Coffee consumption	Non-consumption	Gallstones disease	follow-up questionnaires,self-reported or ultrasound records	10	404166	46008	40-75 y	RR	**0.73**	**0.60**	**0.89**
Leitzmann-1999-1-1								≤ 1 cup/day									0.88	0.67	1.14
Leitzmann-1999-1-2								2-3 cups/day									0.60	0.42	0.86
Leitzmann-1999-1-3								≥ 4 cups/day									0.55	0.33	0.92
Leitzmann-1999-2								Caffeine intake	Little-intake								**0.88**	**0.80**	**0.96**
Leitzmann-1999-2-1								26-100 mg/d	≤ 25 mg/d								0.97	0.81	1.17
Leitzmann-1999-2-2								101-200 mg/d									0.87	0.72	1.06
Leitzmann-1999-2-3								201-400 mg/d									0.92	0.76	1.10
Leitzmann-1999-2-4								401-800 mg/d									0.80	0.64	0.99
Leitzmann-1999-2-5								800+ mg/d									0.55	0.35	0.87
Leitzmann-1999-3								Caffeinated coffee intake	Non-intake								**0.80**	**0.73**	**0.89**
Leitzmann-1999-3-1								≤ 1 cup/day									0.87	0.75	1.00
Leitzmann-1999-3-2								2-3 cups/day									0.79	0.67	0.94
Leitzmann-1999-3-3								≥ 4 cups/day									0.67	0.53	0.84
Leitzmann-1999-4								Decaffeinated coffee intake									**1.05**	**0.95**	**1.17**
Leitzmann-1999-4-1								≤ 1 cup/day									1.05	0.92	1.20
Leitzmann-1999-4-2								2-3 cups/day									1.05	0.86	1.27
Leitzmann-1999-4-3								≥ 4 cups/day									1.08	0.79	1.47
Leitzmann-1999-5								Tea intake	Decaffeinated drinks intake								**1.10**	**0.98**	**1.23**
Leitzmann-1999-5-1								≤ 1 cup/day									1.13	0.99	1.29
Leitzmann-1999-5-2								2-3 cups/day									0.87	0.66	1.15
Leitzmann-1999-5-3								≥ 4 cups/day									1.42	0.92	2.18
Leitzmann-2002								Coffee consumption	Non-intake								**0.89**	**0.86**	**0.91**
Leitzmann-2002-1	12454839	Leitzmann	2002	the Nurses' Health Study cohort VI	American	Female	1980-2000	Caffeinated coffee intake		Cholecystectomy	self-report and medical records	20	1389936	80898	30-55 y	RR	**0.85**	**0.82**	**0.87**
Leitzmann-2002-1-1								≤ 1 cup/day									0.92	0.87	0.98
Leitzmann-2002-1-2								2-3 cups/day									0.82	0.78	0.87
Leitzmann-2002-1-3								≥ 4 cups/day									0.77	0.71	0.83
Leitzmann-2002-2								Decaffeinated coffee intake									**0.99**	**0.94**	**1.04**
Leitzmann-2002-2-1								≤ 1 cup/day									1.01	0.95	1.08
Leitzmann-2002-2-2								2-3 cups/day									0.96	0.88	1.05
Leitzmann-2002-2-3								≥ 4 cups/day									0.93	0.79	1.09
Leitzmann-2002-3								Tea intake	Decaffeinated drinks intake								**1.07**	**1.03**	**1.11**
Leitzmann-2002-3-1								≤ 1 cup/day									1.07	1.02	1.13
Leitzmann-2002-3-2								2-3 cups/day									1.06	0.98	1.14
Leitzmann-2002-3-3								≥ 4 cups/day									1.13	1.00	1.28
Banim-2011-1	Abstract	Banim	2011	the European Prospective Investigation of Cancer-Norfolk (EPIC-Norfolk)	European	Male	1993-2007	Caffeinated coffee consumption	Non-consumption	Gallstones disease	histology or radiology,ICD-10 diagnosis codes	14	NA	11281	64.2 y	HR	**0.58**	**0.38**	**0.90**
Banim-2011-2						Female								14358	66.5 y		**0.84**	**0.60**	**1.17**

Nordenvall-2014-1	25245628	Nordenvall	2014	the Swedish Mammography Cohort and the Cohort of Swedish Men.	European	Male	1998-2011	Coffee consumption	< 2 cups/day	Cholecystectomy	ICD-9, ICD-10 diagnosis codes	11	905933	40936	NA	HR	**0.99**	**0.88**	**1.12**
Nordenvall-2014-1-1								2-3 cups/day									1.04	0.86	1.25
Nordenvall-2014-1-2								4-5 cups/day									0.96	0.78	1.18
Nordenvall-2014-1-3								≥ 6 cups/day									0.96	0.75	1.24
Nordenvall-2014-2						Female		Coffee consumption						30989			**0.75**	**0.67**	**0.84**
Nordenvall-2014-2-1								2-3 cups/day									0.88	0.74	1.03
Nordenvall-2014-2-2								4-5 cups/day									0.67	0.55	0.82
Nordenvall-2014-2-3								≥ 6 cups/day									0.58	0.44	0.78
Shabanzadeh-2016	27232657	Shabanzadeh	2016	the Danish MONICA I study and the 10-year follow-up of the cohort	European	Both	1982-1994	Coffee consumption	Non-consumption	Gallstones disease	ultrasonography	11.6	NA	2848	30-60 y	RR	**1.00**	**0.97**	**1.04**
Shabanzadeh-2017-1	28704597		2017			Male								1227	43.5 y		0.98	0.92	1.05
Shabanzadeh-2017-2						Female								1139	42.8 y		0.97	0.90	1.05
Nordestgaard-2019	31486166	Nordestgaard	2019	the Copenhagen General Population Study (CGPS) & the Copen hagen City Heart Study (CCHS)	European	Both	2003-2014	Coffee intake	Non-intake	Gallstones disease	reported by practitioners and records from hospitals, ICD 10	8	NA	104493	50-60y	RR	**0.86**	**0.79**	**0.95**
Nordestgaard-2019-1								0.1-3 cups/day									0.93	0.81	1.07
Nordestgaard-2019-2								3.1-6 cups/day									0.83	0.71	0.97
Nordestgaard-2019-3								≥ 6 cups/day									0.77	0.61	0.96

Table 2.4. Longitudinal studies of tea consumption associated with gallstones risk

**study ID**	**PMID**	**Author**	**Year**	**Subjects source**	**Country**	**Gender**	**Period**	**Expusure group**	**Control group**	**Disease**	**Diagnosis**	**Follow-up**	**Person years**	**Total (n_i)**	**Mean age**	**Effect**	**Effect size**	**LCI**	**UCI**
Leitzmann-1999-1	10367821	Leitzmann	1999	The Health Professionals Follow-up Study	American	Male	1986-1996	Tea intake	Decaffeinated drinks intake	Gallstones disease	follow-up questionnaires,self-reported or ultrasound records	10	404166	46008	40-75 y	RR	**1.10**	**0.98**	**1.23**
Leitzmann-1999-1-1								≤ 1 cup/day									1.13	0.99	1.29
Leitzmann-1999-1-2								2-3 cups/day									0.87	0.66	1.15
Leitzmann-1999-1-3								≥ 4 cups/day									1.42	0.92	2.18
Leitzmann-2002-1	12454839	Leitzmann	2002	the Nurses' Health Study cohort VI	American	Female	1980-2000	Tea intake	Decaffeinated drinks intake	Cholecystectomy	self-report and medical records	20	1389936	80898	30-55 y	RR	**1.07**	**1.03**	**1.11**
Leitzmann-2002-1-1								≤ 1 cup/day									1.07	1.02	1.13
Leitzmann-2002-1-2								2-3 cups/day									1.06	0.98	1.14
Leitzmann-2002-1-3								≥ 4 cups/day									1.13	1.00	1.28

Table 3. Characteristic information for instrumental variables of the addictive behaviors

Exposure	Study	Source	Sample Size (N)	Sig. SNPs (n)	Variance Explain	F-value	PMID
Lifetime smoking	Evidence for causal effects of lifetime smoking on risk for depression and schizophrenia: a Mendelian randomisation study (Psychol Med, 2020)	UK Biobank	462,690	126	1.31%	48.35	31689377
Ever smoking	Association studies of up to 1.2 million individuals yield new insights into the genetic etiology of tobacco and alcohol use (Nat Genet, 2019)	European-ancestry	1,232,091	358	1.33%	45.72	30643251
Current smoking			337,334	48	1.20%	85.64	
Smoking cessation			547,219	23	0.24%	56.31	
Common alcohol use			941,280	94	0.64%	64.25	
Problematic alcohol use	Genome-wide meta-analysis of problematic alcohol use in 435,563 individuals yields insights into biology and relationships with other traits (Nat Neurosci, 2020)	European-ancestry	435,563	34	0.56%	72.35	32451486
Caffeine intake	Associations of Observational and Genetically Determined Caffeine Intake With Coronary Artery Disease and Diabetes Mellitus (J Am Heart Assoc, 2020)	UK Biobank	362,316	55	1.72%	115.49	33287642
Coffee consumption			373,522	24	0.59%	92.67	
Tea consumption			395,866	24	0.46%	75.58	

Lifetime smoking: an addictive behavior which captures smoking duration, heaviness and cessation; Smoking initiation: indicating whether an individual had ever smoked regularly; Current smoking: Heaviness of smoking was measured with cigarettes per day; Smoking cessation: contrasting current-smokers vs.former-smokers; Alcohol drinking: an addictive behavior with drinks per week; Problematic alcohol use (PAU): both AUD and AUDIT-P as PAU (AUD: alcohol use and alcohol use disorder, AUDIT-P: a measure of problematic drinking, such as the Alcohol Use Disorders Identification Test-Problems); Caffeine intake: calculating the number of cups of coffee or tea multiplied by the caffeine content per cup; Coffee consumption: daily coffee intake were assessed by asking participants "How many cups of coffee do you drink each day (include decaffeinated coffee)"; Tea consumption: daily tea intake were assessed by asking participants "How many cups of tea do you drink each day (include black and green tea)".

Table 4.1. Characteristics of genetic instruments associated with lifetime smoking

SNP	Chromosome	beta	se	p-value	effect_allele	other_allele	eaf	R^2
rs1193237	1	-0.011000	0.002000	2.80E-08	G	C	0.439	0.000
rs4949465	1	-0.017000	0.003000	1.70E-08	T	C	0.870	0.009
rs549845	1	0.016000	0.002000	8.30E-14	G	A	0.301	0.000
rs1933270	1	0.013000	0.002000	1.50E-10	T	G	0.364	0.003
rs7528604	1	0.014000	0.002000	5.70E-12	G	A	0.566	0.012
rs11210229	1	0.017000	0.002000	2.00E-16	A	G	0.384	0.001
rs7553348	1	0.014000	0.002000	5.20E-12	G	A	0.438	0.019
rs10922907	1	0.015000	0.002000	3.00E-13	A	T	0.451	0.002
rs1931263	1	-0.011000	0.002000	4.00E-08	G	T	0.510	0.001
rs7519626	1	0.012000	0.002000	1.20E-08	C	T	0.324	0.012
rs9435340	1	0.012000	0.002000	1.20E-08	T	A	0.344	0.008
rs10918701	1	0.012000	0.002000	2.10E-08	G	A	0.372	0.000
rs2867112	2	0.021000	0.003000	4.80E-15	T	G	0.835	0.000
rs6741228	2	0.011000	0.002000	1.60E-08	T	C	0.433	0.000
rs62135536	2	0.035000	0.006000	8.00E-10	C	T	0.968	0.000
rs7569203	2	-0.016000	0.002000	7.40E-13	A	C	0.689	0.007
rs13016665	2	-0.012000	0.002000	1.80E-09	C	A	0.577	0.004
rs4671357	2	-0.014000	0.002000	1.10E-11	T	C	0.519	0.008
rs359243	2	-0.013000	0.002000	9.50E-10	T	C	0.393	0.001
rs2678670	2	0.013000	0.002000	3.10E-10	A	T	0.486	0.000
rs62155874	2	-0.024000	0.003000	5.20E-16	A	G	0.873	0.005
rs3811038	2	-0.014000	0.002000	8.90E-10	T	C	0.724	0.000
rs2890772	2	-0.020000	0.002000	2.10E-22	G	T	0.413	0.003
rs62175972	2	0.031000	0.006000	1.70E-08	T	C	0.966	0.000
rs3769949	2	-0.012000	0.002000	2.50E-09	T	A	0.528	0.000

rs13009008	2	0.012000	0.002000	4.60E-09	A	G	0.328	0.000
rs4473348	2	-0.015000	0.002000	6.40E-11	A	T	0.250	0.010
rs12623702	2	-0.014000	0.002000	7.70E-12	A	G	0.613	0.000
rs6779302	3	-0.013000	0.002000	1.20E-09	G	T	0.633	0.001
rs6778080	3	0.016000	0.002000	1.30E-12	T	C	0.267	0.000
rs775758	3	0.012000	0.002000	1.10E-08	A	T	0.433	0.001
rs421983	3	0.013000	0.002000	3.30E-10	T	C	0.519	0.008
rs326341	3	0.014000	0.002000	1.20E-11	G	A	0.525	0.001
rs73220544	3	-0.016000	0.003000	1.50E-08	A	C	0.842	0.000
rs9842947	3	-0.013000	0.002000	3.10E-09	C	T	0.326	0.009
rs624833	4	0.013000	0.002000	6.60E-10	T	G	0.695	0.003
rs61796681	4	-0.019000	0.004000	4.20E-08	A	T	0.912	0.000
rs317021	4	-0.017000	0.003000	1.10E-10	T	A	0.814	0.016
rs72678864	4	0.018000	0.003000	1.60E-11	G	A	0.829	0.000
rs17576594	4	0.016000	0.002000	1.70E-12	G	A	0.724	0.001
rs11948770	5	-0.015000	0.002000	4.90E-10	T	C	0.768	0.005
rs71627581	5	0.019000	0.003000	1.60E-09	G	A	0.889	0.001
rs10052591	5	0.012000	0.002000	2.10E-09	T	C	0.573	0.009
rs2080870	5	0.012000	0.002000	4.90E-08	A	T	0.258	0.003
rs4571506	5	0.011000	0.002000	1.50E-08	C	T	0.540	0.002
rs4957528	5	-0.015000	0.002000	4.20E-09	A	C	0.208	0.003
rs329120	5	0.014000	0.002000	6.30E-12	C	T	0.581	0.007
rs986391	5	0.016000	0.002000	9.40E-15	G	A	0.367	0.005
rs13153393	5	-0.020000	0.003000	2.50E-10	A	G	0.884	0.004
rs245774	5	-0.013000	0.002000	7.40E-09	A	G	0.272	0.000
rs6935954	6	0.014000	0.002000	8.20E-12	A	G	0.421	0.000
rs2254710	6	0.013000	0.002000	3.50E-08	C	A	0.236	0.000
rs2894808	6	-0.022000	0.004000	3.50E-09	T	A	0.922	0.001

rs12202536	6	-0.012000	0.002000	2.80E-09	A	G	0.513	0.000
rs7766610	6	0.018000	0.003000	2.20E-12	C	A	0.183	0.000
rs1922018	7	0.014000	0.002000	3.00E-12	C	T	0.364	0.001
rs10226228	7	-0.016000	0.002000	2.00E-15	A	G	0.630	0.000
rs11768481	7	0.013000	0.002000	9.90E-10	C	A	0.666	0.002
rs6962772	7	0.016000	0.003000	7.80E-09	A	G	0.846	0.002
rs10282292	7	0.013000	0.002000	5.90E-10	C	T	0.362	0.003
rs2401924	7	0.015000	0.002000	2.70E-14	G	C	0.502	0.002
rs7807019	7	-0.015000	0.002000	6.70E-14	A	G	0.540	0.004
rs6957896	7	-0.011000	0.002000	4.50E-08	C	T	0.503	0.001
rs4731925	7	-0.012000	0.002000	2.60E-08	C	T	0.316	0.013
rs35169606	8	0.013000	0.002000	1.20E-09	T	G	0.612	0.000
rs11783093	8	0.023000	0.003000	1.20E-16	C	T	0.839	0.003
rs2062882	8	-0.012000	0.002000	1.10E-08	G	A	0.587	0.002
rs72674867	8	0.013000	0.002000	3.80E-08	A	T	0.765	0.000
rs4543592	9	-0.012000	0.002000	4.50E-10	T	C	0.520	0.000
rs7039819	9	0.013000	0.002000	5.10E-10	G	A	0.427	0.001
rs1246265	9	-0.013000	0.002000	4.20E-09	T	C	0.305	0.002
rs1221148	9	0.013000	0.002000	7.30E-11	C	G	0.587	0.005
rs13296519	9	-0.014000	0.002000	8.10E-12	G	T	0.606	0.000
rs113382419	9	-0.041000	0.003000	3.00E-37	C	A	0.889	0.003
rs11255908	10	-0.015000	0.002000	2.30E-10	T	G	0.743	0.001
rs2675638	10	0.012000	0.002000	1.30E-09	G	A	0.581	0.001
rs10823968	10	0.012000	0.002000	2.10E-08	A	T	0.633	0.000
rs17553262	10	-0.018000	0.003000	5.30E-09	A	C	0.885	0.000
rs7077678	10	0.012000	0.002000	2.60E-09	C	T	0.623	0.000
rs12244388	10	-0.019000	0.002000	1.40E-19	G	A	0.661	0.006
rs3896224	10	0.014000	0.002000	1.10E-11	A	G	0.585	0.001

rs34866095	11	-0.012000	0.002000	1.20E-08	A	G	0.686	0.000
rs75742406	11	0.014000	0.002000	1.30E-09	G	A	0.739	0.001
rs17309874	11	-0.016000	0.002000	9.70E-13	G	A	0.740	0.000
rs4391802	11	0.015000	0.002000	1.40E-11	A	G	0.707	0.000
rs112282219	11	-0.033000	0.005000	3.80E-11	G	A	0.959	0.000
rs9919670	11	-0.022000	0.002000	7.60E-27	G	A	0.612	0.006
rs74086911	12	0.021000	0.004000	2.10E-08	G	A	0.925	0.000
rs7297175	12	-0.012000	0.002000	6.60E-09	T	C	0.431	0.000
rs10879871	12	-0.014000	0.002000	5.00E-11	T	G	0.343	0.006
rs12831617	12	-0.013000	0.002000	1.90E-08	C	T	0.764	0.002
rs7333559	13	0.015000	0.002000	3.20E-10	G	A	0.212	0.007
rs6562474	13	0.012000	0.002000	1.00E-08	C	G	0.651	
rs860326	14	0.012000	0.002000	2.70E-09	C	T	0.428	0.000
rs7155595	14	-0.013000	0.002000	2.50E-09	A	C	0.674	0.003
rs3742365	14	-0.016000	0.002000	2.50E-14	T	C	0.595	0.000
rs35175834	15	-0.024000	0.002000	4.60E-22	G	A	0.788	0.000
rs28485305	15	0.012000	0.002000	2.60E-08	C	T	0.631	0.001
rs8042849	15	0.028000	0.002000	1.80E-39	C	T	0.342	0.002
rs8042134	15	-0.014000	0.002000	1.30E-12	T	G	0.541	0.001
rs6598539	15	-0.012000	0.002000	4.50E-09	T	C	0.489	0.001
rs11861214	16	0.014000	0.002000	2.00E-08	G	T	0.784	0.000
rs12708665	16	-0.013000	0.002000	3.50E-09	A	G	0.285	0.002
rs57611503	16	0.011000	0.002000	4.00E-08	G	A	0.485	0.000
rs889398	16	0.013000	0.002000	6.30E-11	C	T	0.588	0.002
rs60952428	16	0.019000	0.003000	3.00E-08	T	C	0.909	0.000
rs1050847	16	0.011000	0.002000	1.40E-08	C	T	0.426	0.003
rs369230	16	-0.013000	0.002000	1.80E-09	G	T	0.308	0.012
rs8614	17	-0.017000	0.003000	1.80E-10	C	A	0.817	0.000

rs732083	17	0.012000	0.002000	1.50E-08	G	A	0.333	0.001
rs9904288	17	0.012000	0.002000	3.10E-08	T	C	0.708	0.001
rs67596067	17	-0.013000	0.002000	1.20E-09	G	A	0.649	0.001
rs71367545	18	-0.015000	0.002000	1.40E-09	G	A	0.791	0.000
rs62098013	18	-0.012000	0.002000	4.10E-09	G	A	0.640	0.005
rs12967855	18	0.012000	0.002000	3.10E-08	A	G	0.331	0.003
rs76608582	19	0.031000	0.005000	3.20E-10	C	A	0.953	0.005
rs35343344	19	0.013000	0.002000	8.80E-09	C	A	0.733	
rs4814873	20	0.014000	0.002000	2.90E-09	C	T	0.767	0.000
rs6119897	20	-0.018000	0.002000	3.60E-15	G	A	0.762	0.005
rs12481282	20	-0.013000	0.002000	7.80E-09	G	C	0.722	0.002
rs348809	20	-0.012000	0.002000	1.30E-08	A	G	0.348	0.005
rs6011779	20	0.028000	0.003000	2.30E-27	C	T	0.191	0.001
rs2838834	21	-0.013000	0.002000	6.30E-10	C	T	0.699	0.002
rs147412694	21	-0.017000	0.003000	2.90E-09	G	A	0.850	
rs202645	22	-0.015000	0.002000	3.90E-09	A	G	0.203	0.000
rs136233	22	-0.014000	0.003000	1.80E-08	A	G	0.809	

Table 4.2. Characteristics of genetic instruments associated with ever smoking

SNP	Chromosome	beta	se	p-value	effect_allele	other_allele	eaf	R^2
rs12130857	1	-0.018003	0.002720	3.65E-11	A	G	0.325	0.001
rs301807	1	0.018014	0.002573	2.50E-12	G	A	0.570	0.001
rs3820277	1	-0.018837	0.002552	1.57E-13	T	G	0.526	0.003
rs1889571	1	0.022180	0.003776	4.19E-09	G	T	0.131	0.002
rs10914684	1	-0.015804	0.002722	6.32E-09	A	G	0.324	0.000
rs2637869	1	0.018220	0.002788	6.54E-11	A	G	0.297	0.009
rs12755632	1	-0.015405	0.002740	1.93E-08	G	A	0.316	0.001
rs951740	1	0.029541	0.002632	3.82E-29	A	G	0.625	0.000
rs925524	1	0.015557	0.002808	2.94E-08	G	A	0.710	0.003
rs12022778	1	0.026825	0.003179	3.18E-17	C	A	0.202	0.008
rs11587399	1	-0.017805	0.003076	7.25E-09	T	A	0.221	0.023
rs4912332	1	0.014121	0.002549	2.94E-08	T	C	0.491	0.000
rs1937443	1	0.020436	0.002569	1.79E-15	G	C	0.563	0.000
rs1022528	1	0.017402	0.002682	8.48E-11	A	G	0.344	0.003
**rs12740789**	1	-0.028497	0.003331	1.18E-17	A	G	0.178	0.036
rs10789369	1	-0.023448	0.002618	3.39E-19	G	A	0.615	0.006
rs1514176	1	-0.019300	0.002581	7.67E-14	A	G	0.580	0.016
rs10873871	1	0.017452	0.003145	2.82E-08	G	A	0.207	0.000
rs11162019	1	-0.015495	0.002650	5.06E-09	T	C	0.363	0.000
rs1008078	1	0.022817	0.002599	1.63E-18	T	C	0.402	0.000
rs1935571	1	-0.015720	0.002550	6.99E-10	G	T	0.480	0.000
rs12027999	1	-0.024359	0.003921	5.33E-10	C	T	0.120	0.012
rs45444697	1	0.019690	0.003117	2.72E-10	G	C	0.212	0.000
rs2901785	1	-0.017308	0.002563	1.47E-11	A	G	0.446	0.000
rs147052174	1	0.062309	0.009827	2.30E-10	T	G	0.017	0.002

rs35656245	1	0.015946	0.002850	2.23E-08	A	G	0.276	0.000
rs12739243	1	-0.021252	0.003071	4.45E-12	C	T	0.221	0.002
rs12563365	1	0.016559	0.002564	1.05E-10	A	G	0.556	0.009
rs876793	1	-0.017925	0.002737	5.69E-11	C	T	0.349	0.001
rs114976176	2	-0.015514	0.002668	6.04E-09	C	A	0.352	0.001
rs62106258	2	-0.045498	0.006000	3.33E-14	C	T	0.047	0.004
rs6731872	2	0.031598	0.003361	5.35E-21	G	T	0.826	0.004
rs1022376	2	-0.014745	0.002611	1.66E-08	C	T	0.516	0.005
rs61533748	2	0.017436	0.002620	2.82E-11	C	T	0.384	0.010
rs72790288	2	-0.045532	0.007696	3.28E-09	A	G	0.028	0.000
rs2710634	2	-0.017761	0.002550	3.36E-12	C	T	0.521	0.009
rs62137126	2	-0.023691	0.003905	1.31E-09	G	A	0.121	0.006
rs1004787	2	0.028414	0.002562	1.11E-28	A	G	0.552	0.000
rs7598402	2	-0.014728	0.002548	7.38E-09	G	C	0.492	0.000
rs10490159	2	0.017237	0.002607	3.86E-11	T	C	0.394	0.000
rs1518393	2	0.016860	0.002624	1.30E-10	C	A	0.619	0.005
rs17616642	2	-0.016557	0.002955	2.10E-08	G	A	0.247	0.000
rs6730325	2	-0.014636	0.002612	2.10E-08	A	G	0.610	0.001
rs2539706	2	0.016245	0.002553	1.95E-10	A	G	0.530	0.006
rs7585579	2	0.020396	0.002609	5.48E-15	G	C	0.499	0.045
rs1863161	2	0.015339	0.002567	2.34E-09	A	G	0.561	0.030
rs359247	2	0.022030	0.002652	9.89E-17	T	A	0.639	0.000
rs62180324	2	-0.019517	0.003117	3.91E-10	A	G	0.212	0.003
rs6750107	2	0.014565	0.002616	2.60E-08	A	G	0.387	0.000
rs12714017	2	0.015396	0.002610	3.65E-09	C	T	0.511	0.000
rs56208390	2	0.021564	0.003879	2.68E-08	G	A	0.123	0.003
rs11692435	2	0.025053	0.004582	4.47E-08	A	G	0.085	0.003
rs13392222	2	-0.023438	0.003683	1.93E-10	C	A	0.139	0.001

rs1901477	2	0.030437	0.002610	2.07E-31	G	A	0.511	0.000
rs11889814	2	-0.021027	0.003814	3.44E-08	C	A	0.128	0.073
rs3811038	2	0.019140	0.002841	1.58E-11	C	T	0.279	0.000
rs75210106	2	-0.018659	0.003341	2.33E-08	T	C	0.177	0.071
rs34399632	2	0.019350	0.003018	1.46E-10	G	A	0.232	0.002
rs74697736	2	0.022296	0.002816	2.43E-15	A	G	0.287	0.002
**rs6756212**	2	-0.033888	0.002554	3.49E-40	T	C	0.535	0.019
rs16826827	2	-0.022207	0.003866	9.17E-09	C	T	0.124	0.002
rs1445649	2	0.020572	0.002556	8.48E-16	C	T	0.538	0.020
rs1722666	2	0.016093	0.002877	2.17E-08	T	C	0.732	0.001
rs11678980	2	0.017669	0.002561	5.19E-12	A	G	0.450	0.010
rs12474587	2	0.024231	0.002574	4.83E-21	T	G	0.429	0.030
rs357304	2	0.016676	0.002860	5.40E-09	C	T	0.727	0.003
rs13007361	2	0.017534	0.003139	2.29E-08	A	G	0.208	0.003
rs7600835	2	-0.015122	0.002686	1.80E-08	A	G	0.342	0.001
rs6750529	2	0.019907	0.002919	9.26E-12	T	C	0.744	0.008
rs17229285	2	-0.015480	0.002548	1.27E-09	T	C	0.505	0.004
rs3115418	2	-0.014225	0.002559	2.79E-08	C	T	0.454	0.001
rs62193862	2	0.023846	0.004249	1.99E-08	A	G	0.100	0.007
rs4674916	2	-0.018026	0.002714	3.06E-11	A	C	0.328	0.002
rs4674993	2	-0.024005	0.003185	4.85E-14	G	A	0.200	0.002
rs11713899	3	0.018719	0.003384	3.15E-08	C	A	0.171	0.001
rs748832	3	0.017214	0.002637	6.60E-11	G	A	0.371	0.001
rs10446419	3	-0.019563	0.003145	5.05E-10	G	A	0.207	0.006
rs13319205	3	0.016540	0.002808	3.77E-09	A	T	0.290	0.007
rs3172494	3	-0.029127	0.004001	3.40E-13	T	G	0.115	0.035
rs2526390	3	0.020466	0.002701	3.62E-14	T	C	0.334	0.021
rs2276825	3	0.018876	0.002962	1.89E-10	C	T	0.245	0.008

rs2306866	3	-0.016675	0.002617	1.89E-10	T	A	0.614	0.004
rs73831818	3	0.032043	0.005495	5.46E-09	G	A	0.057	0.005
rs1910236	3	0.014644	0.002553	9.91E-09	A	G	0.469	0.000
rs7640107	3	-0.014186	0.002573	3.46E-08	T	C	0.431	0.000
rs2734390	3	0.014771	0.002636	2.09E-08	G	A	0.372	0.002
rs221988	3	-0.014865	0.002620	1.43E-08	C	A	0.384	0.000
**rs11128203**	3	0.020406	0.002553	1.29E-15	A	T	0.530	0.000
rs62246017	3	-0.016169	0.002725	3.03E-09	A	G	0.323	0.000
rs4543050	3	0.022204	0.003288	1.45E-11	T	A	0.816	0.001
rs6782116	3	-0.014650	0.002586	1.46E-08	T	C	0.415	0.001
rs13066050	3	0.018834	0.003139	1.93E-09	T	C	0.208	0.000
rs12633090	3	-0.023020	0.003302	3.16E-12	C	G	0.182	0.000
**rs1549979**	3	-0.024522	0.002623	8.80E-21	T	C	0.615	0.014
rs57153235	3	-0.019380	0.002741	1.56E-12	G	T	0.318	0.053
rs6437769	3	0.014214	0.002582	3.74E-08	T	C	0.581	0.000
rs9288999	3	0.017441	0.002887	1.50E-09	A	G	0.735	0.002
rs6438436	3	0.024737	0.003288	5.33E-14	T	C	0.816	0.000
rs12053870	3	0.015616	0.002557	1.02E-09	G	T	0.542	0.018
rs9826984	3	-0.014053	0.002557	3.87E-08	A	G	0.542	0.001
rs2279829	3	-0.017377	0.003096	2.05E-08	T	C	0.216	0.003
rs2319545	3	0.023237	0.003577	8.30E-11	A	C	0.149	0.004
rs10935779	3	-0.014327	0.002586	2.95E-08	T	C	0.415	0.004
rs963354	3	0.015049	0.002748	4.21E-08	A	C	0.687	0.002
rs1714521	3	-0.016295	0.002589	3.07E-10	C	A	0.411	0.078
rs1449012	3	-0.015373	0.002555	1.77E-09	T	C	0.463	0.005
rs9850597	3	-0.018571	0.003288	1.65E-08	A	G	0.816	0.003
rs1187820	3	-0.014271	0.002567	2.69E-08	T	C	0.439	0.002
rs16828799	3	0.019769	0.003511	1.83E-08	T	G	0.156	0.008

rs9841807	3	0.016253	0.002860	1.35E-08	T	C	0.273	0.000
rs7631379	3	0.020801	0.003150	3.94E-11	C	T	0.206	0.014
rs4140932	4	-0.014045	0.002573	4.89E-08	A	T	0.431	0.000
rs12642744	4	-0.016591	0.002989	2.82E-08	T	G	0.744	0.000
rs59537158	4	0.022487	0.003107	4.62E-13	T	C	0.214	0.092
rs1389171	4	-0.017472	0.002979	4.45E-09	A	T	0.241	0.015
rs55944129	4	-0.017565	0.002880	1.06E-09	C	T	0.267	0.003
rs58400863	4	-0.020172	0.002677	4.89E-14	A	G	0.347	0.000
**rs7657022**	4	0.018291	0.002549	7.34E-13	G	A	0.489	0.000
rs112725451	4	0.026092	0.003400	1.65E-14	T	C	0.169	0.002
rs1160685	4	0.015302	0.002561	2.31E-09	G	C	0.450	0.000
rs1435479	4	0.016390	0.002815	5.68E-09	T	G	0.287	0.007
rs3934797	4	-0.021297	0.003302	1.12E-10	A	G	0.182	0.000
rs71602617	4	-0.017765	0.003170	2.10E-08	T	C	0.216	0.002
rs7696257	4	0.015331	0.002645	6.78E-09	A	G	0.366	0.000
rs13109980	4	-0.022182	0.002718	3.37E-16	A	G	0.326	0.002
rs1116690	4	0.016291	0.002912	2.16E-08	G	A	0.742	0.000
**rs13110073**	4	-0.024643	0.002606	3.24E-21	C	T	0.395	0.005
rs62340589	4	0.017413	0.003179	4.31E-08	C	G	0.201	0.010
rs12517438	5	0.015354	0.002556	1.89E-09	G	T	0.538	0.016
rs35375873	5	-0.027010	0.004072	3.29E-11	C	G	0.110	0.000
rs986714	5	-0.016031	0.002564	4.13E-10	T	A	0.445	0.004
rs71592686	5	0.020737	0.002857	3.85E-13	C	T	0.274	0.001
rs2028269	5	0.016165	0.002602	5.19E-10	A	G	0.399	0.000
rs6874731	5	0.015318	0.002549	1.83E-09	G	T	0.484	0.000
rs6452785	5	-0.026883	0.002552	4.69E-26	T	C	0.474	0.000
rs10805858	5	0.018124	0.002699	1.88E-11	T	A	0.335	0.003
rs181508347	5	0.081076	0.013033	4.95E-10	G	T	0.010	0.003

rs42417	5	0.016930	0.002757	8.27E-10	T	C	0.691	0.004
rs72780746	5	-0.025763	0.003368	2.05E-14	C	T	0.173	0.001
rs10060196	5	0.018312	0.002582	1.29E-12	A	C	0.581	0.002
rs72789626	5	-0.025643	0.003717	5.13E-12	A	T	0.136	0.016
rs17165769	5	0.015940	0.002606	9.56E-10	G	A	0.395	0.001
rs329124	5	-0.016387	0.002575	1.96E-10	G	A	0.428	0.000
rs1385108	5	0.018704	0.002987	3.84E-10	T	C	0.239	0.005
rs1173461	5	0.016609	0.002716	9.51E-10	T	C	0.327	0.003
rs11956866	5	-0.014838	0.002571	7.82E-09	G	T	0.567	0.011
rs3909281	5	0.021067	0.002555	1.62E-16	G	T	0.536	0.003
rs3843905	5	-0.015146	0.002597	5.41E-09	T	C	0.403	0.001
rs79476395	5	0.033374	0.004910	1.04E-11	G	A	0.073	0.000
rs6890961	5	-0.019311	0.002630	2.13E-13	T	C	0.624	0.010
rs4044321	5	-0.022641	0.002661	1.75E-17	G	A	0.644	0.000
rs2173019	5	0.028207	0.003338	2.98E-17	A	T	0.177	0.005
rs10042827	5	0.016717	0.002734	9.41E-10	C	T	0.681	0.001
rs359431	5	-0.014198	0.002567	3.16E-08	T	C	0.560	0.005
rs1059490	6	-0.018594	0.002648	2.16E-12	C	T	0.367	0.001
**rs1150668**	6	-0.018510	0.002587	8.54E-13	G	T	0.419	0.047
rs1632941	6	-0.015807	0.002561	6.67E-10	C	T	0.460	0.026
rs3218116	6	-0.019843	0.002919	1.05E-11	T	C	0.256	0.001
rs160631	6	-0.017263	0.002873	1.87E-09	G	T	0.731	0.001
**rs7743165**	6	0.019256	0.002548	4.15E-14	G	T	0.495	0.000
rs10945141	6	0.018142	0.002894	3.59E-10	A	G	0.263	0.011
rs17554906	6	0.014185	0.002564	3.14E-08	C	G	0.444	0.008
rs619087	6	0.014270	0.002580	3.10E-08	G	A	0.422	0.000
rs6568832	6	0.018869	0.002958	1.74E-10	A	G	0.754	0.000
rs12195240	6	0.024911	0.002822	1.08E-18	A	G	0.285	0.002

rs6936160	6	0.020107	0.002775	4.20E-13	T	C	0.698	0.002
rs12530388	6	-0.018362	0.002549	5.83E-13	C	A	0.511	0.000
rs3800227	6	0.017178	0.002912	3.64E-09	G	A	0.742	0.004
rs118202	6	-0.036748	0.003261	1.90E-29	T	G	0.812	0.002
rs73008357	6	-0.022310	0.004000	2.44E-08	C	A	0.121	0.001
rs9331343	6	-0.014135	0.002572	3.90E-08	C	T	0.568	0.001
rs10698713	6	-0.033517	0.005617	2.38E-09	A	G	0.054	0.008
rs1737329	6	0.017029	0.002912	5.08E-09	G	C	0.742	0.003
rs10272990	7	-0.020922	0.002715	1.27E-14	C	T	0.328	0.000
rs6948707	7	0.024347	0.002582	4.24E-21	G	T	0.419	0.015
**rs13237637**	7	-0.023682	0.002549	1.54E-20	C	G	0.485	0.003
**rs7809303**	7	-0.021419	0.002720	3.48E-15	A	G	0.325	0.000
rs7802996	7	-0.020885	0.003424	1.06E-09	T	C	0.166	0.000
rs1030015	7	0.014290	0.002550	2.15E-08	T	G	0.520	0.000
rs4727189	7	0.014860	0.002682	3.00E-08	C	T	0.344	0.005
rs76841737	7	-0.023149	0.004192	3.26E-08	G	C	0.103	0.000
rs11768481	7	-0.018556	0.002690	5.23E-12	A	C	0.340	0.000
rs1799068	7	0.016610	0.002626	2.59E-10	T	G	0.379	0.006
rs13437771	7	-0.027110	0.003520	1.39E-14	G	A	0.155	0.000
rs11766326	7	-0.017544	0.002610	1.79E-11	C	T	0.506	0.001
rs6968380	7	-0.023419	0.002734	1.05E-17	A	G	0.681	0.005
rs112913817	7	0.078056	0.012044	9.28E-11	G	A	0.011	0.013
rs10233018	7	0.024612	0.002549	4.77E-22	G	A	0.516	0.005
rs10953957	7	0.014406	0.002617	3.66E-08	A	G	0.386	0.005
rs77283305	7	-0.015196	0.002765	3.91E-08	A	G	0.306	0.003
rs10279261	7	-0.018873	0.002622	6.05E-13	A	G	0.618	0.002
rs1561112	7	-0.015244	0.002588	3.84E-09	C	T	0.413	0.086
rs2952251	8	0.016413	0.002997	4.24E-08	G	A	0.744	0.000

rs4326350	8	-0.017614	0.002553	5.16E-12	G	C	0.493	0.082
**rs11783093**	8	-0.047124	0.003493	2.07E-41	T	C	0.158	0.003
rs7836565	8	-0.015508	0.002831	4.36E-08	T	C	0.718	0.004
rs13261666	8	-0.019995	0.002550	4.36E-15	T	G	0.517	0.005
rs3850736	8	0.019128	0.002552	6.43E-14	G	C	0.474	0.000
rs2063976	8	-0.020181	0.002699	7.45E-14	T	C	0.665	0.005
rs6993429	8	-0.019050	0.002559	9.87E-14	A	C	0.453	0.006
rs6986430	8	-0.024338	0.003064	1.99E-15	C	T	0.222	0.005
rs9987376	8	-0.020468	0.002577	2.01E-15	G	T	0.574	0.011
rs290601	8	0.016310	0.002857	1.14E-08	T	C	0.274	0.001
rs3847244	9	0.018672	0.002553	2.60E-13	T	C	0.470	0.000
rs11791671	9	0.027850	0.005085	4.24E-08	T	C	0.067	0.007
rs7024924	9	0.018892	0.003361	1.90E-08	C	T	0.174	0.000
rs6474609	9	-0.015589	0.002587	1.71E-09	A	T	0.587	0.000
rs1931431	9	0.018233	0.002551	8.56E-13	C	G	0.478	0.042
rs7867822	9	-0.015097	0.002716	2.76E-08	G	A	0.673	0.009
rs10966092	9	-0.020486	0.002880	1.12E-12	C	T	0.267	0.002
rs10969352	9	0.014347	0.002548	1.82E-08	A	T	0.500	0.000
rs4877285	9	-0.018132	0.002706	2.10E-11	A	G	0.668	0.000
rs1930371	9	-0.017242	0.002979	7.09E-09	T	C	0.241	0.000
rs2378662	9	0.015212	0.002557	2.67E-09	A	G	0.541	0.006
rs1927901	9	-0.014175	0.002563	3.10E-08	C	T	0.553	0.003
rs4837631	9	-0.015357	0.002563	2.03E-09	T	C	0.446	0.002
rs1759433	9	0.015365	0.002550	1.69E-09	A	G	0.480	0.002
rs34553878	9	0.024671	0.004056	1.17E-09	G	A	0.111	0.001
rs7026534	9	-0.016603	0.002791	2.68E-09	G	T	0.704	0.017
rs10858334	9	0.022871	0.003760	1.18E-09	G	C	0.140	0.000
rs10905461	10	-0.016392	0.002935	2.36E-08	C	T	0.748	0.008

rs7920501	10	-0.015517	0.002554	1.25E-09	A	T	0.465	0.001
rs1291821	10	0.014493	0.002554	1.39E-08	G	A	0.534	0.002
rs11258417	10	-0.014514	0.002611	2.71E-08	T	C	0.391	0.000
rs7072776	10	-0.021975	0.002814	5.66E-15	G	A	0.712	0.002
rs2796793	10	0.014481	0.002560	1.55E-08	A	G	0.452	0.001
rs1733760	10	0.014773	0.002549	6.70E-09	C	T	0.510	0.001
rs7921378	10	-0.023314	0.002550	6.10E-20	C	G	0.482	0.007
rs7901883	10	-0.019257	0.003026	1.98E-10	A	G	0.230	0.000
rs11594623	10	0.027440	0.003008	7.45E-20	C	T	0.234	0.025
rs11191269	10	0.017643	0.003226	4.61E-08	G	C	0.193	0.038
rs28408682	10	0.016673	0.002601	1.41E-10	G	A	0.600	0.002
**rs12244388**	10	0.025815	0.002671	4.31E-22	A	G	0.350	0.024
rs34970111	10	-0.014556	0.002557	1.28E-08	T	C	0.458	0.002
rs9787523	10	-0.015627	0.002583	1.42E-09	C	T	0.418	0.008
rs11192347	10	-0.026450	0.004274	6.15E-10	A	G	0.104	0.044
rs10885480	10	-0.018677	0.002825	3.83E-11	C	T	0.284	0.000
rs4752018	10	0.018854	0.003023	4.42E-10	A	C	0.231	0.011
rs9423279	10	-0.018581	0.002663	3.06E-12	G	C	0.645	0.000
rs6265	11	-0.029275	0.003261	2.81E-19	T	C	0.188	0.003
rs4275621	11	-0.021367	0.002622	3.76E-16	G	A	0.382	0.009
rs62618693	11	-0.035272	0.006295	2.09E-08	T	C	0.043	0.000
rs2939756	11	-0.015700	0.002550	7.45E-10	A	G	0.480	0.006
rs1381775	11	-0.015615	0.002814	2.79E-08	C	T	0.712	0.006
rs2959084	11	0.017080	0.002793	9.82E-10	A	G	0.705	0.000
rs3740977	11	0.019474	0.003416	1.17E-08	C	T	0.167	0.007
rs61886926	11	-0.017940	0.002620	7.30E-12	T	C	0.384	0.013
rs61884449	11	0.019975	0.003576	2.32E-08	T	C	0.149	0.003
rs644740	11	-0.014079	0.002558	3.67E-08	T	C	0.457	0.000

rs7943721	11	-0.021214	0.003384	3.58E-10	A	G	0.829	0.003
rs7929518	11	0.019236	0.003042	2.55E-10	G	A	0.773	0.001
rs586699	11	-0.014803	0.002558	7.29E-09	A	G	0.543	0.000
rs76460663	11	-0.042350	0.006421	4.15E-11	G	C	0.041	0.000
**rs2155646**	11	0.037777	0.002601	9.44E-48	C	T	0.400	0.001
rs1713676	11	-0.016726	0.002551	5.38E-11	G	A	0.523	0.002
rs238896	11	-0.016867	0.002549	3.65E-11	A	G	0.490	0.001
rs540860	11	0.017609	0.002558	5.75E-12	G	A	0.543	0.000
rs1834306	11	-0.014485	0.002581	1.96E-08	G	A	0.579	0.002
rs1106363	11	0.017375	0.002681	9.20E-11	T	C	0.345	0.007
rs2010921	11	0.017429	0.002752	2.47E-10	A	G	0.311	0.004
rs11057005	12	-0.015714	0.002566	9.12E-10	G	A	0.441	0.005
rs13906	12	-0.024530	0.004088	1.98E-09	T	C	0.109	0.003
rs4759229	12	0.015570	0.002682	6.53E-09	G	A	0.656	0.000
rs7969559	12	-0.017016	0.002816	1.53E-09	G	A	0.713	0.000
rs7134009	12	-0.015797	0.002884	4.30E-08	C	T	0.287	0.000
rs77215829	12	-0.024045	0.003783	2.02E-10	C	A	0.131	0.008
rs1109480	12	-0.016692	0.002620	1.84E-10	A	G	0.384	0.003
rs11611651	12	0.027114	0.004525	2.05E-09	A	G	0.087	0.001
rs17197663	13	-0.021587	0.003852	2.06E-08	A	G	0.125	0.002
rs4264267	13	0.014792	0.002552	6.82E-09	T	C	0.527	0.000
rs61959481	13	-0.020344	0.003128	7.95E-11	A	G	0.210	0.000
rs3098272	13	-0.017808	0.003178	2.08E-08	C	A	0.799	0.077
rs9538162	13	0.017379	0.002585	1.76E-11	C	T	0.416	0.000
rs56367474	13	-0.017298	0.002770	4.20E-10	T	C	0.304	0.034
rs55786907	13	0.019445	0.003454	1.84E-08	G	A	0.162	0.008
rs4886207	13	-0.016247	0.002650	8.78E-10	C	T	0.637	0.000
rs9540731	13	-0.017730	0.002549	3.42E-12	T	C	0.509	0.005

rs9545155	13	-0.016071	0.002551	3.04E-10	C	T	0.478	0.005
rs1772572	13	-0.016868	0.002722	5.62E-10	A	C	0.324	0.000
rs75674569	13	-0.025338	0.004253	2.58E-09	A	G	0.100	0.001
rs7333559	13	-0.023213	0.003091	5.94E-14	A	G	0.783	0.001
rs1108130	13	0.023944	0.003117	1.57E-14	A	T	0.212	0.004
rs12855717	13	0.015524	0.002556	1.22E-09	T	C	0.538	0.001
rs12878369	14	0.017437	0.002588	1.60E-11	A	C	0.415	0.006
rs2145451	14	-0.020046	0.003231	5.44E-10	C	T	0.193	0.003
rs9323328	14	-0.014237	0.002557	2.55E-08	G	A	0.537	0.000
rs1811739	14	0.018272	0.002952	5.97E-10	A	G	0.248	0.019
rs8005334	14	0.016673	0.002656	3.44E-10	G	T	0.360	0.001
rs34940743	14	0.015925	0.002680	2.80E-09	G	A	0.346	0.006
rs2925128	14	0.016820	0.002683	3.67E-10	T	C	0.385	0.015
rs1381287	14	0.018017	0.002556	1.81E-12	T	C	0.467	0.005
rs55913542	14	0.018562	0.003356	3.25E-08	T	G	0.175	0.001
rs1435672	15	0.014105	0.002567	3.82E-08	C	T	0.560	0.001
**rs281296**	15	0.024689	0.002659	1.59E-20	A	G	0.357	0.000
rs56902655	15	-0.021863	0.003717	4.09E-09	G	T	0.136	0.004
rs2289791	15	-0.017726	0.002954	2.01E-09	T	G	0.247	0.007
rs60833441	15	-0.014277	0.002556	2.28E-08	G	A	0.461	0.001
rs62007780	15	-0.015913	0.002585	7.48E-10	T	G	0.416	0.000
rs12442563	15	-0.023230	0.003061	3.13E-14	T	G	0.223	0.001
rs4310804	15	-0.018187	0.002954	7.55E-10	G	C	0.247	0.000
rs8027457	15	0.015314	0.002549	1.88E-09	C	T	0.511	0.000
rs1139897	16	-0.024087	0.003028	1.77E-15	A	G	0.230	0.003
rs11076962	16	0.018300	0.002841	1.20E-10	C	T	0.279	0.003
rs7192140	16	-0.016883	0.002548	3.40E-11	C	T	0.498	0.004
rs9922607	16	-0.022159	0.003185	3.42E-12	T	C	0.200	0.013

rs9941217	16	-0.018557	0.002667	3.50E-12	G	C	0.352	0.000
rs7188873	16	0.020296	0.002616	8.46E-15	G	A	0.613	0.001
rs6497840	16	0.022770	0.002867	2.01E-15	A	G	0.707	0.005
rs4785187	16	0.019977	0.003061	6.55E-11	A	G	0.223	0.000
rs8050598	16	0.018670	0.002926	1.76E-10	T	C	0.254	0.001
rs12918191	16	-0.019727	0.002971	3.14E-11	G	A	0.243	0.005
rs9302604	16	0.018710	0.002570	3.29E-13	G	A	0.435	0.000
rs9936784	16	0.013989	0.002554	4.33E-08	G	T	0.534	0.001
rs62052916	16	-0.031914	0.004990	1.62E-10	T	A	0.070	0.051
rs4788676	16	-0.017745	0.003034	4.92E-09	C	T	0.229	0.001
**rs117657830**	16	-0.037760	0.006373	3.18E-09	G	A	0.042	0.006
rs1050847	16	-0.014830	0.002566	7.37E-09	T	C	0.559	0.001
rs11642231	16	-0.015598	0.002640	3.44E-09	A	G	0.369	0.005
rs4790874	17	0.017449	0.002553	8.43E-12	T	C	0.532	0.006
rs11078713	17	-0.014583	0.002582	1.59E-08	G	A	0.419	0.000
rs28441558	17	-0.035565	0.005527	1.24E-10	C	T	0.056	0.082
rs11651955	17	-0.014026	0.002548	3.74E-08	A	G	0.499	0.003
rs67777803	17	-0.024601	0.003376	3.18E-13	T	G	0.172	0.000
rs2344976	17	-0.015088	0.002615	7.98E-09	C	T	0.612	0.001
rs3764351	17	-0.014749	0.002684	3.89E-08	A	G	0.657	0.000
**rs17692129**	17	0.019599	0.002707	4.57E-13	T	C	0.331	0.005
rs75919030	17	-0.020966	0.002880	3.35E-13	C	T	0.267	0.000
rs2938134	17	-0.017501	0.002781	3.14E-10	A	C	0.673	0.086
rs2587507	17	-0.014660	0.002548	8.69E-09	C	T	0.502	0.003
rs34342129	18	-0.014281	0.002549	2.13E-08	C	T	0.509	0.003
rs4476253	18	-0.018486	0.002983	5.78E-10	A	G	0.240	0.000
rs7505855	18	-0.016982	0.002587	5.31E-11	T	C	0.586	0.002
rs8096225	18	0.015525	0.002788	2.63E-08	C	A	0.703	0.005

rs67050670	18	-0.020272	0.003032	2.34E-11	G	A	0.229	0.009
rs2359180	18	-0.014389	0.002640	4.98E-08	G	A	0.369	0.000
rs72898831	18	-0.024416	0.003520	4.14E-12	G	A	0.155	0.001
rs8083764	18	-0.015952	0.002764	7.97E-09	T	G	0.306	0.000
rs1373178	18	-0.020316	0.002589	4.16E-15	G	T	0.588	0.059
rs62098013	18	0.017710	0.002646	2.24E-11	A	G	0.365	0.006
rs72938304	18	-0.027205	0.004024	1.36E-11	A	G	0.113	0.007
rs11872397	18	-0.017114	0.002931	5.20E-09	A	G	0.253	0.000
rs71367544	18	0.020552	0.003167	8.54E-11	T	C	0.203	0.016
rs76608582	19	-0.034549	0.005908	4.88E-09	A	C	0.049	0.009
rs10853981	19	0.014787	0.002709	4.88E-08	A	G	0.330	0.000
rs113230003	19	-0.018876	0.002923	1.05E-10	A	G	0.255	0.002
rs8103660	19	0.015803	0.002664	3.03E-09	C	T	0.354	0.000
rs117734003	19	0.030299	0.005085	2.57E-09	C	G	0.067	0.003
rs1126757	19	0.014162	0.002552	2.92E-08	T	C	0.473	0.003
rs6050446	20	0.054410	0.007611	8.80E-13	G	A	0.971	0.000
rs1555445	20	0.018763	0.002743	7.75E-12	T	A	0.318	0.012
rs6073075	20	-0.018704	0.003354	2.44E-08	A	T	0.824	0.000
rs910912	20	-0.016770	0.002906	7.82E-09	C	T	0.739	0.000
rs6011779	20	-0.019177	0.003228	2.83E-09	T	C	0.806	0.021
rs3810496	20	0.015881	0.002629	1.54E-09	C	T	0.619	0.012
rs4818005	21	-0.020431	0.002644	1.09E-14	A	G	0.581	0.000
rs139896	22	0.015440	0.002668	7.14E-09	C	T	0.648	0.000
rs4822102	22	-0.016543	0.002622	2.78E-10	T	C	0.618	0.000
rs9627272	22	-0.015474	0.002593	2.42E-09	C	G	0.407	0.000

Note. Excluded Linkage disequilibrium SNPs: rs80054503 (r^2 =0.756 with rs12740789), rs3076896 (r^2 =0.219 with rs6756212), rs2196356 (r^2 =0.128 with rs11128203), rs74664784 (r^2 =0.977 with rs1549979), rs55900829 (r^2 =0.989 with rs7657022), rs28717373 (r^2 =0.163 with rs13110073), rs6932350 (r^2 =0.150 with rs1150668), rs79180767 (r^2 =0.617 with rs7743165), rs10259715 (r^2 =0.474 with rs13237637), rs79631993 (r^2 =0.949 with rs7809303), rs11780471 (r^2 =0.288 with rs11783093), rs1565735 (r^2 =0.727 with rs11783093), rs111842178 (r^2 =0.777 with rs12244388), rs78239456 (r^2 =0.329 with rs2155646), rs1944689 (r^2 =0.057 with rs540860), rs1435741 (r^2 = 0.241 with rs281296), rs61537885 (r^2 =0.820 with rs117657830), rs72836318 (r^2 =0.170 with rs17692129).

Table 4.3. Characteristics of genetic instruments associated with current smoking per day

SNP	Chromosome	beta	se	p-value	effect_allele	other_allele	eaf	R^2
rs11264100	1	-0.022166	0.003705	2.22E-09	G	A	0.876	0.001
rs2072659	1	-0.029984	0.004096	2.51E-13	G	C	0.099	0.000
rs34973462	1	0.015073	0.002589	5.85E-09	T	C	0.334	0.000
rs7599488	2	0.014121	0.002454	8.95E-09	T	C	0.437	0.003
rs78408772	2	-0.021996	0.004023	4.51E-08	T	C	0.102	0.001
rs10204824	2	-0.017978	0.002535	1.35E-12	G	A	0.639	0.005
rs2084533	3	0.016118	0.002608	6.53E-10	T	C	0.321	0.016
rs7431710	3	-0.018290	0.002566	1.04E-12	A	G	0.654	0.006
rs2236951	3	-0.017191	0.003044	1.59E-08	C	T	0.200	0.040
rs699165	3	0.016119	0.002793	8.09E-09	G	A	0.745	0.001
rs28813180	3	-0.015496	0.002435	1.95E-10	A	G	0.498	0.000
rs1024323	4	-0.014416	0.002506	8.66E-09	T	C	0.382	0.000
rs11940255	4	-0.017158	0.002703	2.2E-10	A	G	0.717	0.001
rs10454798	4	0.015842	0.002800	1.53E-08	T	G	0.253	0.003
rs7766641	6	-0.017284	0.002743	2.91E-10	A	G	0.272	0.000
rs215600	7	-0.024002	0.002544	4.02E-21	A	G	0.645	0.001
rs62447179	7	-0.015268	0.002662	9.68E-09	A	G	0.298	
rs2741351	8	0.018476	0.003211	8.8E-09	C	A	0.826	0.005
rs73229090	8	0.026211	0.003860	1.14E-11	A	C	0.112	0.016
rs13253502	8	-0.013842	0.002478	2.31E-08	A	G	0.407	0.002
rs4236926	8	0.034267	0.002876	7.66E-33	G	T	0.766	0.024
rs790564	8	-0.017624	0.002739	1.24E-10	C	A	0.729	0.025
rs75596189	9	0.035800	0.003860	1.84E-20	T	C	0.112	0.011
rs3025383	9	-0.031380	0.003122	9.78E-24	C	T	0.187	
rs7951365	11	0.017756	0.002632	1.53E-11	C	T	0.310	0.003

rs10742683	11	-0.013488	0.002471	4.83E-08	A	G	0.415	0.002
rs113001570	11	0.029801	0.004880	1.04E-09	T	A	0.067	0.001
rs7125588	11	-0.016900	0.002460	6.5E-12	G	A	0.429	0.000
rs11846838	14	0.015177	0.002595	5.03E-09	A	G	0.327	0.000
rs1115019	15	-0.017859	0.002989	2.27E-09	C	T	0.790	0.000
rs632811	15	-0.017751	0.002779	1.67E-10	G	A	0.330	0.003
rs4886550	15	-0.019931	0.003398	4.58E-09	G	A	0.288	0.001
**rs10519203**	15	-0.093618	0.002587	3.12E-286	A	G	0.655	0.000
rs182317	15	-0.015588	0.002570	1.31E-09	T	G	0.355	0.008
rs1592485	16	-0.016153	0.002504	1.11E-10	A	C	0.611	0.003
rs12924872	16	-0.013412	0.002449	4.39E-08	T	C	0.463	0.000
rs258321	16	0.015796	0.002467	1.53E-10	G	A	0.429	0.000
rs4144686	18	-0.018551	0.003264	1.35E-08	A	G	0.167	0.000
rs4485470	18	-0.015271	0.002477	7.05E-10	A	G	0.592	
rs59208569	19	0.020476	0.003234	2.45E-10	C	G	0.829	0.001
rs143200968	19	-0.086105	0.007860	6.97E-28	C	G	0.025	0.005
rs56113850	19	0.052310	0.002474	4.01E-99	C	T	0.555	0.026
rs8192726	19	-0.039344	0.004888	8.35E-16	A	C	0.068	0.079
rs117824460	19	-0.095260	0.007727	7.66E-35	G	A	0.026	0.002
rs6078373	20	0.016073	0.002483	9.4E-11	A	G	0.402	0.001
rs1737894	20	0.016856	0.002477	9.9E-12	G	C	0.408	0.003
rs2273500	20	0.036386	0.003438	3.49E-26	C	T	0.147	0.001
rs7281463	21	0.013678	0.002473	3.15E-08	C	A	0.413	0.000

Note. Excluded Linkage disequilibrium SNPs: rs28438420, rs72740955, rs146009840, rs28681284, rs8040868, rs3743063 (r^2 =0.396, 0.977, 0.898, 0.124, 0.704, and 0.339, respectively.) in a block with rs10519203.

Table 4.4. Characteristics of genetic instruments associated with smoking cessation

SNP	Chromosome	beta	se	p-value	effect_allele	other_allele	eaf	R^2
rs112187834	2	0.033410	0.005623	2.81E-09	A	T	0.140	0.000
rs7617480	3	-0.032871	0.004658	1.68E-12	C	A	0.773	0.000
rs12203592	6	-0.029209	0.005124	1.21E-08	T	C	0.176	0.009
rs707968	6	0.023271	0.004186	2.76E-08	G	A	0.681	
rs7778443	7	-0.022999	0.004016	1.04E-08	C	T	0.618	0.000
rs1565735	8	-0.034557	0.004887	1.54E-12	A	T	0.199	0.000
rs60749569	8	-0.040071	0.007209	2.68E-08	T	A	0.080	
rs12378015	9	-0.027660	0.004258	8.31E-11	A	G	0.300	0.000
rs9409844	9	-0.058561	0.009389	4.37E-10	A	G	0.045	0.000
rs3025327	9	0.078586	0.006312	1.19E-35	C	G	0.107	0.007
rs10821523	9	0.026157	0.003912	2.28E-11	C	A	0.536	0.071
rs1611124	9	-0.045338	0.007764	5.26E-09	T	G	0.068	0.008
rs7109376	11	0.028059	0.004350	1.14E-10	A	T	0.279	0.000
rs591143	15	-0.024330	0.003994	1.14E-09	T	C	0.592	0.003
rs3866543	15	0.022202	0.003907	1.35E-08	G	T	0.523	0.005
rs518425	15	-0.030503	0.004322	1.72E-12	G	A	0.285	0.002
rs145580088	19	0.090955	0.012749	9.48E-13	G	A	0.024	0.001
rs56113850	19	-0.057606	0.003938	1.61E-48	C	T	0.567	0.026
rs117824460	19	0.086499	0.012148	1.09E-12	G	A	0.027	0.052
rs59586387	19	0.051388	0.007756	3.37E-11	G	C	0.068	0.002
**rs6011779**	20	-0.050022	0.004977	9.89E-24	T	C	0.806	0.011
rs6089904	20	-0.064183	0.009254	4.01E-12	T	A	0.047	0.004
rs9607805	22	0.029541	0.004370	1.37E-11	T	C	0.725	0.000

Note. Excluded Linkage disequilibrium SNPs: rs4809543 (r^2 =0.358 with rs6011779).

Table 4.5. Characteristics of genetic instruments associated with common alcohol use per week

SNP	Chromosome	beta	se	p-value	effect_allele	other_allele	eaf	R^2
rs705687	1	-0.010904	0.001776	8.15E-10	G	A	0.785	0.001
rs58107686	1	-0.009747	0.001585	7.79E-10	A	C	0.328	0.004
rs12088813	1	-0.009329	0.001649	1.58E-08	C	A	0.267	0.004
rs5024204	1	0.009703	0.001628	2.55E-09	T	A	0.278	0.001
rs10753661	1	-0.008638	0.001569	3.76E-08	A	G	0.684	0.000
rs28680958	1	-0.010996	0.001770	5.13E-10	A	G	0.217	0.000
rs823114	1	0.008768	0.001467	2.31E-09	A	G	0.553	0.001
rs77165542	2	-0.026011	0.003971	5.63E-11	T	C	0.035	0.000
rs1260326	2	0.020890	0.001488	8.05E-45	C	T	0.601	0.016
rs2178197	2	-0.008781	0.001472	2.45E-09	G	A	0.569	0.065
rs13383034	2	0.014927	0.001551	6.31E-22	T	C	0.329	0.000
rs13032049	2	0.010195	0.001618	3E-10	G	A	0.283	0.003
rs828867	2	0.008757	0.001464	2.15E-09	A	G	0.545	0.003
rs11692435	2	0.017450	0.002616	2.53E-11	A	G	0.085	0.009
rs13024996	2	-0.010913	0.001515	5.72E-13	A	C	0.364	0.005
rs72859280	2	0.022885	0.003902	4.44E-09	T	G	0.036	0.007
rs56337305	2	-0.009588	0.001499	1.63E-10	C	T	0.383	0.001
rs13094887	3	-0.010310	0.001589	8.57E-11	T	A	0.301	0.000
rs62250685	3	-0.014357	0.001500	1.05E-21	G	A	0.614	0.001
rs13066454	3	-0.008776	0.001492	4.13E-09	T	C	0.398	0.001
rs9838144	3	-0.009964	0.001793	2.65E-08	C	G	0.209	0.000
rs2011092	3	-0.008900	0.001540	7.35E-09	C	T	0.339	0.004
rs60654199	3	-0.016660	0.003002	2.85E-08	A	C	0.063	0.020
rs6787172	3	-0.008031	0.001466	4.27E-08	G	T	0.554	0.019
rs3748034	4	-0.011740	0.002082	1.67E-08	T	G	0.143	0.001

rs7682824	4	0.008356	0.001503	2.77E-08	T	C	0.548	0.003
rs11940694	4	0.025950	0.001486	3.03E-68	G	A	0.597	0.091
rs4501255	4	0.010693	0.001719	4.83E-10	G	C	0.235	0.003
rs12499107	4	0.012661	0.002159	4.45E-09	G	A	0.131	0.000
rs144198753	4	-0.041827	0.005903	1.35E-12	T	C	0.016	0.000
rs1154414	4	0.017630	0.002094	3.74E-17	C	T	0.141	0.000
rs1229984	4	0.150534	0.003861	<2.2e-308	C	T	0.963	0.001
rs561222871	4	-0.038807	0.003619	6.56E-27	T	C	0.047	0.007
rs36052336	4	-0.018429	0.003034	1.23E-09	G	A	0.061	0.004
rs2165670	4	0.023080	0.002364	1.67E-22	A	G	0.106	0.008
rs79139602	4	0.060272	0.005076	1.8E-32	T	A	0.021	0.000
rs4699791	4	0.018571	0.002477	6.58E-14	A	G	0.096	0.001
rs13107325	4	-0.027505	0.002816	1.53E-22	T	C	0.072	0.002
rs4690727	4	0.010817	0.001620	2.43E-11	G	C	0.718	0.000
rs10004020	4	0.009052	0.001623	2.43E-08	A	G	0.720	0.000
rs12651313	4	-0.008643	0.001467	3.79E-09	G	C	0.443	0.003
rs4916723	5	-0.009952	0.001479	1.72E-11	C	A	0.416	0.014
rs12655091	5	-0.008312	0.001460	1.25E-08	A	G	0.530	0.015
rs55872084	5	0.009979	0.001719	6.32E-09	T	G	0.235	0.000
rs11739827	5	-0.008375	0.001469	1.18E-08	T	G	0.451	0.000
rs10085696	7	-0.011409	0.001873	1.12E-09	G	A	0.186	0.007
rs6460047	7	0.011624	0.001796	9.69E-11	C	T	0.208	0.000
rs10236149	7	-0.013498	0.002219	1.18E-09	G	A	0.123	0.000
rs35034355	7	-0.008097	0.001459	2.87E-08	A	G	0.521	0.006
rs6951574	7	0.013222	0.001463	1.58E-19	C	T	0.458	0.000
rs13250583	8	-0.009718	0.001780	4.7E-08	T	C	0.213	0.005
rs1217091	8	0.012161	0.001865	7.05E-11	C	T	0.812	0.001
rs28601761	8	0.009103	0.001477	7.17E-10	G	C	0.420	0.006

rs55932213	9	0.009488	0.001654	9.55E-09	G	A	0.736	0.001
rs10978550	9	-0.011748	0.001802	7.15E-11	C	T	0.206	
rs7074871	10	-0.009400	0.001672	1.86E-08	A	G	0.255	0.004
rs17665139	10	-0.011560	0.002047	1.59E-08	T	C	0.149	
rs7950166	11	-0.009799	0.001516	9.89E-11	T	C	0.637	0.000
rs11030084	11	-0.010607	0.001881	1.72E-08	T	C	0.184	0.006
rs56030824	11	-0.011602	0.001563	1.15E-13	A	G	0.322	0.006
rs10750025	11	0.010321	0.001570	4.89E-11	T	C	0.686	0.001
rs1713676	11	-0.007992	0.001459	4.29E-08	G	A	0.522	0.003
rs4938230	11	0.012810	0.001998	1.48E-10	A	C	0.842	0.001
rs682011	11	0.008212	0.001468	2.22E-08	C	T	0.559	0.002
rs12795042	11	-0.008319	0.001504	3.25E-08	C	A	0.623	0.002
rs10876188	12	-0.007987	0.001463	4.84E-08	T	C	0.457	0.003
rs3809162	12	0.009061	0.001490	1.19E-09	G	A	0.397	0.001
rs10506274	12	-0.009037	0.001458	5.78E-10	T	G	0.484	0.007
rs4842786	12	-0.008798	0.001479	2.73E-09	A	G	0.584	0.001
rs500321	13	-0.009669	0.001653	4.92E-09	T	A	0.736	0.000
rs1123285	14	-0.008897	0.001544	8.14E-09	G	C	0.335	0.001
rs2180870	14	-0.012178	0.002133	1.12E-08	C	T	0.135	0.001
rs28929474	14	-0.036800	0.005438	1.34E-11	T	C	0.018	0.004
rs11625650	14	-0.009568	0.001724	2.89E-08	A	G	0.233	0.002
rs2472297	15	0.010606	0.001685	3.1E-10	T	C	0.249	0.001
rs12907323	15	0.008497	0.001481	9.93E-09	G	A	0.411	
rs2764771	16	0.009891	0.001582	4.02E-10	A	G	0.307	0.000
rs17177078	16	-0.022315	0.003012	1.27E-13	T	C	0.063	0.003
rs378421	16	-0.011205	0.001487	4.83E-14	A	G	0.404	0.000
rs113443718	16	-0.010208	0.001585	1.19E-10	A	G	0.305	0.006
rs62044525	16	-0.012173	0.001883	1.03E-10	G	C	0.184	0.010

rs7185555	16	-0.011101	0.002027	4.24E-08	C	G	0.153	0.003
rs79616692	16	0.016302	0.002351	4.11E-12	C	G	0.108	0.002
rs1104608	16	-0.010975	0.001476	1.05E-13	C	G	0.425	0.000
rs4548913	17	-0.008360	0.001511	3.11E-08	A	G	0.632	0.000
rs3803800	17	0.011379	0.001777	1.5E-10	G	A	0.786	0.001
rs2854334	17	0.009221	0.001498	7.51E-10	G	A	0.615	0.007
rs2532276	17	-0.021800	0.002559	1.62E-17	A	C	0.215	0.001
rs10438820	17	0.008972	0.001593	1.76E-08	T	C	0.702	0.001
rs9950000	18	-0.009118	0.001491	9.38E-10	T	C	0.395	0.001
rs4092465	18	-0.008292	0.001514	4.39E-08	G	A	0.635	
rs281379	19	0.013722	0.001458	4.91E-21	A	G	0.508	0.000
rs4815364	20	0.008582	0.001499	1.02E-08	A	G	0.616	0.000
rs9607814	22	-0.010181	0.001859	4.31E-08	A	C	0.200	0.000

Note. Excluded Linkage disequilibrium SNPs: rs1004787 (r^2 =0.438 with rs13383034), rs74664784 (r^2 =0.977 with rs62250685), rs35538052 (r^2 =0.923 with rs11940694), rs10028756 (r^2 =0.256 with rs1154414), rs17029090 (r^2 =1.0 with rs79139602).

Table 4.6. Characteristics of genetic instruments associated with problematic alcohol use

SNP	Chromosome	beta	se	p-value	effect_allele	other_allele	eaf	R^2
rs6421482	1	-0.016437	0.002603	2.70E-10	A	G	0.436	0.001
rs61767420	1	0.015317	0.002681	1.11E-08	A	G	0.400	
rs1260326	2	-0.024563	0.002642	1.45E-20	T	C	0.403	0.001
rs494904	2	-0.021371	0.002696	2.26E-15	T	C	0.596	0.000
rs1402398	2	0.019088	0.002689	1.27E-12	A	G	0.627	0.001
rs9679319	2	-0.015647	0.002604	1.86E-09	T	G	0.480	0.005
rs13382553	2	-0.018415	0.003069	1.97E-09	A	G	0.766	0.000
rs2673136	2	-0.015802	0.002691	4.31E-09	A	G	0.639	0.001
rs62250713	3	0.016267	0.002689	1.46E-09	A	G	0.368	0.000
rs13129401	4	-0.023316	0.002618	5.29E-19	A	G	0.453	0.004
rs2602856	4	-0.015678	0.002732	9.56E-09	A	C	0.661	0.000
rs1154431	4	0.088948	0.011167	1.65E-15	A	G	0.975	0.009
rs13125415	4	-0.024219	0.002669	1.16E-19	A	G	0.585	0.031
rs12505678	4	0.027322	0.004001	8.64E-12	T	C	0.880	0.026
rs10553521	4	0.015108	0.002668	1.49E-08	A	AAGAT	0.398	0.060
rs138764179	4	0.016396	0.003001	4.68E-08	T	C	0.745	0.018
rs13135092	4	0.056302	0.004823	1.75E-31	A	G	0.919	0.001
rs2533200	7	-0.015305	0.002718	1.79E-08	C	G	0.516	0.000
rs2582405	8	0.017499	0.003043	8.86E-09	T	C	0.237	0.000
rs7900002	10	-0.014549	0.002644	3.74E-08	T	G	0.601	0.001
rs56722963	10	-0.019028	0.002985	1.85E-10	T	C	0.255	0.000
rs34806762	10	0.021776	0.003891	2.19E-08	T	G	0.128	0.015
rs10717830	11	0.017977	0.002799	1.34E-10	G	GT	0.674	0.000
rs576859	11	0.020327	0.003585	1.43E-08	A	C	0.327	0.015
rs138084129	11	0.021129	0.002701	5.13E-15	A	AATAT	0.627	0.001
rs6589386	11	-0.019549	0.002603	5.88E-14	T	C	0.432	0.052

rs1783835	11	-0.015579	0.002606	2.24E-09	A	G	0.457	0.014
rs10790456	11	0.023175	0.004123	1.89E-08	A	G	0.780	0.081
rs12296477	12	0.014307	0.002609	4.15E-08	C	G	0.547	0.000
rs61974485	14	0.016631	0.003020	3.67E-08	T	C	0.265	0.001
rs8008020	14	0.015959	0.002633	1.35E-09	T	C	0.418	
rs72768626	16	0.032190	0.005757	2.26E-08	A	G	0.945	0.003
rs9937709	16	0.017382	0.002633	4.06E-11	A	G	0.585	
rs492602	19	-0.015998	0.002604	8.08E-10	A	G	0.508	0.000

Table 4.7. Characteristics of genetic instruments associated with daily caffeine intake

SNP	Chromosome	beta	se	p-value	effect_allele	other_allele	eaf	R^2
rs77476394	1	-0.016	0.003	1.60E-08	CTAAA	C	0.209	0.002
rs4615895	1	-0.017	0.003	8.60E-11	G	A	0.260	0.003
rs11204711	1	-0.014	0.002	6.40E-10	A	G	0.616	0.002
rs56188862	1	0.017	0.002	2.40E-13	T	C	0.615	0.000
rs768283768	1	0.025	0.002	1.20E-23	A	AC	0.420	NA
rs7571970	2	-0.021	0.003	9.40E-12	T	C	0.173	0.001
**rs1260326**	2	-0.023	0.002	4.20E-22	T	C	0.392	0.001
**rs35198275**	3	0.021	0.003	8.20E-10	A	G	0.866	0.000
rs2117137	3	-0.013	0.002	2.90E-09	A	G	0.595	0.002
rs78456557	3	-0.024	0.004	1.20E-09	C	G	0.900	0.006
rs115454798	3	-0.023	0.003	1.10E-10	A	G	0.872	0.002
**rs2231142**	4	0.039	0.004	1.10E-26	G	T	0.887	0.000
**rs62332762**	4	0.018	0.002	1.20E-14	C	T	0.598	0.000
rs12514566	5	0.017	0.002	2.20E-12	G	A	0.665	0.001
rs6893807	5	-0.019	0.003	1.30E-09	A	G	0.844	0.004
rs192084998	5	0.016	0.002	1.20E-10	G	A	0.704	0.000
**rs2465018**	6	-0.022	0.003	1.10E-16	G	A	0.769	0.000
rs1490384	6	-0.016	0.002	8.30E-12	C	T	0.501	0.002
rs139797380	6	0.107	0.014	3.70E-15	C	G	0.992	0.000
rs754177720	6	-0.014	0.002	4.40E-09	CA	C	0.480	NA
rs4410790	7	-0.081	0.002	1.50E-249	T	C	0.364	0.005
rs215601	7	0.015	0.002	6.00E-10	A	C	0.373	0.003
rs141180025	7	-0.014	0.002	2.10E-09	CT	C	0.391	0.000
rs34060476	7	-0.025	0.003	7.10E-14	A	G	0.866	0.008
**rs17685**	7	-0.041	0.003	3.80E-56	G	A	0.721	0.000
rs4240624	8	0.027	0.004	1.90E-11	G	A	0.092	0.000
rs62534435	9	-0.016	0.003	4.40E-09	C	G	0.797	0.000
rs12785227	10	0.015	0.003	6.50E-09	A	G	0.686	0.006

rs4418728	10	0.014	0.002	7.00E-10	G	T	0.551	0.000
rs76881016	10	-0.026	0.004	6.60E-09	A	G	0.928	0.000
rs117810762	10	-0.068	0.009	1.30E-14	G	A	0.982	0.001
rs11022752	11	-0.015	0.003	1.20E-09	A	G	0.731	0.000
rs10741694	11	-0.015	0.002	1.80E-10	T	C	0.373	0.001
rs6265	11	0.022	0.003	3.40E-13	C	T	0.811	0.001
rs1228024	11	0.014	0.002	1.30E-08	C	A	0.340	0.001
**rs2298527**	11	0.015	0.002	1.60E-10	G	C	0.406	0.000
rs376877108	12	0.018	0.003	9.10E-10	GTT	G	0.201	NA
rs73053413	12	0.022	0.003	7.10E-13	C	T	0.837	
rs12591786	15	0.020	0.003	3.00E-10	C	T	0.842	0.007
rs2472297	15	-0.105	0.003	2.40E-351	C	T	0.732	0.000
rs2667773	15	0.015	0.003	2.50E-09	A	G	0.687	0.012
rs2521501	15	0.015	0.002	1.60E-09	A	T	0.677	0.000
**rs201399553**	16	-0.023	0.002	2.60E-21	T	TTC	0.594	0.001
rs199602679	16	-0.016	0.003	1.00E-08	G	GT	0.784	0.012
rs139937261	17	-0.015	0.002	9.80E-11	C	CG	0.488	0.000
rs531431865	17	-0.019	0.003	2.70E-13	C	CA	0.679	
**rs66723169**	18	-0.022	0.003	1.80E-16	C	A	0.769	0.521
rs61141867	19	-0.018	0.003	1.40E-11	T	TG	0.254	0.001
rs56113850	19	-0.026	0.002	1.90E-29	T	C	0.422	
rs6063085	20	-0.016	0.002	3.40E-11	A	C	0.625	0.001
**rs140775622**	20	-0.023	0.003	1.80E-12	C	T	0.831	0.000
rs4817505	21	-0.015	0.002	4.20E-11	T	C	0.608	0.000
rs9624470	22	-0.025	0.002	6.80E-29	G	A	0.419	0.001
**rs199612805**	22	0.096	0.010	3.10E-21	T	TGAAACCA	0.986	0.012
**rs9611527**	22	0.020	0.002	1.00E-15	G	A	0.664	0.000

Note. Excluded Linkage disequilibrium SNPs: rs11127048 (r^2 =0.782 with rs1260326), rs145755097 (r^2 =0.115 with rs35198275), rs1481012 (r^2 =0.977 with rs2231142), rs2726513 (r^2 =0.937 with rs62332762), rs1327259 (r^2 =0.458 with rs2465018), rs1057868 (r^2 =0.949 with rs17685), rs7105462 (r^2 =0.978 with rs2298527), rs28429148 (r^2 =0.682 with rs201399553), rs489693 (r^2 =0.521 with rs66723169), rs6062679 (r^2 =0.221 with rs140775622), rs181251778 (r^2 =1.000 with rs199612805), rs132919 (r^2 =0.418 with rs9611527). NA means it can't calculate.

Table 4.8. Characteristics of genetic instruments associated with coffee consumption cups per day

SNP	Chromosome	beta	se	p-value	effect_allele	other_allele	eaf	R^2
rs4615895	1	-0.017050	0.002627	8.60E-11	G	A	0.260	0.000
rs768283768	1	0.014214	0.002434	5.20E-09	A	AC	0.420	NA
rs11127048	2	-0.019970	0.002398	8.10E-17	G	A	0.381	0.002
rs7571970	2	-0.020760	0.003047	9.40E-12	T	C	0.173	
rs35198275	3	0.020797	0.003387	8.20E-10	A	G	0.866	0.000
rs2726513	4	0.015301	0.002347	7.00E-11	G	T	0.587	0.000
rs12514566	5	0.014831	0.002428	1.00E-09	G	A	0.665	0.004
rs6893807	5	-0.019160	0.003159	1.30E-09	A	G	0.844	
rs1327259	6	0.015707	0.002365	3.10E-11	A	G	0.614	0.000
rs4410790	7	-0.051710	0.002384	2.80E-104	T	C	0.364	0.003
rs34060476	7	-0.025190	0.003365	7.10E-14	A	G	0.866	0.006
rs1057868	7	-0.024450	0.002536	5.50E-22	C	T	0.714	0.000
rs76881016	10	-0.025840	0.004453	6.60E-09	A	G	0.928	0.001
rs117810762	10	-0.050110	0.008765	1.10E-08	G	A	0.982	
rs2298527	11	0.014947	0.002336	1.60E-10	G	C	0.406	0.000
rs2472297	15	-0.065210	0.002587	3.10E-140	C	T	0.732	0.006
rs2521501	15	0.014916	0.002472	1.60E-09	A	T	0.677	
rs201399553	16	-0.022530	0.002377	2.60E-21	T	TTC	0.594	0.000
rs531431865	17	-0.017100	0.002507	9.00E-12	C	CA	0.679	0.000
rs139937261	17	-0.014910	0.002304	9.80E-11	C	CG	0.488	
rs66723169	18	-0.022490	0.002732	1.80E-16	C	A	0.769	0.000
rs56113850	19	-0.020710	0.002323	4.90E-19	T	C	0.422	0.000
rs6063085	20	-0.015710	0.002370	3.40E-11	A	C	0.625	0.000
rs181251778	22	0.072648	0.009834	1.50E-13	A	G	0.986	0.000

Table 4.9. Characteristics of genetic instruments associated with tea consumption cups per day

SNP	Chromosome	beta	se	p-value	effect_allele	other_allele	eaf	R^2
rs77476394	1	-0.015720	0.002785	1.60E-08	CTAAA	C	0.209	0.002
rs11204711	1	-0.014440	0.002336	6.40E-10	A	G	0.616	0.000
rs56188862	1	0.016829	0.002297	2.40E-13	T	C	0.615	0.000
rs2117137	3	-0.013490	0.002273	2.90E-09	A	G	0.595	0.004
rs145755097	3	-0.018490	0.003258	1.40E-08	C	CTTTGT	0.853	
rs1481012	4	0.026005	0.003536	1.90E-13	A	G	0.887	0.000
rs192084998	5	0.015836	0.002458	1.20E-10	G	A	0.704	0.000
rs2465018	6	-0.022110	0.002666	1.10E-16	G	A	0.769	0.001
rs139797380	6	0.075145	0.013059	8.70E-09	C	G	0.992	
rs4410790	7	-0.040330	0.002320	1.10E-67	T	C	0.365	0.005
rs141180025	7	-0.013990	0.002335	2.10E-09	CT	C	0.391	0.005
rs17685	7	-0.023910	0.002488	7.20E-22	G	A	0.722	0.000
rs62534435	9	-0.016290	0.002776	4.40E-09	C	G	0.797	0.000
rs10741694	11	-0.014740	0.002312	1.80E-10	T	C	0.373	0.001
rs11022752	11	-0.015350	0.002525	1.20E-09	A	G	0.731	
rs73053413	12	0.021681	0.003021	7.10E-13	C	T	0.837	0.000
rs12591786	15	0.019576	0.003109	3.00E-10	C	T	0.842	0.000
rs2472297	15	-0.054110	0.002521	3.30E-102	C	T	0.733	
rs28429148	16	0.013189	0.002288	8.20E-09	G	A	0.565	0.007
rs199602679	16	-0.016030	0.002800	1.00E-08	G	GT	0.784	
rs140775622	20	-0.022620	0.003207	1.80E-12	C	T	0.831	0.000
rs4817505	21	-0.015120	0.002292	4.20E-11	T	C	0.608	0.000
rs9624470	22	-0.025340	0.002272	6.80E-29	G	A	0.419	0.001
rs132919	22	-0.017080	0.002697	2.40E-10	G	C	0.226	

Table 5. Dose-response relationship between addictive behaviors and gallstone disease risk with linear or spline model

	Linear model (RR_l, 95% CI)			Cubic spline moel (RR_s, 95% *CI*)			Testparm *P* value
	per 1 unit	per 5 units	per 10 units	doses 1	doses 2	doses 3	
Smoking cigarettes/day	1.01 (1.01-1.01)	1.05 (1.04-1.06)	1.10 (1.08-1.12)	1.04 (0.99-1.10)	0.03 ($6.53*10^{-6}$-106.10)	55.44 (0.01-585832.2)	0.001
Smoking pack-years	1.01 (1.01-1.01)	1.04 (1.03-1.05)	1.08 (1.06-1.09)	1.01 (1.01-1.02)	0.97 (0.93-1.00)	1.05 (0.98-1.13)	0.082
Alcohol intake grams/day	0.99 (0.96-1.01)	0.94 (0.83-1.07)	0.89 (0.69-1.15)	0.99 (0.98-0.99)	1.12 (0.87-1.43)	0.89 (0.64-1.23)	0.000
Alcohol intake times/week	0.96 (0.92-1.00)	0.81 (0.67-0.99)	0.66 (0.45-0.99)	0.96 (0.96-0.97)	0.50 (0.20-1.26)	2.33 (0.81-6.70)	0.000
Alcohol frequency days/week	0.96 (0.94-0.97)	0.80 (0.75-0.86)	——	0.96 (0.90-1.03)	1.00 (0.64-1.58)	0.96 (0.35-2.66)	0.893
Coffee consumption cups/day	0.95 (0.94-0.96)	0.77 (0.74-0.82)	——	0.91 (0.88-0.95)	1.21 (0.92-1.61)	0.77 (0.48-1.24)	0.020
Tea consumption cups/day	1.02 (0.94-1.10)	1.10 (0.75-1.63)	——	1.18 (1.07-1.31)	0.07 (0.01-0.41)	31.60 (3.20-311.86)	0.012

Table 6. MR-analysis between smoking substance-intake and GSD risks.

Exposure	Study	Consortium	Population	Sample Size	Outcome	nSNPs	*OR (95% CI), P-val*	PMID
**Nicotine**	Expanding the genetic architecture of nicotine dependence and its shared genetics with multiple traits (*Nat Commun., 2020*)	iNDiGO	European ancestry or African American	58,000	**cholelithiasis**	**4**	**1.012 (1.002-1.022), 0.017**	33144568
					gallstone	4	1.002 (0.994-1.010), 0.604	
					cholecystectomy	4	1.006 (0.995-1.018), 0.290	
**Cannabis**	GWAS of lifetime cannabis use reveals new risk loci, genetic overlap with psychiatric traits,and a causal influence of schizophrenia (*Nat Neurosci., 2018*)	ICC, 23andMe, and UK-Biobank	European ancestry	184,765	cholelithiasis	7	1.000 (0.995-1.004), 0.835	30150663
					gallstone	8	1.002 (0.997-1.007), 0.441	
					cholecystectomy	8	1.003 (0.996-1.010), 0.414	
**eCO**	The genetics of smoking in individuals with chronic obstructive pulmonary disease (*Respiratory Research, 2018*)	the Lung Health Study (LHS)	European Americans	2,706	cholelithiasis	1	0.995 (0.989-1.001), 0.103	29631575
					gallstone	1	1.000 (0.995-1.004), 0.843	
					cholecystectomy	1	0.997 (0.990-1.003), 0.325	
**Exposure to tobacco smoke (ETS)**	*MR-Base (ukb-b-6244)* : output from GWAS pipeline using Phesant derived variables from UKBiobank, 2018.	MRC-IEU	European	391,502	cholelithiasis	7	1.001 (0.965-1.039), 0.946	NA
					gallstone	7	1.017 (0.991-1.045), 0.204	
					**cholecystectomy**	**7**	**1.043 (1.002-1.086), 0.038**	

eCO (exhaled carbon monoxide): a simple breath test of tobacco smoke intake.

Table 7. MR-analysis between caffeine consumption and GSD risks.

Exposure	Study	Consortium	Population	Sample Size	Outcome	nSNPs	*OR (95% CI), P-val*	PMID
**Self-reported coffee consumption**	A genome-wide association study of bitter and sweet beverage consumption (*Hum Mol Genet, 2019*)	UK Biobank 24h recall data & assessment center data	European	335,909	cholelithiasis	32	1.002 (0.989-1.014), 0.787	31046077
					gallstone	29	1.003 (0.992-1.015), 0.581	
					cholecystectomy	30	1.008 (0.984-1.032), 0.504	

Continued

Exposure	Study	Consortium	Population	Sample Size	Outcome	nSNPs	*OR (95% CI), P-val*	PMID
**Habitual caffeine intake**	Genome-wide meta-analysis identifies six novel loci associated with habitual coffee consumption (*Mol Psychiatry, 2015*)	the ARIC study, the Busselton Health study, CHS, COLAUS, DNBC, EGCUT, EPIC-Norfolk, ERF, FamHS, Fenland, FHS, GOOD, HBCS, HealthABC, HPFS, inCHIANTI, KORA, MESA, NFBC, NHS, NTR, PLCO, RS-I, SHIP, SORBS, THISEAS, TwinGene, WGHS,	European ancestry	91,462	**cholelithiasis**	**6**	**0.997 (0.995-1.000), 0.044**	25288136
					gallstone	6	0.998 (0.995-1.002), 0.368	
					**cholecystectomy**	**6**	**0.996 (0.993-0.999), 0.010**	
				30,062	**cholelithiasis**	**3**	**0.993 (0.988-0.997), 0.003**	
					**gallstone**	**3**	**0.995 (0.992-0.999), 0.021**	
					**cholecystectomy**	**3**	**0.992 (0.986-0.997), 0.002**	
**Caffeine-metabolism**	Genome-wide association study of caffeine metabolites provides new insights to caffeine metabolism and dietary caffeine-consumption behavior (*Hum Mol Genet, 2016*)	PIVUS, SHIP-TREND, SKIPOGH, TwinGene, TwinsUK, and the ULSAM	European ancestry	9,876	**cholelithiasis**	**9**	**0.997 (0.995-0.999), 0.013**	27702941
					gallstone	9	0.999 (0.997-1.001), 0.391	
					**cholecystectomy**	**10**	**0.998 (0.996-1.000), 0.025**	
**Coffee type: Decaffeinated coffee**	*MR-Base (ukb-d-1508_1)* : GWAS_from UKBiobank, 2018.	Neale lab	European	283,449	cholelithiasis	2	1.027 (0.961-1.098), 0.432	NA
					**gallstone**	**1**	**1.064 (1.000-1.131), 0.048**	
					**cholecystectomy**	**2**	**1.127 (1.043-1.217), 0.002**	
**Coffee type: Instant coffee**	*MR-Base (ukb-d-1508_2)* : GWAS_from UKBiobank, 2018.	Neale lab	European	283,449	**cholelithiasis**	**3**	**1.074 (1.016-1.135), 0.012**	NA
					gallstone	3	1.040 (0.998-1.083), 0.060	
					**cholecystectomy**	**3**	**1.083 (1.015-1.156), 0.015**	
**Tea consumption** (substances other than caffeine: **Polyphenols**)	*MR-Base (ukb-b-4078)* : Green tea intake_GWAS from UKBiobank, 2018.	MRC-IEU	European	64,949	**gallstones**	**1**	**0.999 (0.998-1.000), 0.042**	NA

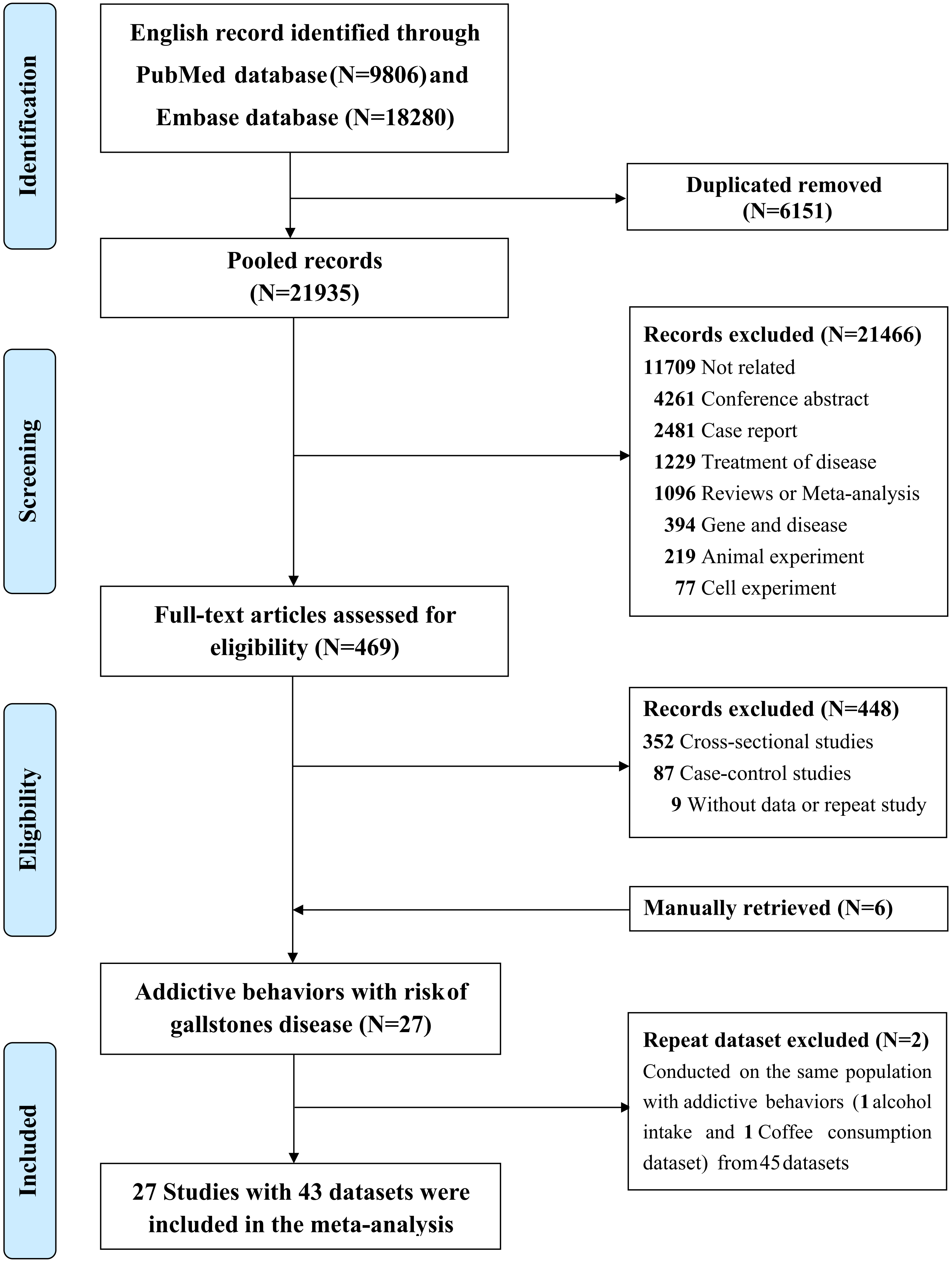

Figure 1. Flowchart of study selection for inclusion in meta-analysis

2A

Study name	Risk ratio	Lower limit	Upper limit	Z-Value	p-Value
Stampfer-1992-1	1.06	0.96	1.17	1.15	0.25
Stampfer-1992-2	1.17	1.07	1.27	3.59	0.00
Kato-1992-1	1.10	0.84	1.44	0.70	0.48
Kato-1992-2	1.30	1.06	1.59	2.52	0.01
Murray-1994	1.19	1.06	1.34	2.91	0.00
Grodstein-1994-1	1.10	0.86	1.41	0.75	0.45
Grodstein-1994-2	1.30	1.00	1.69	1.94	0.05
Misciagna-1996	2.15	1.31	3.53	3.02	0.00
Sahi-1998-1	1.28	0.89	1.85	1.32	0.19
Sahi-1998-2	1.47	1.13	1.92	2.85	0.00
Syngal-1999	1.05	0.92	1.20	0.72	0.47
Boland-2002-1	1.08	0.75	1.55	0.42	0.68
Boland-2002-2	1.13	0.70	1.81	0.51	0.61
Boland-2002-3	0.89	0.67	1.18	-0.82	0.41
Boland-2002-4	0.78	0.57	1.06	-1.57	0.12
Yamada-2005	1.19	1.02	1.39	2.15	0.03
Gonzalez-2007-1	1.18	0.99	1.41	1.83	0.07
Gonzalez-2007-2	1.05	0.92	1.20	0.72	0.47
Katsika-2007-1	1.15	0.95	1.39	1.44	0.15
Katsika-2007-2	0.99	0.78	1.26	-0.08	0.94
Liu-2009-1	1.10	1.06	1.14	5.13	0.00
Liu-2009-2	1.22	1.18	1.26	11.88	0.00
Halldestam-2009	1.18	0.43	3.24	0.32	0.75
Etminan-2011	2.06	1.99	2.13	41.67	0.00
Shabanzadeh-2016	0.84	0.63	1.12	-1.19	0.23
Figueiredo-2017-1	1.12	1.06	1.18	4.14	0.00
Figueiredo-2017-2	1.19	1.10	1.29	4.28	0.00
Figueiredo-2017-3	1.14	1.09	1.19	5.85	0.00
Figueiredo-2017-4	1.23	1.16	1.31	6.67	0.00
Kim-2018	1.18	0.79	1.76	0.82	0.41
	1.17	1.06	1.29	3.07	0.00

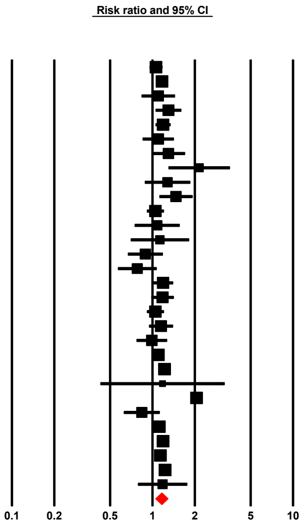

2B

Study name	Risk ratio	Lower limit	Upper limit	Z-Value	p-Value
Maclure-1989	0.89	0.81	0.98	-2.40	0.02
Sichieri-1990-1	0.89	0.69	1.14	-0.91	0.36
Sichieri-1990-2	0.98	0.82	1.18	-0.22	0.83
Kato-1992	0.80	0.61	1.05	-1.59	0.11
Moerman-1994	0.70	0.44	1.11	-1.51	0.13
Misciagna-1996	0.71	0.54	0.93	-2.52	0.01
Leitzmann-1999-1	0.83	0.76	0.91	-4.05	0.00
Leitzmann-1999-2	0.82	0.74	0.91	-3.76	0.00
Boland-2002-1	1.52	0.88	2.63	1.49	0.14
Boland-2002-2	0.98	0.71	1.36	-0.12	0.90
Boland-2002-3	0.76	0.51	1.14	-1.33	0.18
Boland-2002-4	0.88	0.73	1.06	-1.34	0.18
Leitzmann-2003-1	0.70	0.65	0.75	-9.77	0.00
Leitzmann-2003-2	0.86	0.83	0.89	-8.47	0.00
Yamada-2005	1.02	0.89	1.17	0.28	0.78
Gonzalez-2007	0.79	0.73	0.86	-5.64	0.00
Katsika-2007-1	0.93	0.83	1.04	-1.26	0.21
Katsika-2007-2	0.57	0.53	0.61	-15.67	0.00
Liu-2009	0.76	0.75	0.77	-40.88	0.00
Halldestam-2009	0.29	0.09	0.96	-2.03	0.04
Banim-2011-1	0.89	0.60	1.33	-0.57	0.57
Banim-2011-2	0.93	0.71	1.22	-0.53	0.60
Shabanzadeh-2016	0.99	0.98	1.00	-1.95	0.05
Figueiredo-2017-1	0.89	0.85	0.93	-5.08	0.00
Figueiredo-2017-2	0.86	0.82	0.90	-6.35	0.00
Kim-2018	0.81	0.56	1.17	-1.12	0.26
	0.84	0.78	0.91	-4.53	0.00

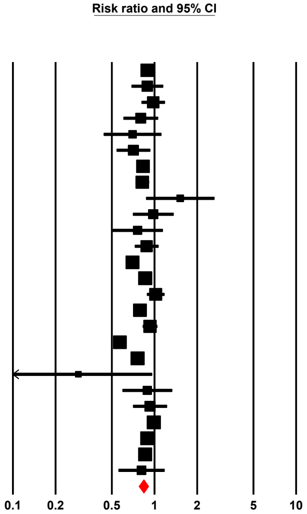

2C

Study name	Risk ratio	Lower limit	Upper limit	Z-Value	p-Value
Misciagna-1996	0.62	0.40	0.97	-2.09	0.04
Leitzmann-1999	0.73	0.60	0.89	-3.13	0.00
Leitzmann-2002	0.89	0.86	0.92	-6.88	0.00
Banim-2011-1	0.58	0.38	0.89	-2.48	0.01
Banim-2011-2	0.84	0.60	1.17	-1.02	0.31
Nordenvall-2014-1	0.99	0.88	1.12	-0.16	0.87
Nordenvall-2014-2	0.75	0.67	0.84	-4.99	0.00
Shabanzadeh-2016	1.00	0.97	1.03	0.00	1.00
Nordestgaard-2019	0.86	0.79	0.94	-3.32	0.00
	0.86	0.79	0.93	-3.64	0.00

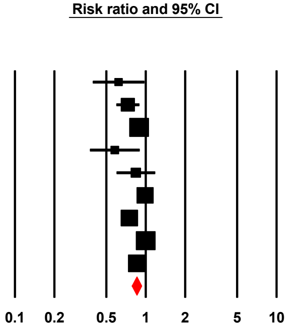

2D

Study name	Risk ratio	Lower limit	Upper limit	Z-Value	p-Value
Leitzmann-1999-1-1	1.13	0.99	1.29	1.81	0.07
Leitzmann-1999-1-2	0.87	0.66	1.15	-0.98	0.33
Leitzmann-1999-1-3	1.42	0.92	2.19	1.59	0.11
Leitzmann-2002-1-1	1.07	1.02	1.12	2.71	0.01
Leitzmann-2002-1-2	1.06	0.98	1.14	1.51	0.13
Leitzmann-2002-1-3	1.13	1.00	1.28	1.94	0.05
	1.08	1.04	1.12	3.87	0.00

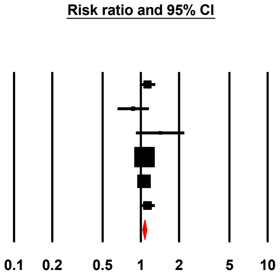

Figure 2. Addictive behaviors overall pooled effect for gallstone disease (A: cigarette smoking, B: alcohol intake, C: coffee consumption, D: tea consumption)

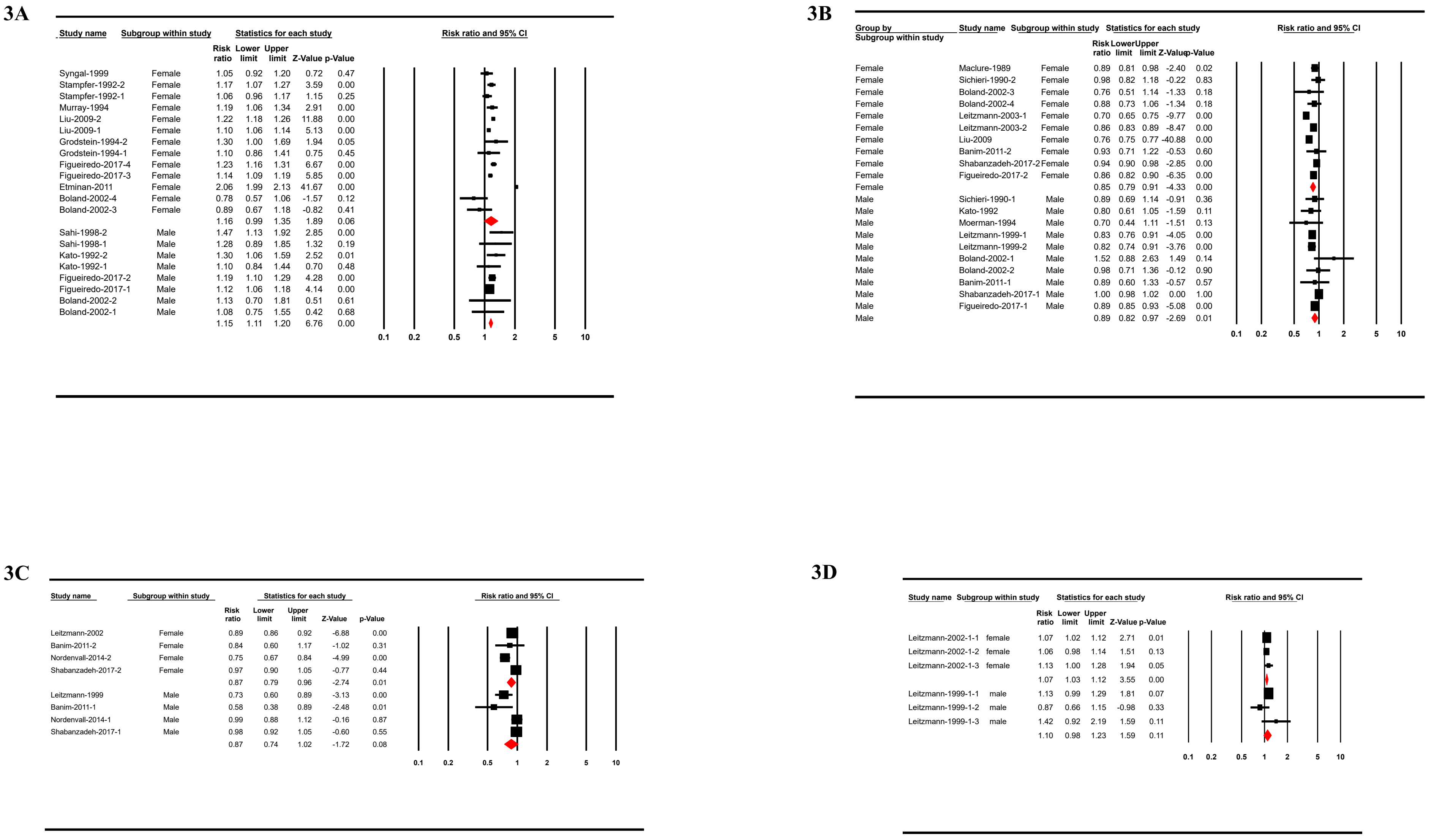

Figure 3. Forest plots of studies on addictive behaviors and the risk of gallstones with subgroup-analysis by gender (A. smoking, B. alcohol, C. coffee, D. tea)

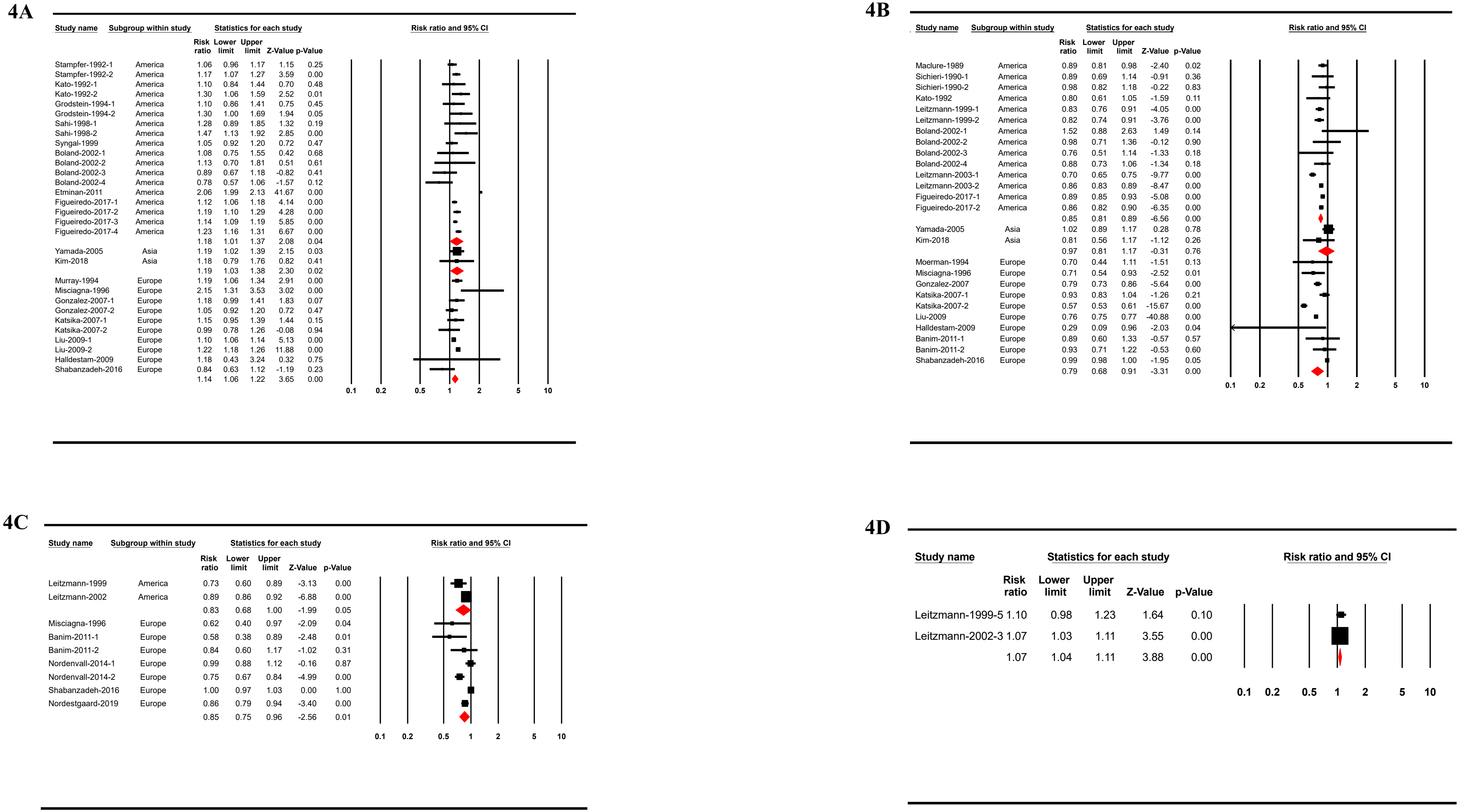

Figure 4. Forest plots of studies on addictive behaviors and the risk of gallstones with subgroup-analysis by geographic (A. smoking, B. alcohol, C. coffee, D. tea).

*(Tea consumption group only included studies on the population from America)

5A

Study name	Subgroup within study	Risk ratio	Lower limit	Upper limit	Z-Value	p-Value
Stampfer-1992-5	with Cholecystectomy	1.06	0.94	1.19	0.99	0.32
Stampfer-1992-6	with Cholecystectomy	1.15	1.11	1.19	7.87	0.00
Syngal-1999	with Cholecystectomy	1.05	0.92	1.20	0.72	0.47
Etminan-2011	with Cholecystectomy	2.06	1.99	2.13	41.67	0.00
		1.28	0.87	1.88	1.23	0.22
Stampfer-1992-3	without Cholecystectomy	1.09	0.89	1.33	0.84	0.40
Stampfer-1992-4	without Cholecystectomy	1.08	0.89	1.31	0.80	0.43
Yamada-2005	without Cholecystectomy	1.19	1.02	1.39	2.15	0.03
		1.13	1.02	1.25	2.29	0.02

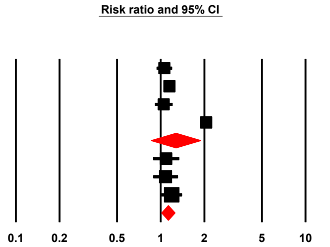

5B

Study name	Subgroup within study	Risk ratio	Lower limit	Upper limit	Z-Value	p-Value
Stampfer-1992-2	Current smoker	1.17	1.07	1.27	3.59	0.00
Kato-1992-2	Current smoker	1.30	1.06	1.59	2.52	0.01
Grodstein-1994-2	Current smoker	1.30	1.00	1.69	1.94	0.05
Sahi-1998-2	Current smoker	1.47	1.13	1.92	2.85	0.00
Boland-2002-2	Current smoker	1.13	0.70	1.81	0.51	0.61
Boland-2002-4	Current smoker	0.78	0.57	1.06	-1.57	0.12
Gonzalez-2007-2	Current smoker	1.05	0.92	1.20	0.72	0.47
Katsika-2007-2	Current smoker	0.99	0.78	1.26	-0.08	0.94
Liu-2009-2	Current smoker	1.22	1.18	1.26	11.88	0.00
Figueiredo-2017-2	Current smoker	1.19	1.10	1.29	4.28	0.00
Figueiredo-2017-4	Current smoker	1.23	1.16	1.31	6.67	0.00
		1.18	1.13	1.24	6.88	0.00
Stampfer-1992-1	Former smoker	1.06	0.96	1.17	1.15	0.25
Kato-1992-1	Former smoker	1.10	0.84	1.44	0.70	0.48
Grodstein-1994-1	Former smoker	1.10	0.86	1.41	0.75	0.45
Sahi-1998-1	Former smoker	1.28	0.89	1.85	1.32	0.19
Boland-2002-1	Former smoker	1.08	0.75	1.55	0.42	0.68
Boland-2002-3	Former smoker	0.89	0.67	1.18	-0.82	0.41
Gonzalez-2007-1	Former smoker	1.18	0.99	1.41	1.83	0.07
Katsika-2007-1	Former smoker	1.15	0.95	1.39	1.44	0.15
Liu-2009-1	Former smoker	1.10	1.06	1.14	5.13	0.00
Figueiredo-2017-1	Former smoker	1.12	1.06	1.18	4.14	0.00
Figueiredo-2017-3	Former smoker	1.14	1.09	1.19	5.85	0.00
		1.11	1.09	1.14	9.06	0.00

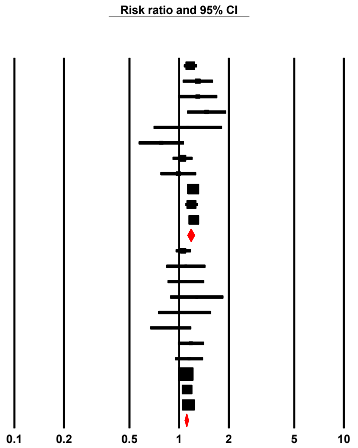

5C

Study name	Subgroup within study	Risk ratio	Lower limit	Upper limit	Z-Value	p-Value
Kato-1992-2-2	> 20 pack-years	1.30	1.00	1.69	1.94	0.05
Kato-1992-2-3	> 20 pack-years	1.40	1.09	1.79	2.68	0.01
Figueiredo-2017-2-2	> 20 pack-years	1.22	1.09	1.37	3.41	0.00
Figueiredo-2017-4-2	> 20 pack-years	1.37	1.23	1.53	5.65	0.00
		1.31	1.21	1.40	7.22	0.00
Kato-1992-2-1	≤ 20 pack-years	1.00	0.79	1.26	0.00	1.00
Figueiredo-2017-2-1	≤ 20 pack-years	1.16	1.04	1.30	2.61	0.01
Figueiredo-2017-4-1	≤ 20 pack-years	1.17	1.08	1.27	3.80	0.00
		1.15	1.08	1.23	4.43	0.00

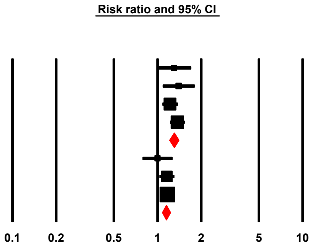

Figure 5. Forest plots of subgroup analysis for studies on smoking status and gallstone disease risks

A. with cholecystectomy *vs.* without cholecystectomy, B. current smokers *vs.* former smokers

C. smoking >20 *vs.* ≤ 20 pack-years

6A

Study name	Subgroup within study	Risk ratio	Lower limit	Upper limit	Z-Value	p-Value
Leitzmann-2003-2	with Cholecystectomy	0.86	0.83	0.89	-8.47	0.00
Leitzmann-2003-3	with Cholecystectomy	0.88	0.83	0.93	-4.40	0.00
		0.87	0.84	0.89	-9.52	0.00
Leitzmann-2003-4	without Cholecystectomy	0.70	0.65	0.75	-9.77	0.00
Yamada-2005	without Cholecystectomy	1.02	0.89	1.17	0.28	0.78
		0.84	0.58	1.22	-0.92	0.36

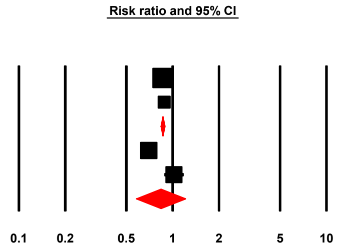

6B

Study name	Subgroup within study	Risk ratio	Lower limit	Upper limit	Z-Value	p-Value
Misciagna-1996-2	Beer	1.11	0.60	2.05	0.33	0.74
Leitzmann-1999-4	Beer	0.75	0.62	0.91	-2.94	0.00
Leitzmann-2003-5	Beer	0.88	0.76	1.02	-1.70	0.09
		0.84	0.73	0.96	-2.50	0.01
Misciagna-1996-3	Liquor	0.78	0.45	1.35	-0.89	0.37
Leitzmann-1999-5	Liquor	0.79	0.68	0.92	-2.95	0.00
Leitzmann-2003-6	Liquor	0.84	0.75	0.94	-3.03	0.00
		0.82	0.75	0.90	-4.27	0.00
Misciagna-1996-1	Wine	0.79	0.61	1.03	-1.76	0.08
Leitzmann-1999-3	Wine	0.86	0.74	1.00	-1.90	0.06
Leitzmann-2003-4	Wine	0.88	0.81	0.95	-3.14	0.00
		0.87	0.81	0.93	-4.00	0.00

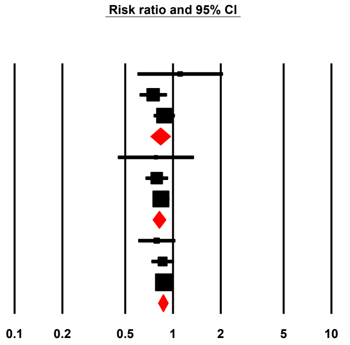

6C

Study name	Subgroup within study	Risk ratio	Lower limit	Upper limit	Z-Value	p-Value
Leitzmann-1999-2-1	1-2 days/week	0.96	0.83	1.12	-0.53	0.60
Leitzmann-2003-3-1	1-2 days/week	0.94	0.86	1.02	-1.42	0.16
		0.94	0.88	1.02	-1.50	0.13
Leitzmann-1999-2-2	3-4 days/week	0.79	0.65	0.97	-2.31	0.02
Leitzmann-2003-3-2	3-4 days/week	0.88	0.77	1.00	-1.96	0.05
		0.85	0.77	0.95	-2.90	0.00
Leitzmann-1999-2-3	5-7 days/week	0.69	0.58	0.82	-4.20	0.00
Leitzmann-2003-3-3	5-7 days/week	0.87	0.76	0.99	-2.06	0.04
Leitzmann-2003-3-4	5-7 days/week	0.73	0.63	0.84	-4.29	0.00
		0.77	0.66	0.88	-3.74	0.00

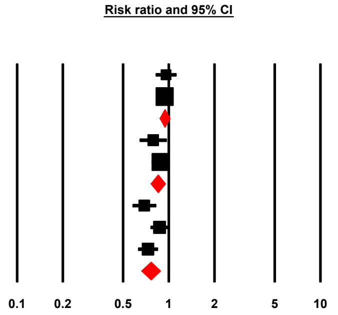

6D

Study name	Subgroup within study	Risk ratio	Lower limit	Upper limit	Z-Value	p-Value
Leitzmann-1999-3-1	< 15 grams/day	0.98	0.82	1.18	-0.22	0.83
Leitzmann-1999-4-1	< 15 grams/day	0.79	0.62	1.00	-1.93	0.05
Leitzmann-1999-5-1	< 15 grams/day	0.84	0.68	1.04	-1.57	0.12
Leitzmann-2003-4-1	< 15 grams/day	0.89	0.82	0.97	-2.72	0.01
Leitzmann-2003-5-1	< 15 grams/day	1.03	0.87	1.22	0.33	0.74
Leitzmann-2003-6-1	< 15 grams/day	0.90	0.79	1.03	-1.56	0.12
		0.91	0.85	0.96	-3.39	0.00
Leitzmann-1999-3-2	≥ 15 grams/day	0.59	0.43	0.81	-3.27	0.00
Leitzmann-1999-4-2	≥ 15 grams/day	0.68	0.50	0.93	-2.40	0.02
Leitzmann-1999-5-2	≥ 15 grams/day	0.75	0.60	0.93	-2.61	0.01
Leitzmann-2003-4-2	≥ 15 grams/day	0.79	0.65	0.96	-2.37	0.02
Leitzmann-2003-5-2	≥ 15 grams/day	0.57	0.43	0.76	-3.87	0.00
Leitzmann-2003-6-2	≥ 15 grams/day	0.72	0.59	0.87	-3.32	0.00
		0.71	0.64	0.78	-7.05	0.00

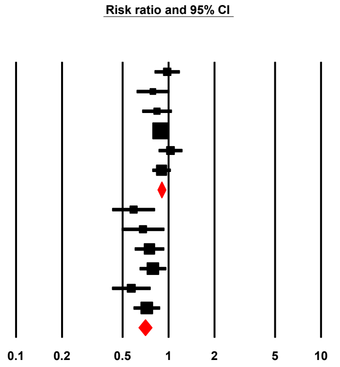

Figure 6. Forest plots of subgroup analysis for studies on alcohol intake and gallstonedisease risks

(A. with cholecystectomy vs. without cholecystectomy, B. differ in three common type of alcohol: beer, liquor, and wine, C. differ in the frequency on drinking days per week, D. drinking ≥15 vs. <15 grams per day).

7A

Study name	Subgroup within study	Risk ratio	Lower limit	Upper limit	Z-Value	p-Value
Leitzmann-1999-3	Caffeinated coffee intake	0.80	0.73	0.88	-4.68	0.00
Leitzmann-2002-1	Caffeinated coffee intake	0.85	0.82	0.88	-9.17	0.00
		0.84	0.82	0.87	-10.23	0.00
Leitzmann-1999-4	Decaffeinated coffee intake	1.05	0.95	1.17	0.92	0.36
Leitzmann-2002-2	Decaffeinated coffee intake	0.99	0.94	1.04	-0.39	0.70
		1.00	0.96	1.05	0.05	0.96

Statistics for each study / Risk ratio and 95% CI

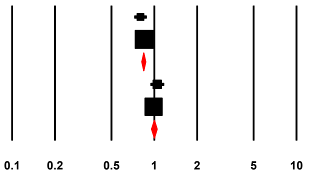

7B

Study name	Subgroup within study	Risk ratio	Lower limit	Upper limit	Z-Value	p-Value
Leitzmann-1999-1-1	≤ 1 cup/day	0.88	0.67	1.15	-0.94	0.35
Leitzmann-2002-1-1	≤ 1 cup/day	0.92	0.87	0.97	-2.87	0.00
		0.92	0.87	0.97	-3.00	0.00
Nordestgaard-2019-3	≥ 6 cups/day	0.77	0.61	0.97	-2.26	0.02
		0.77	0.61	0.97	-2.26	0.02
Leitzmann-1999-1-2	2-3 cups/day	0.60	0.42	0.86	-2.79	0.01
Leitzmann-2002-1-2	2-3 cups/day	0.82	0.78	0.86	-7.52	0.00
		0.81	0.77	0.86	-7.84	0.00
Nordestgaard-2019-2	3-6 cups/day	0.83	0.71	0.97	-2.34	0.02
		0.83	0.71	0.97	-2.34	0.02

Statistics for each study / Risk ratio and 95% CI

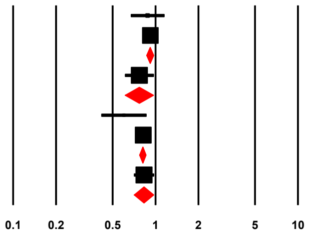

7C

Study name	Subgroup within study	Risk ratio	Lower limit	Upper limit	Z-Value	p-Value
Leitzmann-1999-1	≤ 1 cup/day	1.13	0.99	1.29	1.81	0.07
Leitzmann-2002-1	≤ 1 cup/day	1.07	1.02	1.12	2.71	0.01
		1.08	1.03	1.13	3.17	0.00
Leitzmann-1999-3	≥ 4 cups/day	1.42	0.92	2.19	1.59	0.11
Leitzmann-2002-3	≥ 4 cups/day	1.13	1.00	1.28	1.94	0.05
		1.15	1.02	1.29	2.30	0.02
Leitzmann-1999-2	2-3 cups/day	0.87	0.66	1.15	-0.98	0.33
Leitzmann-2002-2	2-3 cups/day	1.06	0.98	1.14	1.51	0.13
		1.05	0.97	1.12	1.20	0.23

Statistics for each study / Risk ratio and 95% CI

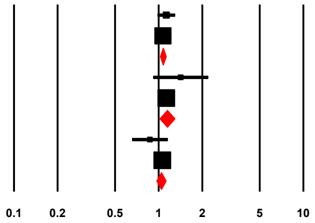

Figure 7. Forest plots of subgroup analysis for studies on coffee/tea consumption and gallstone disease risks (A. caffeinated coffee intake *vs.* decaffeinated coffee intake, B. differ in the frequency on coffee consumption by cups per day, C. tea consumption cups per day.

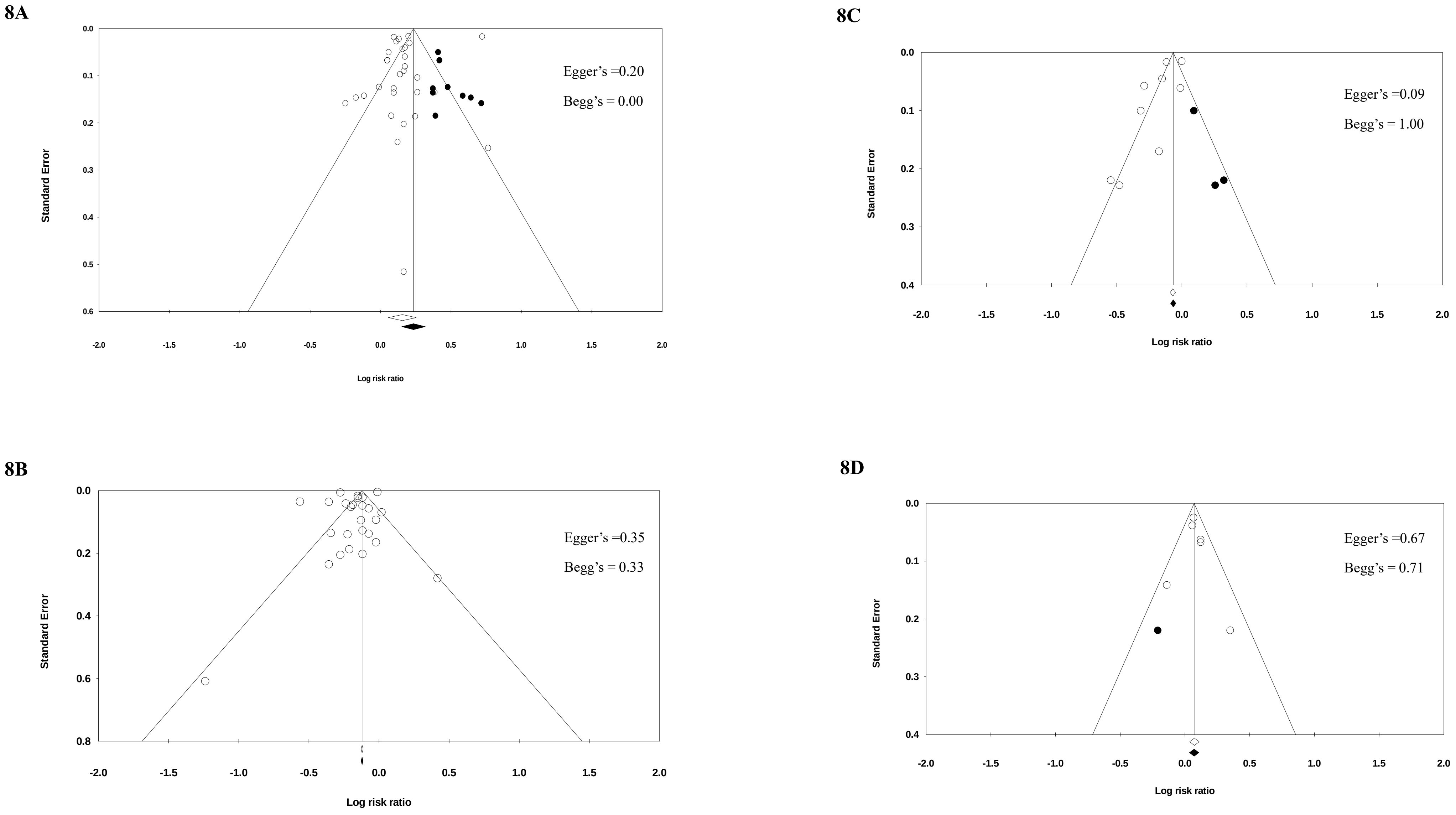

Figure 8. Funnel plot of studies on addictive behaviors and the risk of gallstones

A: cigarette smoking (adjusted RR=1.26, 95%CI: 1.16-1.37);

B: alcohol intake (adjusted RR=0.84, 95%CI: 0.78-0.91);

C: coffee consumption (adjusted RR=0.89, 95%CI: 0.83-0.97);

D: tea consumption (adjusted RR=1.07, 95%CI: 1.03-1.11).

A1

Study name	Statistics with study removed					Risk ratio (95% CI) with study removed
	Point	Lower limit	Upper limit	Z-Value	p-Value	
Stampfer-1992-1	1.17	1.06	1.30	3.07	0.00	
Stampfer-1992-2	1.17	1.05	1.29	2.96	0.00	
Kato-1992-1	1.17	1.06	1.29	3.06	0.00	
Kato-1992-2	1.16	1.05	1.29	2.93	0.00	
Murray-1994	1.17	1.05	1.29	2.97	0.00	
Grodstein-1994-1	1.17	1.06	1.29	3.05	0.00	
Grodstein-1994-2	1.16	1.05	1.29	2.95	0.00	
Misciagna-1996	1.15	1.04	1.27	2.80	0.01	
Sahi-1998-1	1.16	1.05	1.29	2.98	0.00	
Sahi-1998-2	1.16	1.05	1.28	2.87	0.00	
Syngal-1999	1.17	1.06	1.30	3.08	0.00	
Boland-2002-1	1.17	1.06	1.29	3.07	0.00	
Boland-2002-2	1.17	1.06	1.29	3.05	0.00	
Boland-2002-3	1.18	1.07	1.30	3.20	0.00	
Boland-2002-4	1.18	1.07	1.31	3.27	0.00	
Yamada-2005	1.17	1.05	1.29	2.98	0.00	
Gonzalez-2007-1	1.17	1.05	1.29	3.00	0.00	
Gonzalez-2007-2	1.17	1.06	1.30	3.08	0.00	
Katsika-2007-1	1.17	1.06	1.29	3.02	0.00	
Katsika-2007-2	1.17	1.06	1.30	3.13	0.00	
Liu-2009-1	1.17	1.05	1.30	2.92	0.00	
Liu-2009-2	1.16	1.04	1.30	2.69	0.01	
Halldestam-2009	1.17	1.06	1.29	3.06	0.00	
Etminan-2011	1.14	1.11	1.18	8.04	0.00	
Shabanzadeh-2016	1.18	1.07	1.30	3.23	0.00	
Figueiredo-2017-1	1.17	1.05	1.30	2.95	0.00	
Figueiredo-2017-2	1.17	1.05	1.29	2.94	0.00	
Figueiredo-2017-3	1.17	1.05	1.30	2.89	0.00	
Figueiredo-2017-4	1.16	1.05	1.29	2.87	0.00	
Kim-2018	1.17	1.06	1.29	3.02	0.00	
	1.17	1.06	1.29	3.07	0.00	

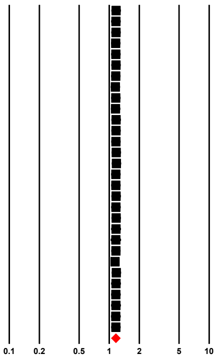

B1

Study name	Statistics with study removed					Risk ratio (95% CI) with study removed
	Point	Lower limit	Upper limit	Z-Value	p-Value	
Maclure-1989	0.84	0.78	0.91	-4.47	0.00	
Sichieri-1990-1	0.84	0.78	0.91	-4.50	0.00	
Sichieri-1990-2	0.84	0.77	0.90	-4.60	0.00	
Kato-1992	0.84	0.78	0.91	-4.42	0.00	
Moerman-1994	0.84	0.78	0.91	-4.41	0.00	
Misciagna-1996	0.85	0.78	0.91	-4.31	0.00	
Leitzmann-1999-1	0.84	0.78	0.91	-4.37	0.00	
Leitzmann-1999-2	0.84	0.78	0.91	-4.37	0.00	
Boland-2002-1	0.83	0.77	0.90	-4.72	0.00	
Boland-2002-2	0.84	0.78	0.90	-4.58	0.00	
Boland-2002-3	0.84	0.78	0.91	-4.43	0.00	
Boland-2002-4	0.84	0.78	0.91	-4.48	0.00	
Leitzmann-2003-1	0.85	0.79	0.92	-4.18	0.00	
Leitzmann-2003-2	0.84	0.78	0.91	-4.26	0.00	
Yamada-2005	0.83	0.77	0.90	-4.66	0.00	
Gonzalez-2007	0.84	0.78	0.91	-4.31	0.00	
Katsika-2007-1	0.84	0.77	0.90	-4.53	0.00	
Katsika-2007-2	0.86	0.80	0.92	-4.05	0.00	
Liu-2009	0.85	0.79	0.91	-4.66	0.00	
Halldestam-2009	0.84	0.78	0.91	-4.43	0.00	
Banim-2011-1	0.84	0.78	0.91	-4.51	0.00	
Banim-2011-2	0.84	0.78	0.90	-4.54	0.00	
Shabanzadeh-2016	0.83	0.78	0.88	-6.63	0.00	
Figueiredo-2017-1	0.84	0.78	0.91	-4.38	0.00	
Figueiredo-2017-2	0.84	0.78	0.91	-4.34	0.00	
Kim-2018	0.84	0.78	0.91	-4.45	0.00	
	0.84	0.78	0.91	-4.53	0.00	

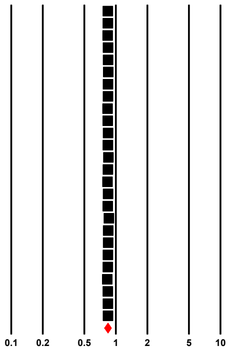

A2

Study name	Cumulative statistics					Cumulative risk ratio (95% CI)
	Point	Lower limit	Upper limit	Z-Value	p-Value	
Stampfer-1992-1	1.06	0.96	1.17	1.15	0.25	
Stampfer-1992-2	1.12	1.01	1.23	2.25	0.02	
Kato-1992-1	1.12	1.05	1.20	3.23	0.00	
Kato-1992-2	1.14	1.05	1.23	3.29	0.00	
Murray-1994	1.15	1.08	1.22	4.56	0.00	
Grodstein-1994-1	1.14	1.09	1.21	5.04	0.00	
Grodstein-1994-2	1.15	1.09	1.21	5.32	0.00	
Misciagna-1996	1.17	1.09	1.27	4.14	0.00	
Sahi-1998-1	1.18	1.09	1.26	4.45	0.00	
Sahi-1998-2	1.20	1.11	1.29	4.70	0.00	
Syngal-1999	1.18	1.10	1.26	4.54	0.00	
Boland-2002-1	1.17	1.10	1.25	4.65	0.00	
Boland-2002-2	1.17	1.10	1.24	4.81	0.00	
Boland-2002-3	1.16	1.08	1.24	4.32	0.00	
Boland-2002-4	1.14	1.06	1.23	3.60	0.00	
Yamada-2005	1.15	1.07	1.22	4.01	0.00	
Gonzalez-2007-1	1.15	1.08	1.22	4.37	0.00	
Gonzalez-2007-2	1.14	1.07	1.21	4.38	0.00	
Katsika-2007-1	1.14	1.08	1.20	4.66	0.00	
Katsika-2007-2	1.13	1.07	1.19	4.57	0.00	
Liu-2009-1	1.13	1.08	1.18	5.25	0.00	
Liu-2009-2	1.14	1.09	1.19	5.59	0.00	
Halldestam-2009	1.14	1.09	1.19	5.65	0.00	
Etminan-2011	1.18	1.04	1.34	2.54	0.01	
Shabanzadeh-2016	1.17	1.03	1.32	2.38	0.02	
Figueiredo-2017-1	1.16	1.03	1.31	2.50	0.01	
Figueiredo-2017-2	1.16	1.04	1.30	2.64	0.01	
Figueiredo-2017-3	1.16	1.05	1.29	2.82	0.00	
Figueiredo-2017-4	1.17	1.06	1.29	3.02	0.00	
Kim-2018	1.17	1.06	1.29	3.07	0.00	
	1.17	1.06	1.29	3.07	0.00	

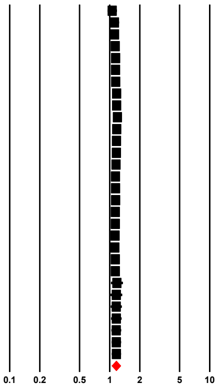

B2

Study name	Cumulative statistics					Cumulative risk ratio (95% CI)
	Point	Lower limit	Upper limit	Z-Value	p-Value	
Maclure-1989	0.89	0.81	0.98	-2.40	0.02	
Sichieri-1990-1	0.89	0.81	0.97	-2.56	0.01	
Sichieri-1990-2	0.91	0.84	0.98	-2.40	0.02	
Kato-1992	0.90	0.83	0.97	-2.75	0.01	
Moerman-1994	0.89	0.83	0.96	-2.96	0.00	
Misciagna-1996	0.87	0.81	0.95	-3.35	0.00	
Leitzmann-1999-1	0.86	0.81	0.91	-5.18	0.00	
Leitzmann-1999-2	0.85	0.81	0.89	-6.46	0.00	
Boland-2002-1	0.85	0.80	0.91	-4.66	0.00	
Boland-2002-2	0.86	0.81	0.91	-4.74	0.00	
Boland-2002-3	0.86	0.81	0.91	-5.16	0.00	
Boland-2002-4	0.86	0.81	0.90	-5.79	0.00	
Leitzmann-2003-1	0.84	0.77	0.91	-4.37	0.00	
Leitzmann-2003-2	0.84	0.78	0.89	-5.29	0.00	
Yamada-2005	0.85	0.80	0.91	-4.72	0.00	
Gonzalez-2007	0.85	0.80	0.90	-5.48	0.00	
Katsika-2007-1	0.85	0.80	0.90	-5.48	0.00	
Katsika-2007-2	0.83	0.76	0.91	-4.26	0.00	
Liu-2009	0.82	0.77	0.87	-6.09	0.00	
Halldestam-2009	0.82	0.77	0.87	-6.16	0.00	
Banim-2011-1	0.82	0.77	0.87	-6.18	0.00	
Banim-2011-2	0.82	0.77	0.88	-6.16	0.00	
Shabanzadeh-2016	0.84	0.77	0.91	-4.10	0.00	
Figueiredo-2017-1	0.84	0.78	0.91	-4.26	0.00	
Figueiredo-2017-2	0.84	0.78	0.91	-4.45	0.00	
Kim-2018	0.84	0.78	0.91	-4.53	0.00	
	0.84	0.78	0.91	-4.53	0.00	

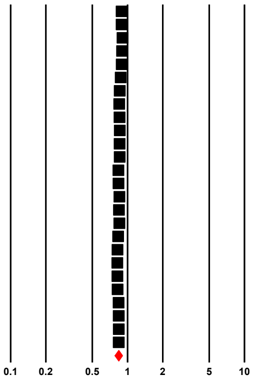

C1

Study name	Point	Lower limit	Upper limit	Z-Value	p-Value
	Statistics with study removed				
Misciagna-1996	0.87	0.80	0.94	-3.39	0.00
Leitzmann-1999	0.87	0.80	0.95	-3.16	0.00
Leitzmann-2002	0.83	0.74	0.94	-2.99	0.00
Banim-2011-1	0.87	0.80	0.94	-3.35	0.00
Banim-2011-2	0.86	0.79	0.93	-3.52	0.00
Nordenvall-2014-1	0.84	0.76	0.92	-3.83	0.00
Nordenvall-2014-2	0.88	0.81	0.96	-3.01	0.00
Shabanzadeh-2016	0.83	0.77	0.90	-4.33	0.00
Nordestgaard-2019	0.85	0.78	0.94	-3.31	0.00
	0.86	0.79	0.93	-3.64	0.00

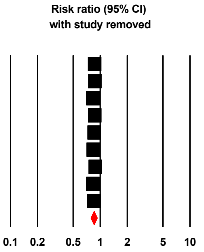

D1

Study name	Point	Lower limit	Upper limit	Z-Value	p-Value
	Statistics with study removed				
Leitzmann-1999-1-1	1.071	1.031	1.113	3.505	0.000
Leitzmann-1999-1-2	1.080	1.040	1.121	4.038	0.000
Leitzmann-1999-1-3	1.073	1.034	1.114	3.748	0.000
Leitzmann-2002-1-1	1.083	1.024	1.146	2.786	0.005
Leitzmann-2002-1-2	1.081	1.036	1.127	3.591	0.000
Leitzmann-2002-1-3	1.070	1.030	1.113	3.448	0.001
	1.076	1.037	1.116	3.871	0.000

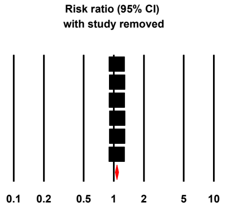

C2

Study name	Point	Lower limit	Upper limit	Z-Value	p-Value
	Cumulative statistics				
Misciagna-1996	0.62	0.40	0.97	-2.09	0.04
Leitzmann-1999	0.71	0.59	0.85	-3.71	0.00
Leitzmann-2002	0.79	0.65	0.96	-2.38	0.02
Banim-2011-1	0.75	0.61	0.92	-2.79	0.01
Banim-2011-2	0.77	0.66	0.91	-3.07	0.00
Nordenvall-2014-1	0.84	0.74	0.94	-2.94	0.00
Nordenvall-2014-2	0.82	0.73	0.91	-3.64	0.00
Shabanzadeh-2016	0.85	0.78	0.94	-3.31	0.00
Nordestgaard-2019	0.86	0.79	0.93	-3.64	0.00
	0.86	0.79	0.93	-3.64	0.00

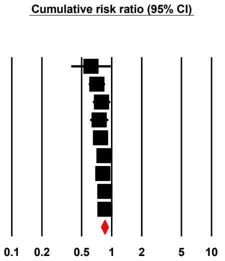

D2

Study name	Point	Lower limit	Upper limit	Z-Value	p-Value
	Cumulative statistics				
Leitzmann-1999-1-1	1.130	0.990	1.290	1.810	0.070
Leitzmann-1999-1-2	1.077	0.955	1.213	1.211	0.226
Leitzmann-1999-1-3	1.098	0.979	1.232	1.592	0.111
Leitzmann-2002-1-1	1.074	1.027	1.124	3.114	0.002
Leitzmann-2002-1-2	1.070	1.030	1.113	3.448	0.001
Leitzmann-2002-1-3	1.076	1.037	1.116	3.871	0.000
	1.076	1.037	1.116	3.871	0.000

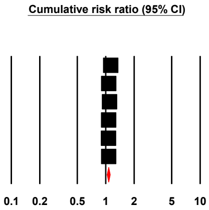

Figure 9. Forest plots of studies with removed each one and cumulative effects on addictive behaviors and gallstone disease risk

A. cigarette smoking (A1: risk ratio with study removed, A2: cumulative risk ratio),

B. alcohol intake (B1: RR with study removed, B2: cumulative RR),

C. coffee consumption (C1: the same as above, C2: the same as above),

D. tea consumption (the same as the group aboved)

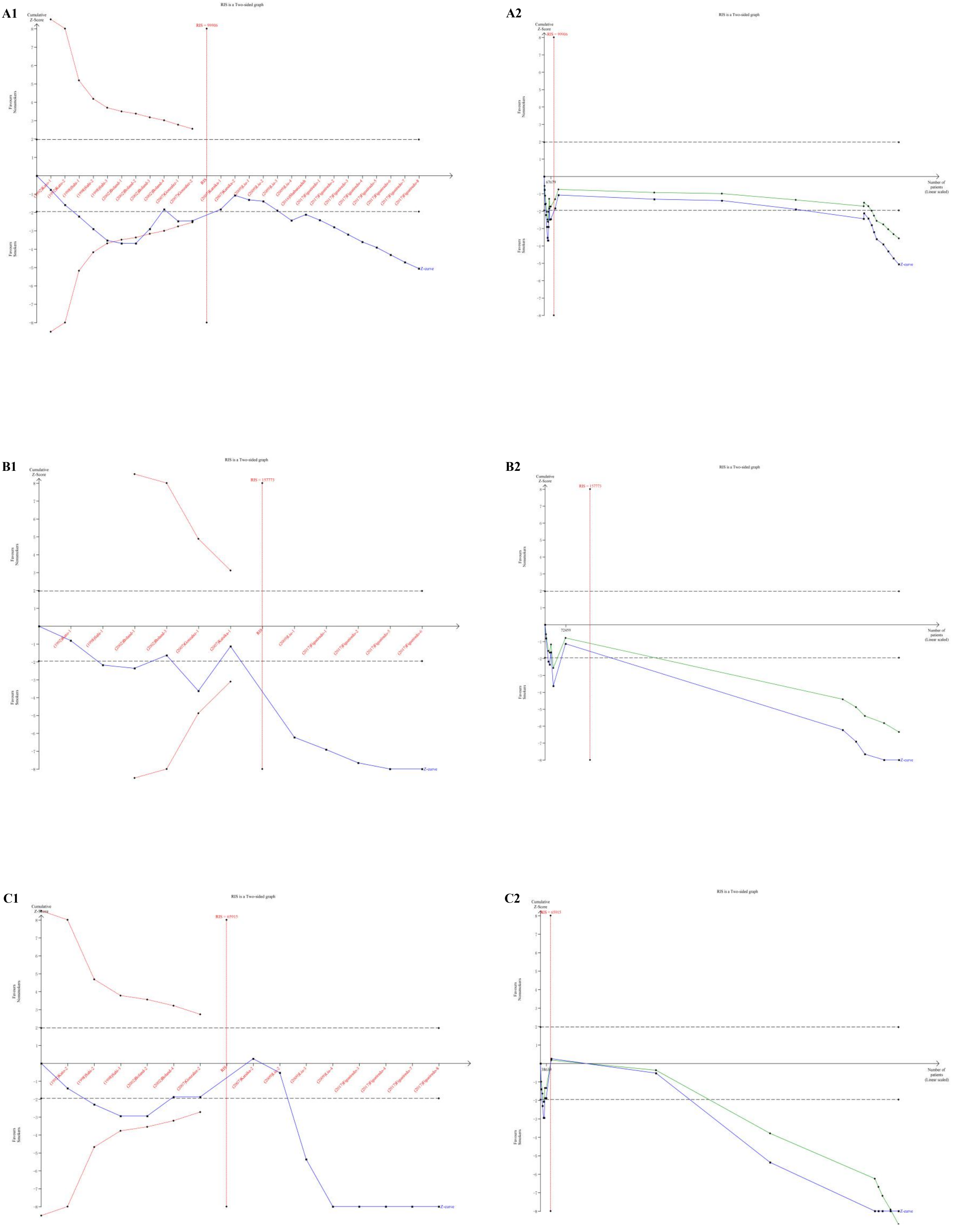
A1
RIS is a Two-sided graph
Cumulative Z-Score
RIS = 99906
Favours Nonsmokers
Favours Smokers
Z-curve
A2
RIS is a Two-sided graph
Cumulative Z-Score
RIS = 99906
67679
Number of patients (Linear scaled)
Favours Nonsmokers
Favours Smokers
Z-curve
B1
RIS is a Two-sided graph
Cumulative Z-Score
RIS = 157773
Favours Nonsmokers
Favours Smokers
Z-curve
B2
RIS is a Two-sided graph
Cumulative Z-Score
RIS = 157773
72459
Number of patients (Linear scaled)
Favours Nonsmokers
Favours Smokers
Z-curve
C1
RIS is a Two-sided graph
Cumulative Z-Score
RIS = 65915
Favours Nonsmokers
Favours Smokers
Z-curve
C2
RIS is a Two-sided graph
Cumulative Z-Score
RIS = 65915
Number of patients (Linear scaled)
Favours Nonsmokers
Favours Smokers
Z-curve

D1

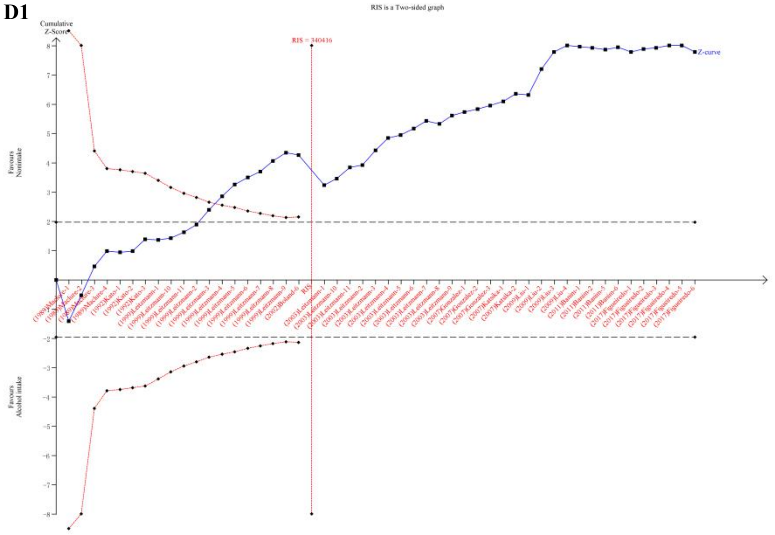

D2

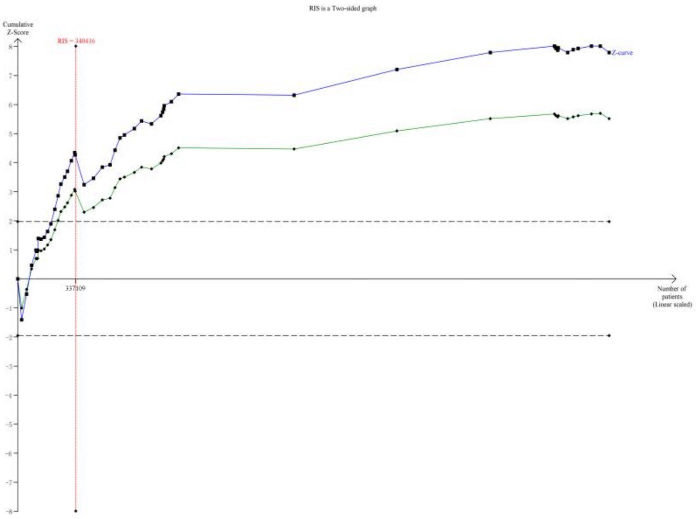

E1

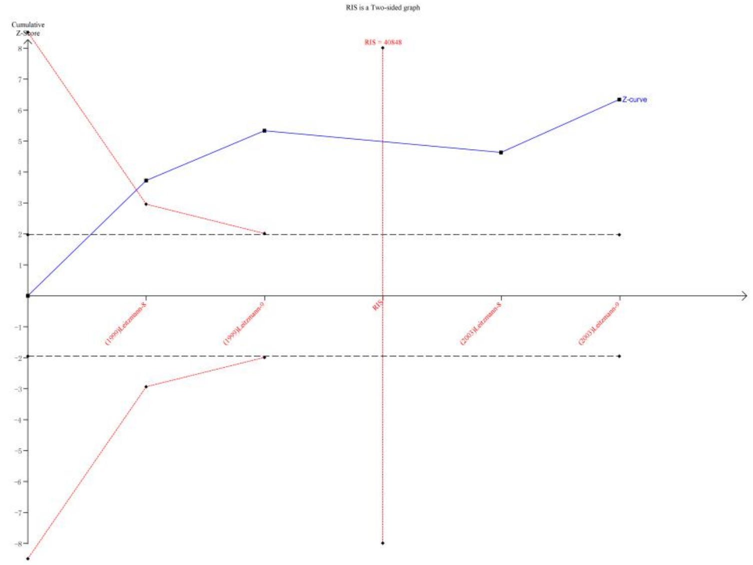

E2

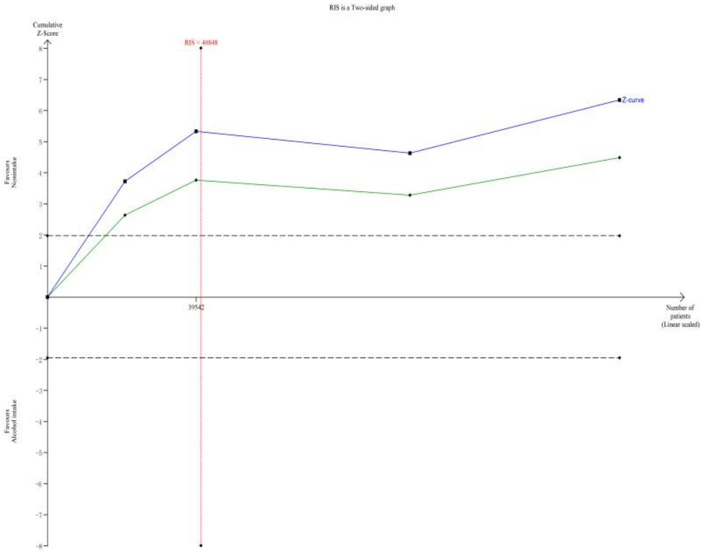

F1

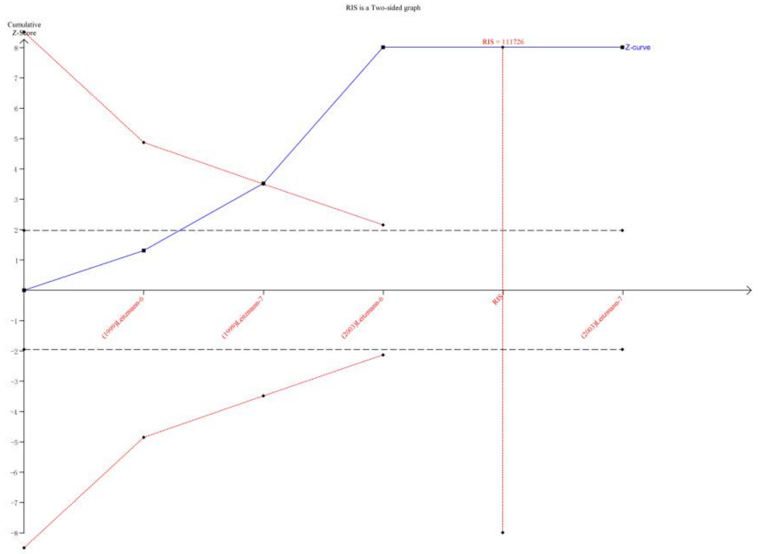

F2

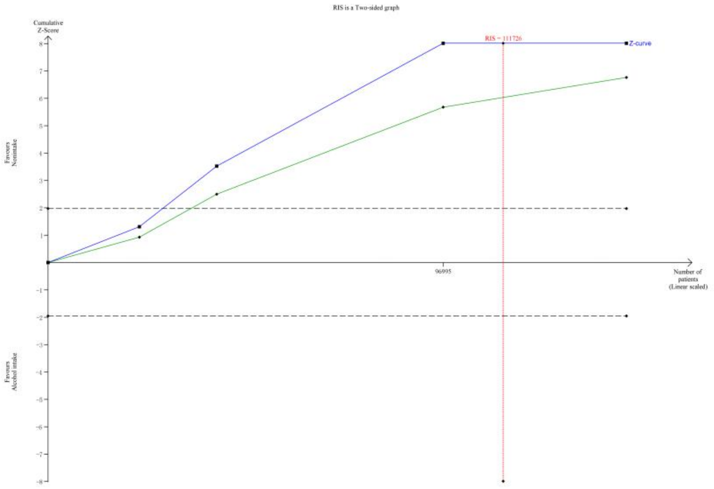

G1

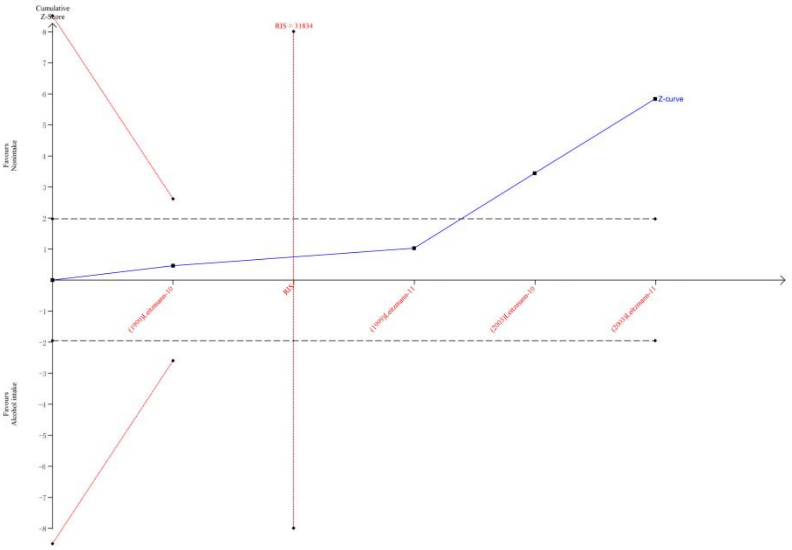

G2

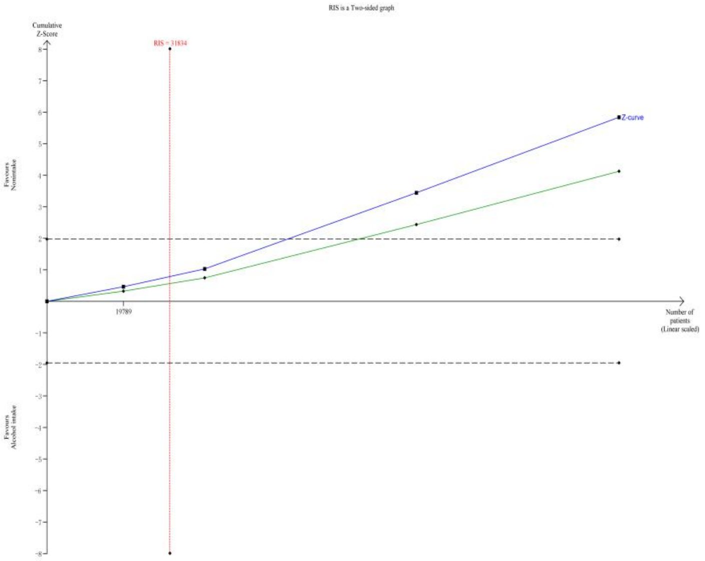

H1

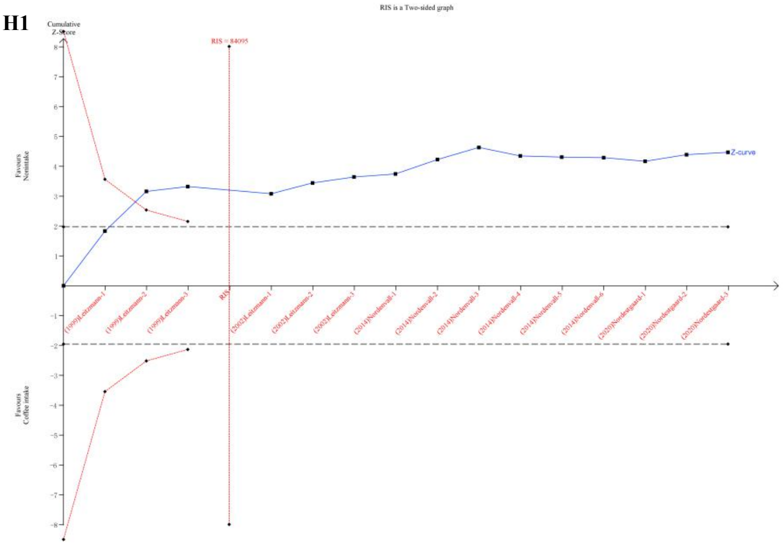

H2

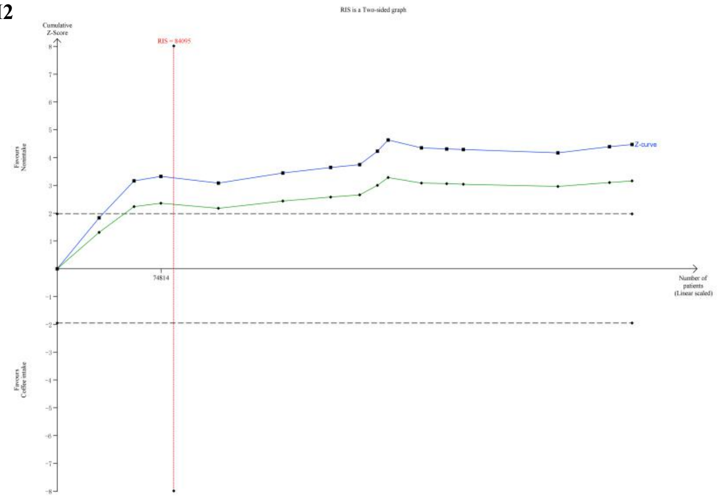

Figure 10. Trial sequential analysis in meta-analysis of addictive behaviors and the risk of gallstone disease

A. **ever smoking *vs.* never smoking** (A1: adjusted boundaries test, A2: penalized test),

B. **former smoking *vs.* never smoking** (B1: adjusted boundaries test, B2: penalized test),

C. **current smoking *vs.* never smoking** (C1: adjusted boundaries test, C2: penalized test),

D. **alcohol intake *vs.* none intake** (D1: adjusted boundaries test, D2: penalized test),

E. **beer intake *vs.* non-intake** (E1: adjusted boundaries test, E2: penalized test),

F. **wine intake *vs.* non-intake** (F1: adjusted boundaries test, F2: penalized test),

G. **liquor intake *vs.* non-intake** (G1: adjusted boundaries test, G2: penalized test),

H. **coffee consumption *vs.* non-consumption** (H1: adjusted boundaries test, H2: penalized test).

*RIS: required information size

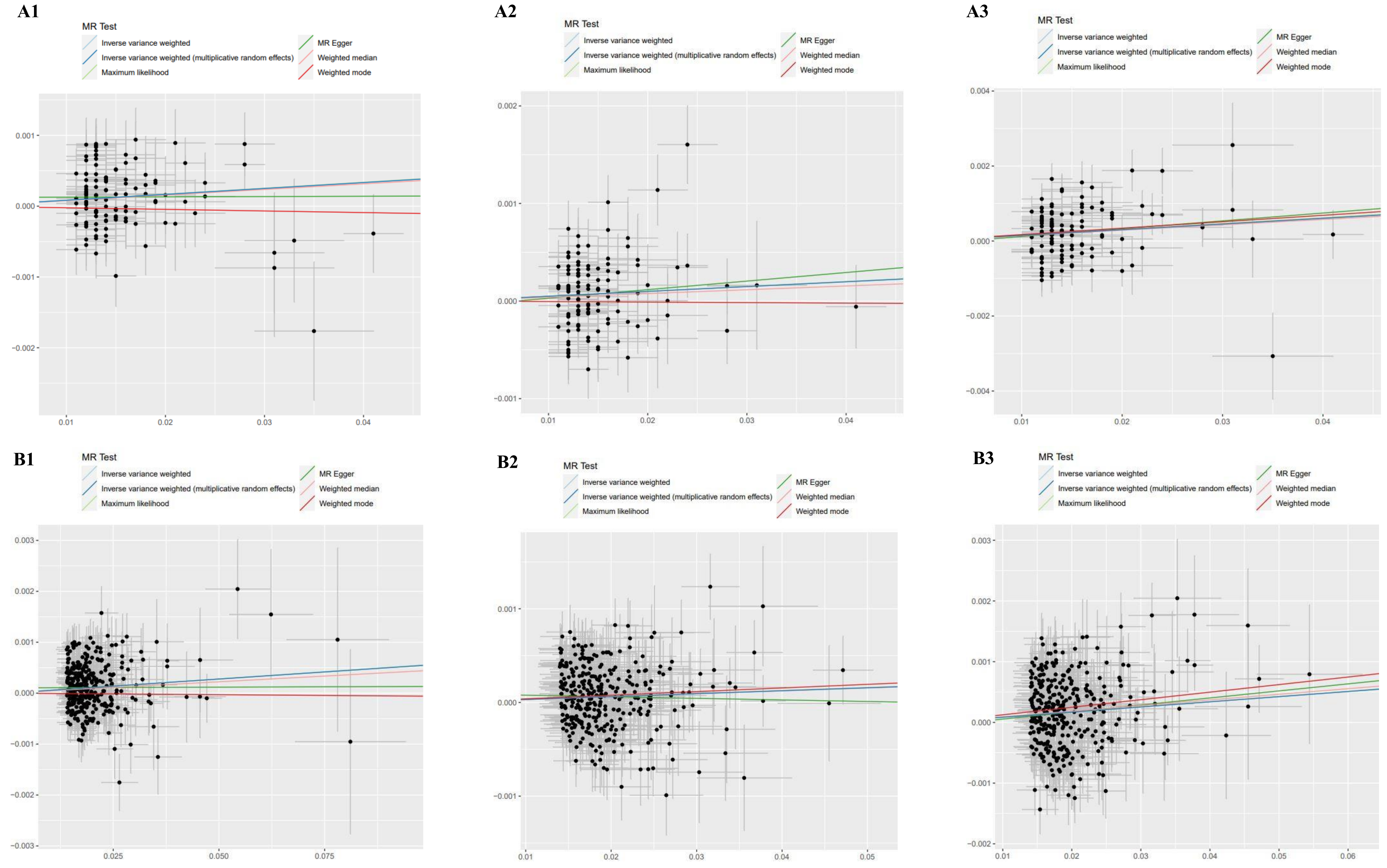
A1
MR Test
Inverse variance weighted
Inverse variance weighted (multiplicative random effects)
Maximum likelihood
MR Egger
Weighted median
Weighted mode
A2
MR Test
Inverse variance weighted
Inverse variance weighted (multiplicative random effects)
Maximum likelihood
MR Egger
Weighted median
Weighted mode
A3
MR Test
Inverse variance weighted
Inverse variance weighted (multiplicative random effects)
Maximum likelihood
MR Egger
Weighted median
Weighted mode
B1
MR Test
Inverse variance weighted
Inverse variance weighted (multiplicative random effects)
Maximum likelihood
MR Egger
Weighted median
Weighted mode
B2
MR Test
Inverse variance weighted
Inverse variance weighted (multiplicative random effects)
Maximum likelihood
MR Egger
Weighted median
Weighted mode
B3
MR Test
Inverse variance weighted
Inverse variance weighted (multiplicative random effects)
Maximum likelihood
MR Egger
Weighted median
Weighted mode

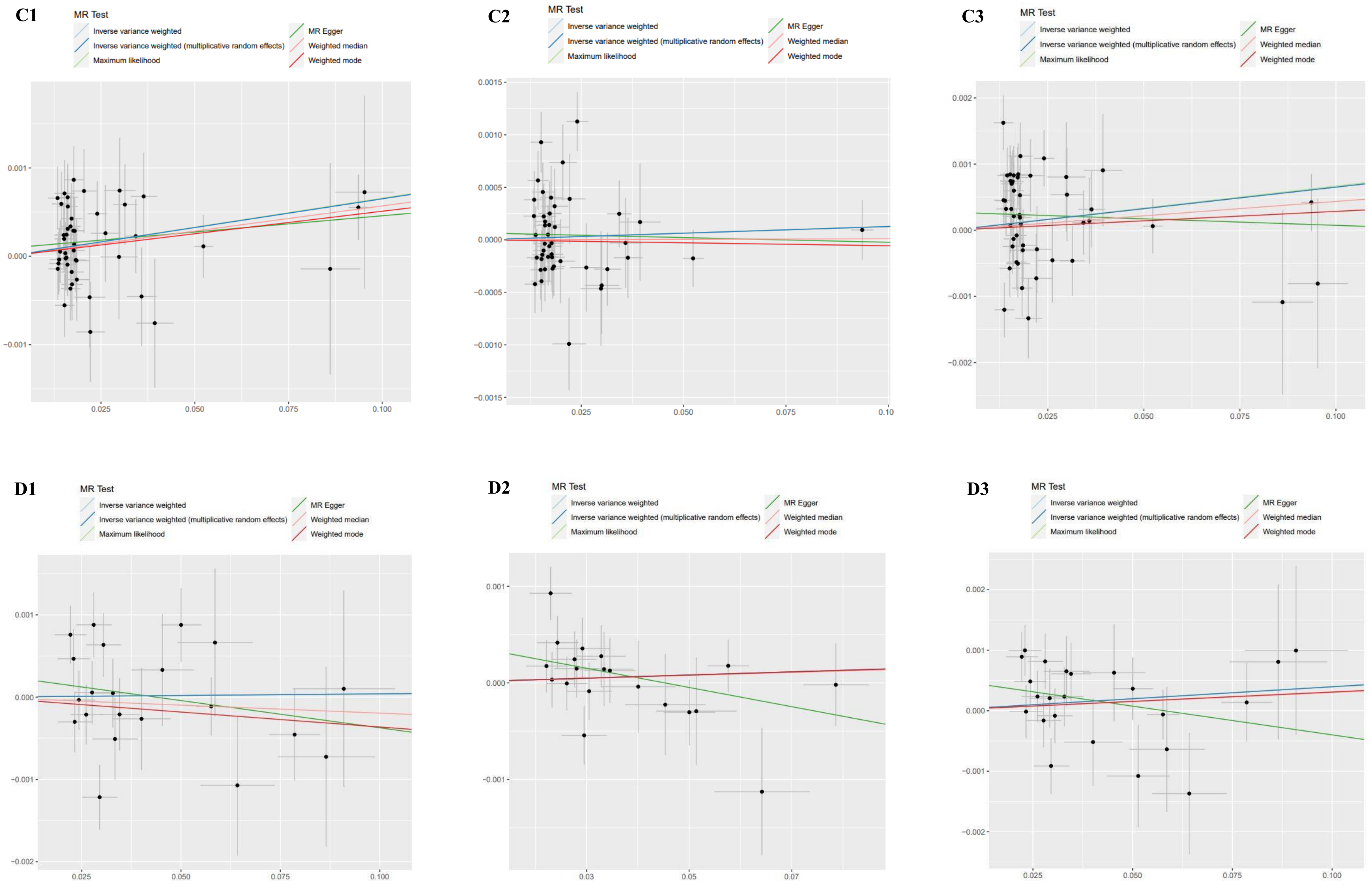
C1
MR Test
Inverse variance weighted
Inverse variance weighted (multiplicative random effects)
Maximum likelihood
MR Egger
Weighted median
Weighted mode
C2
C3
D1
D2
D3

E1

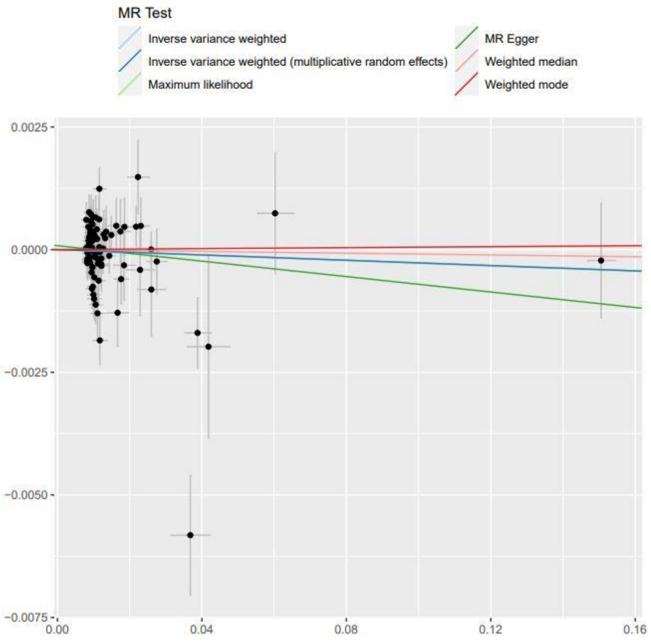

E2

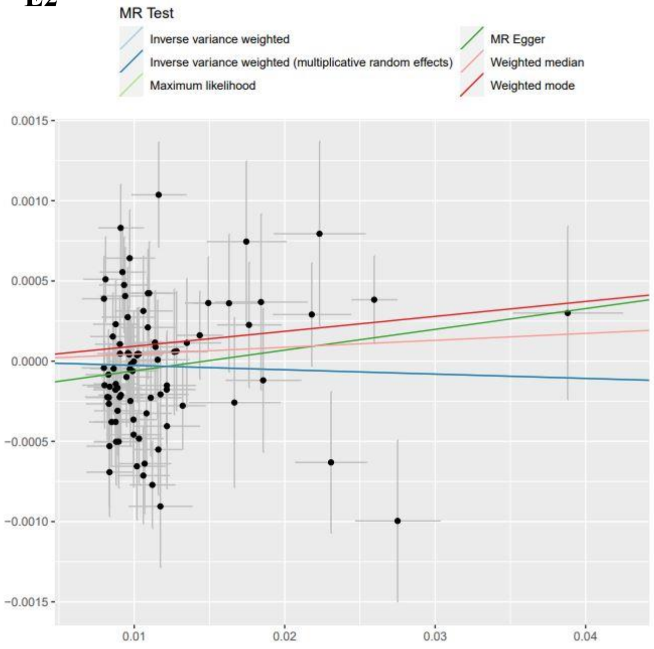

E3

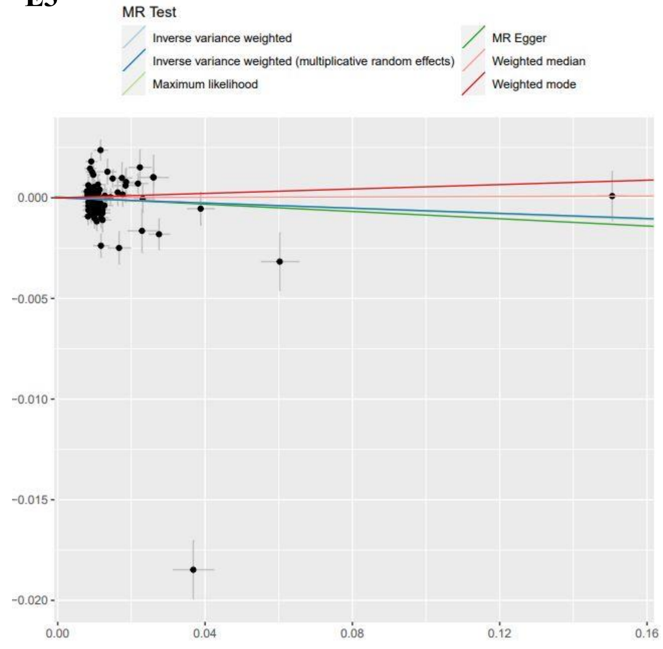

F1

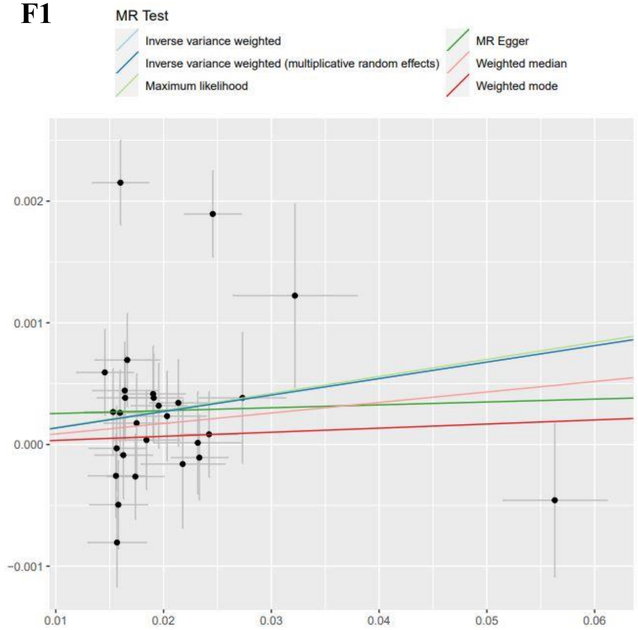

F2

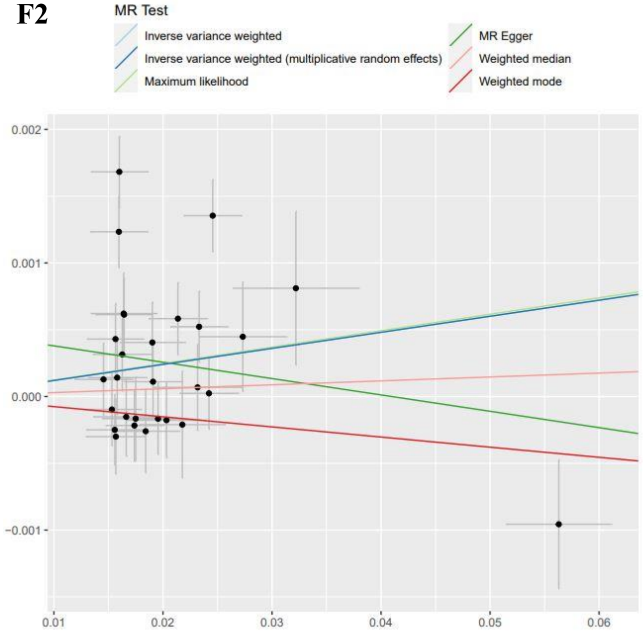

F3

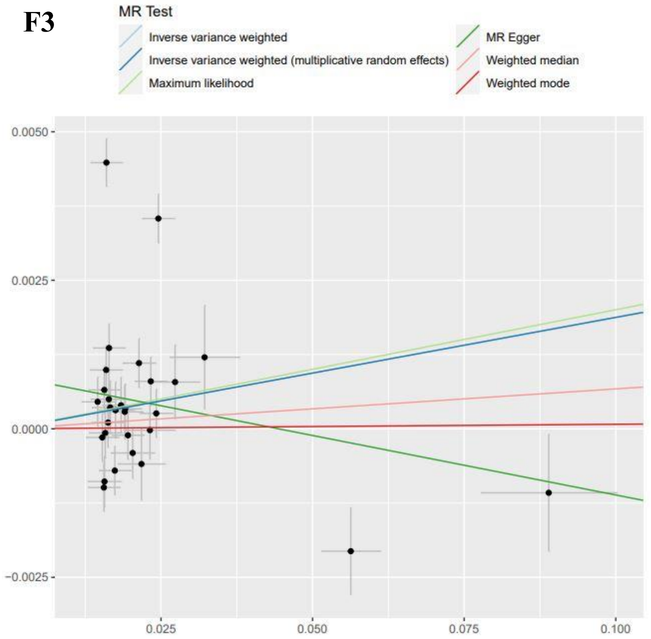

G1

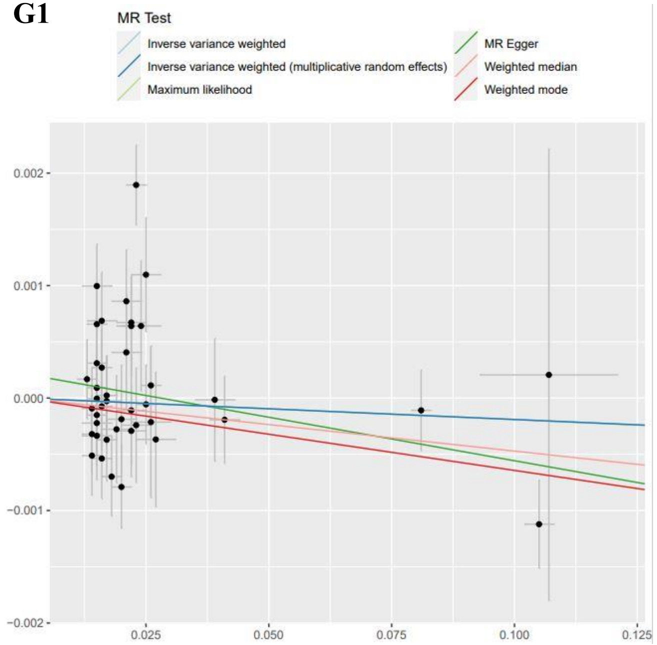

G2

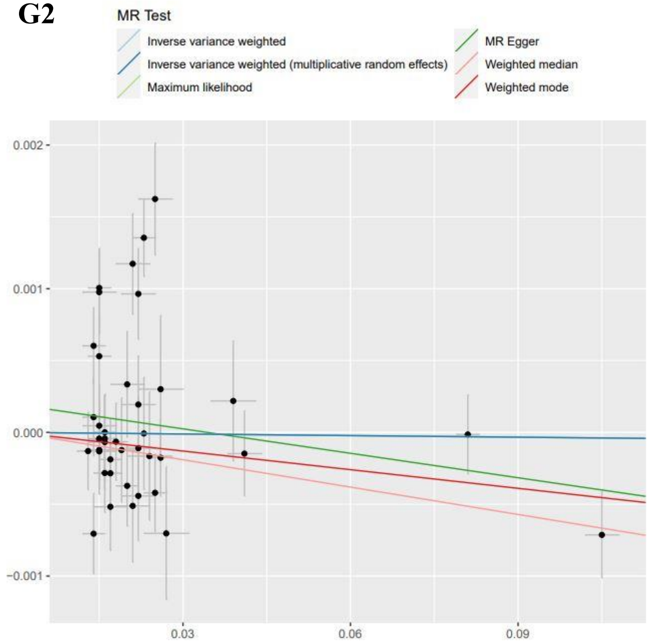

G3

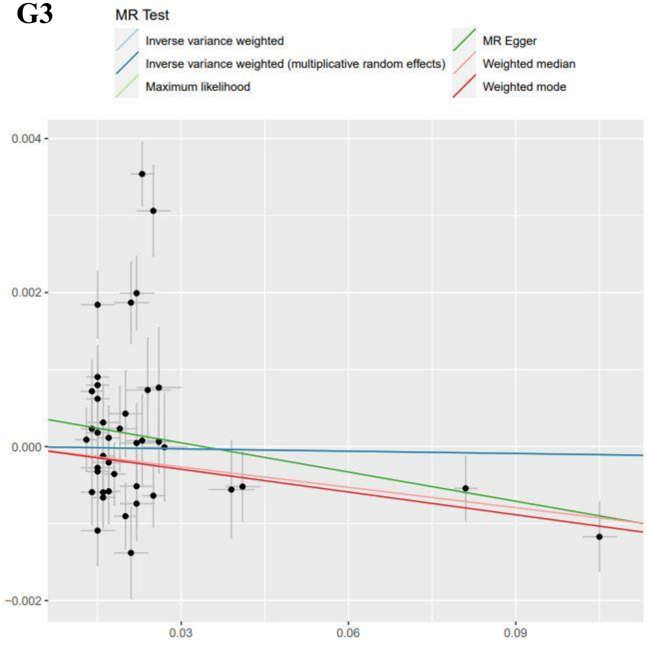

H1

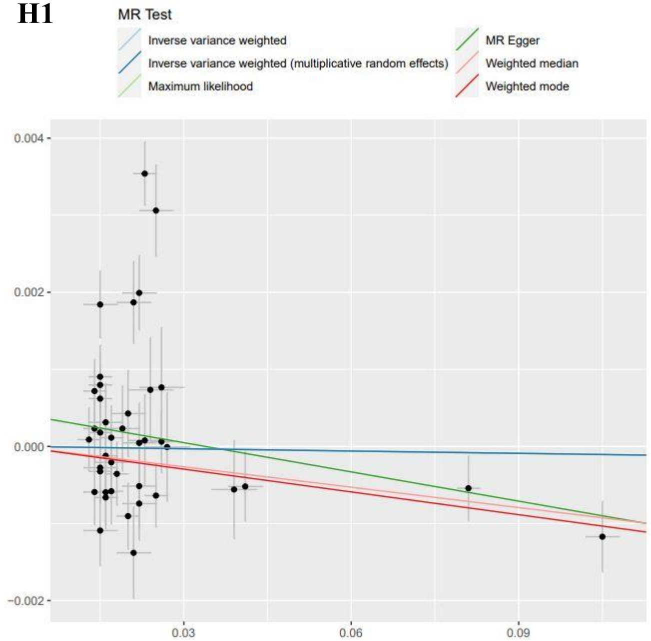

H2

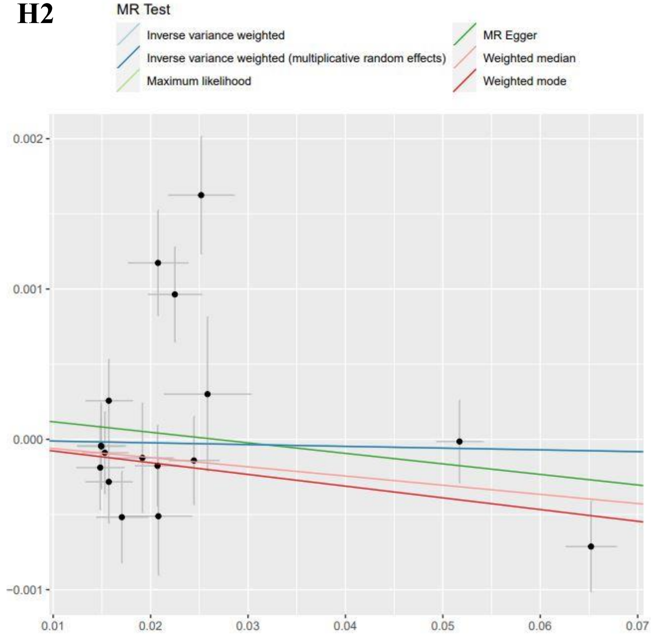

H3

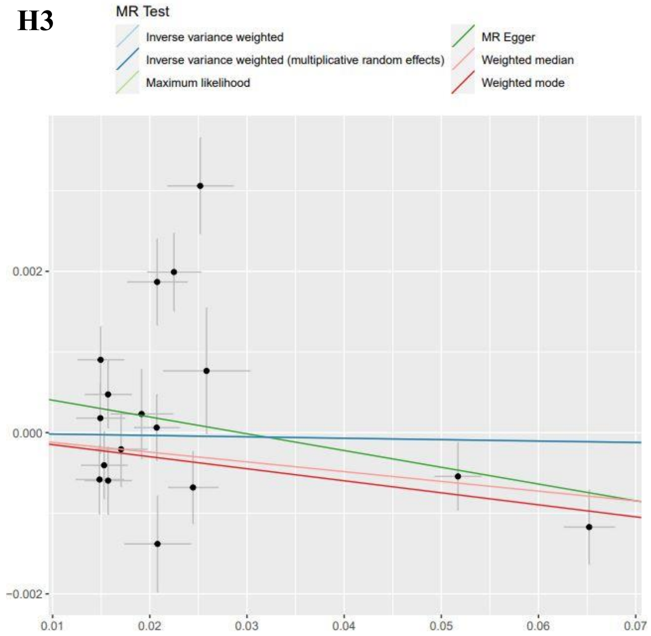

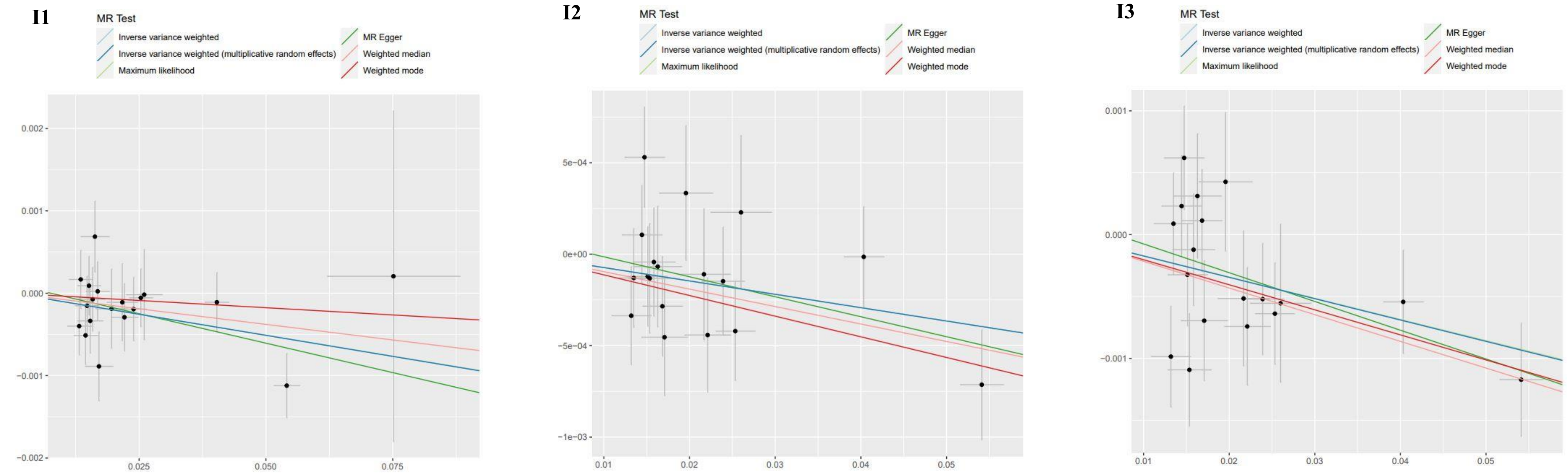

Figure 11. Scatter plots of MR-analysis in addictive behaviors and the risk of GSD

A. Lifetime smoking (A1. Diagnosed cholelithiasis, A2. Self-reported gallstones, A3. Patients underwent checystectomy);

B. Ever smoking (B1. Diagnosed cholelithiasis, B2. Self-reported gallstones, B3. Checystectomy);

C. Current smoking (C1. Diagnosed cholelithiasis, C2. Self-reported gallstones, C3. Checystectomy);

D. Smoking cessation (D1. Diagnosed cholelithiasis, D2. Self-reported gallstones, D3. Checystectomy);

E. Common alcohol use (E1. Diagnosed cholelithiasis, E2. Self-reported gallstones, E3. Checystectomy);

F. Problematic alcohol use (F1. Diagnosed cholelithiasis, F2. Self-reported gallstones, F3. Checystectomy);

G. Caffeine intake (G1. Diagnosed cholelithiasis, G2. Self-reported gallstones, G3. Checystectomy);

H. Coffee consumption (H1. Diagnosed cholelithiasis, H2. Self-reported gallstones, H3. Checystectomy);

I. Tea consumption (I1. Diagnosed cholelithiasis, I2. Self-reported gallstones, I3. Checystectomy).

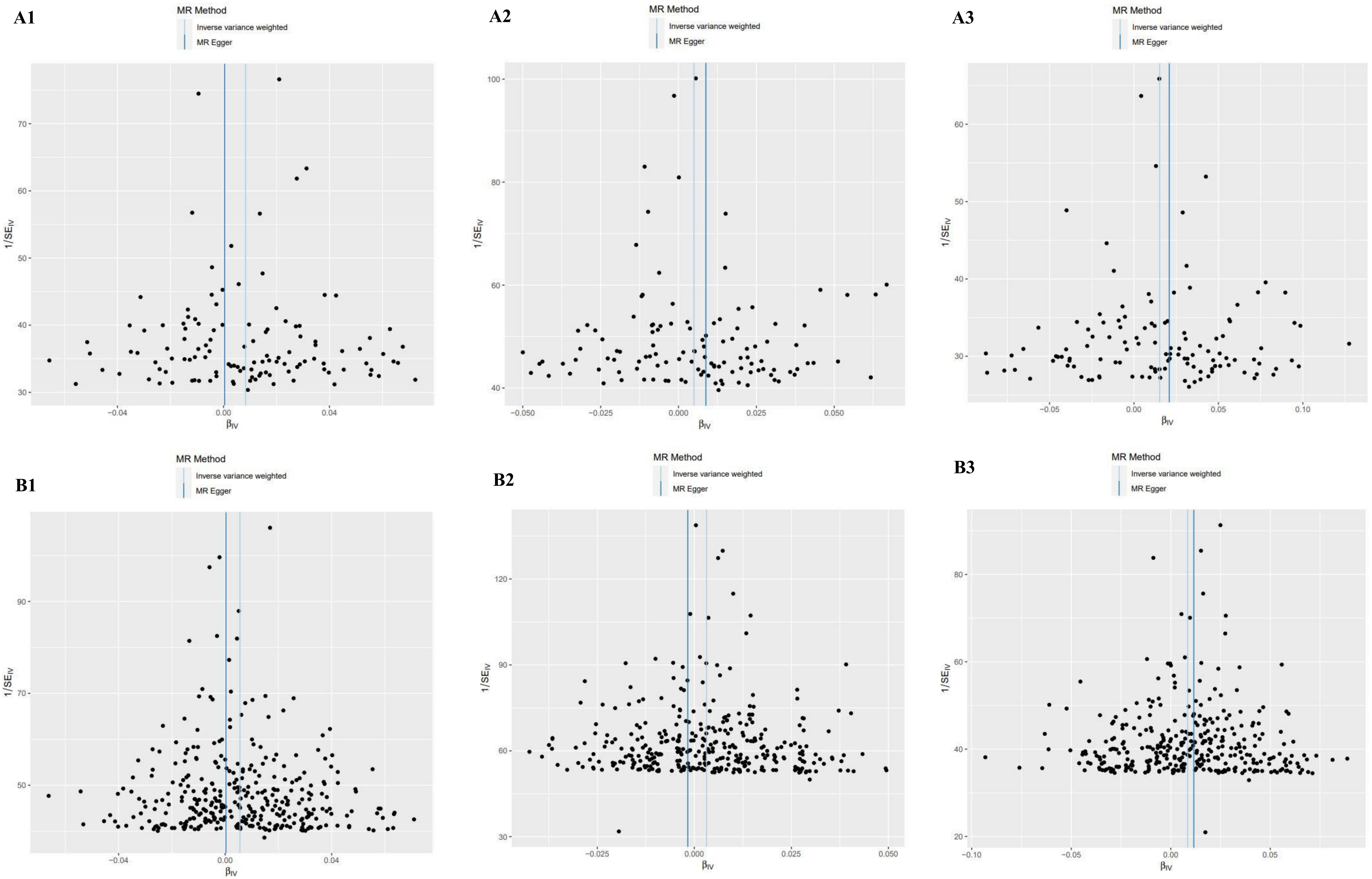
A1
A2
A3
B1
B2
B3
MR Method
Inverse variance weighted
MR Egger
1/SE_IV
β_IV

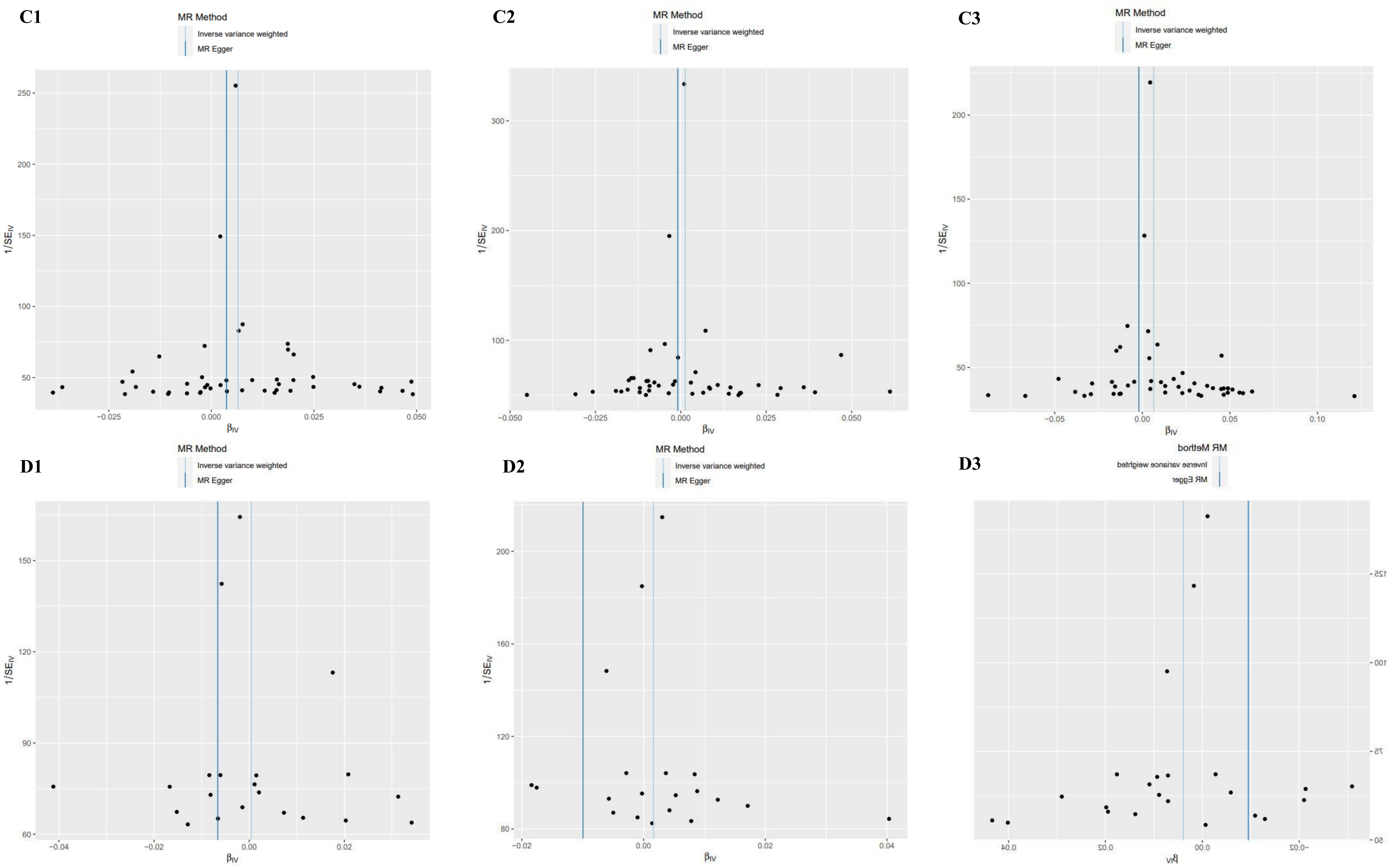
C1
C2
C3
D1
D2
D3
MR Method
Inverse variance weighted
MR Egger
$1/SE_{IV}$
β_{IV}

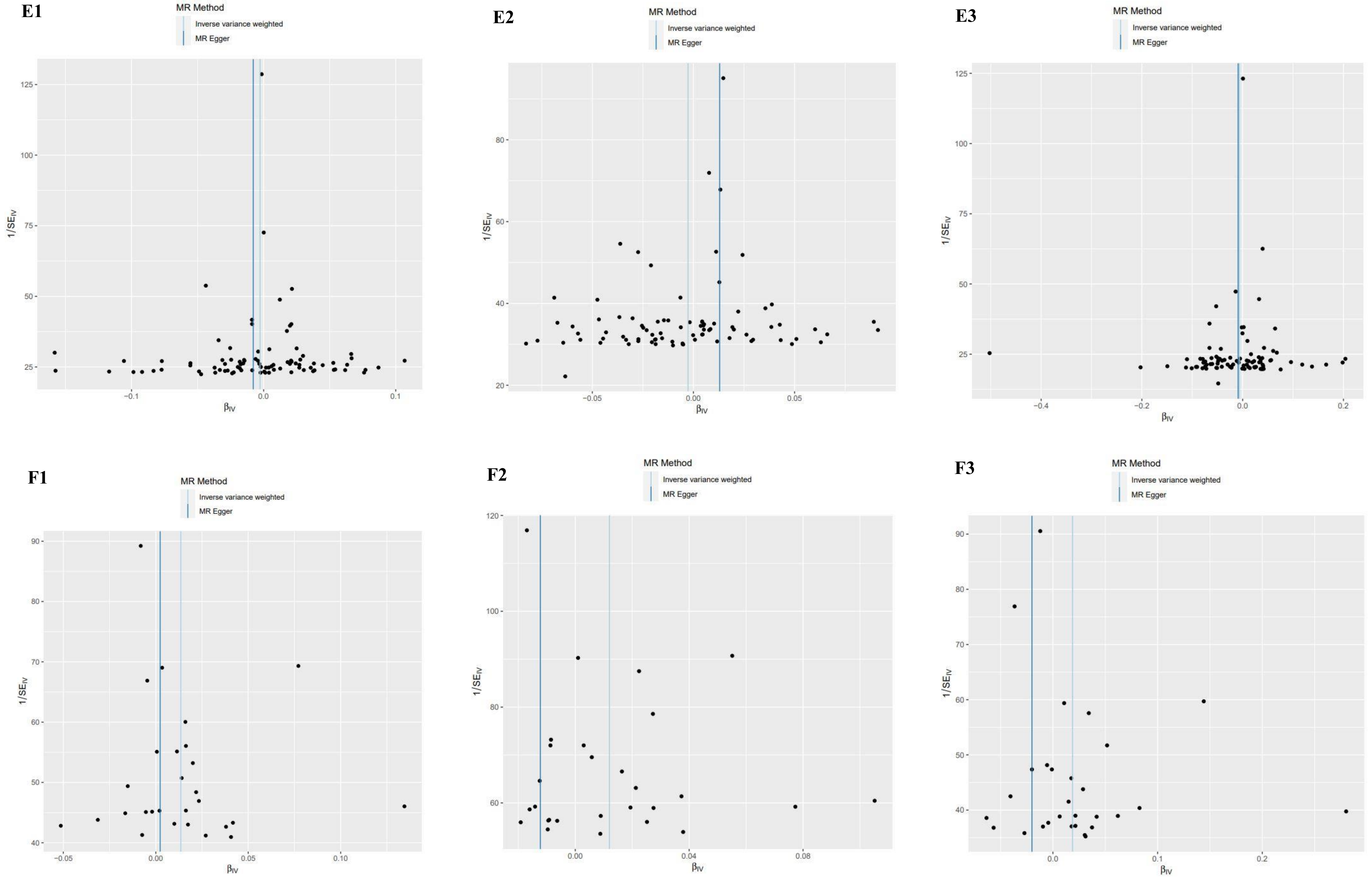
E1
E2
E3
F1
F2
F3
MR Method
Inverse variance weighted
MR Egger
1/SE$_{IV}$
β$_{IV}$

G1

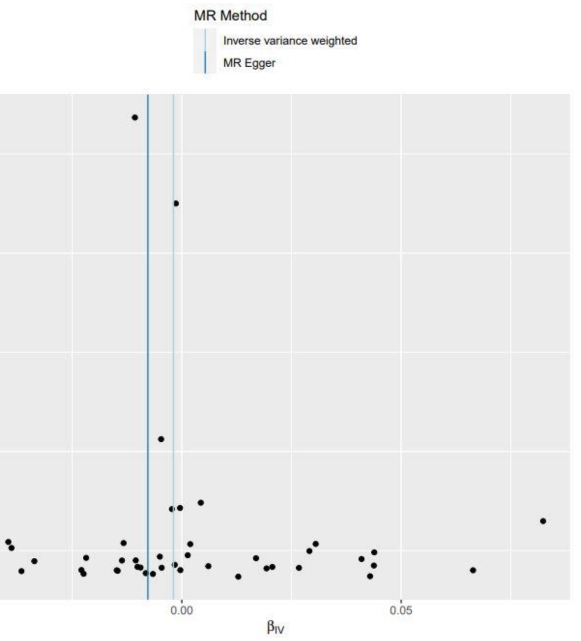

G2

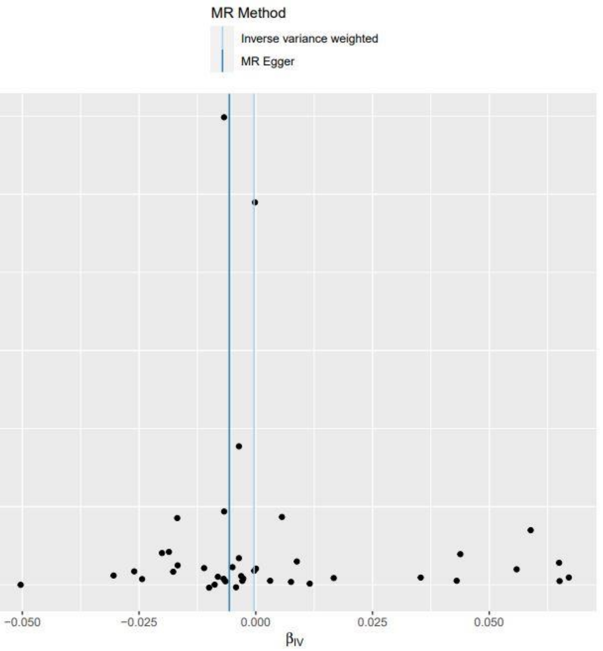

G3

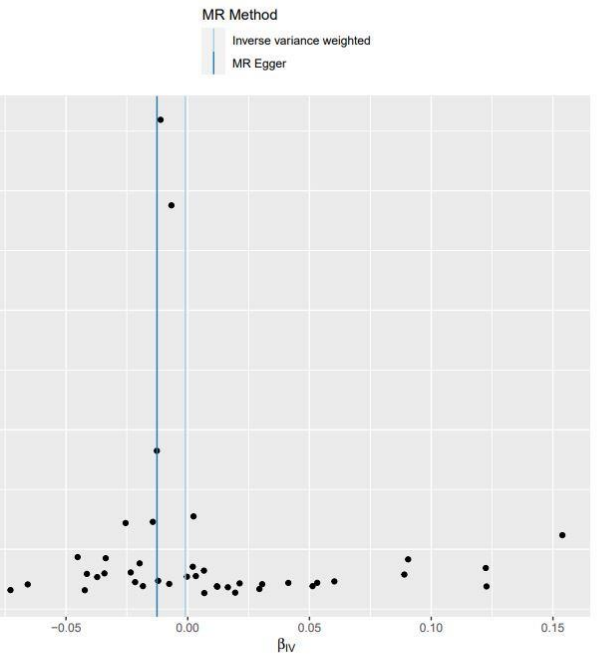

H1

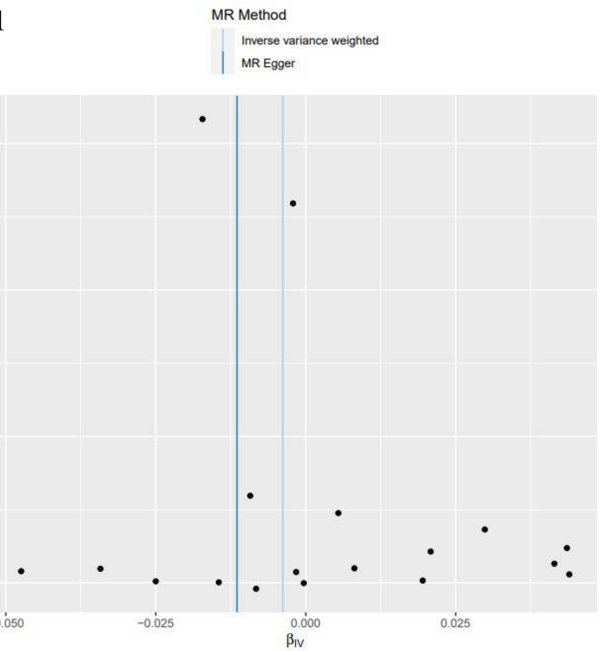

H2

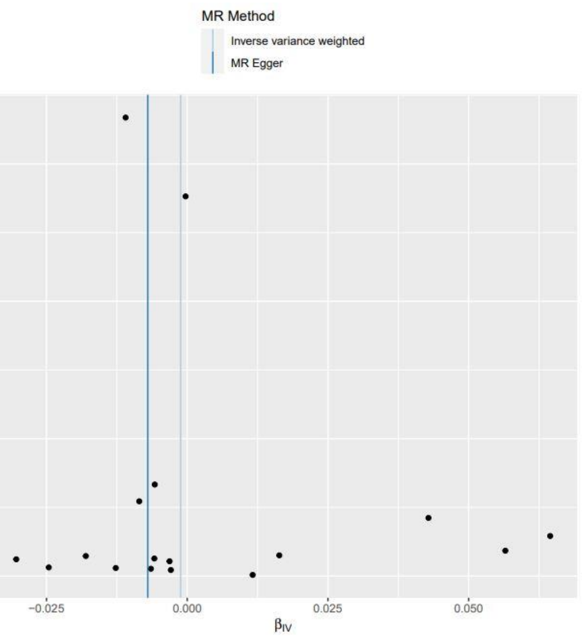

H3

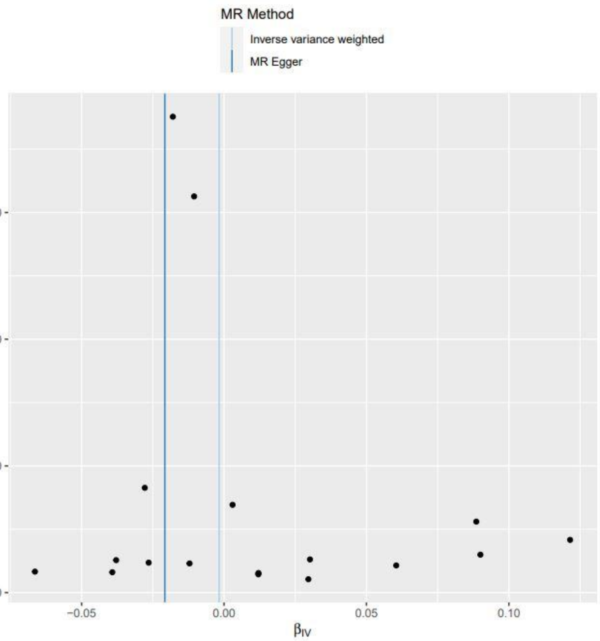

I1

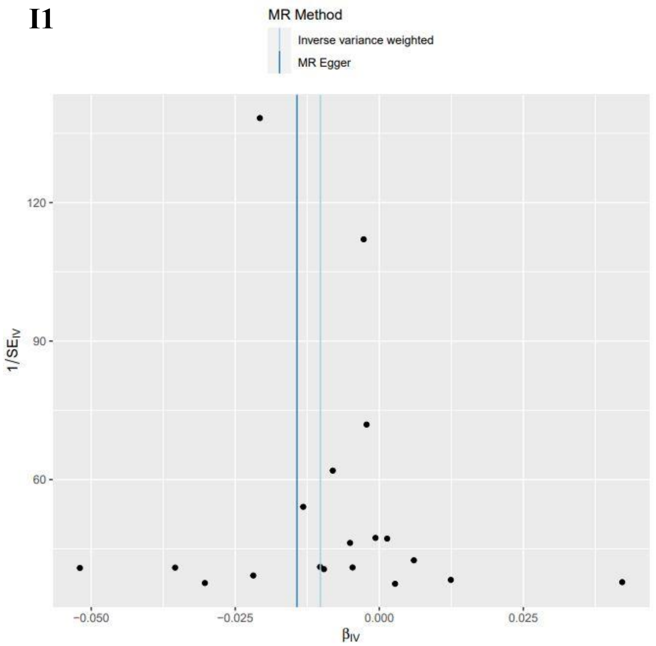

I2

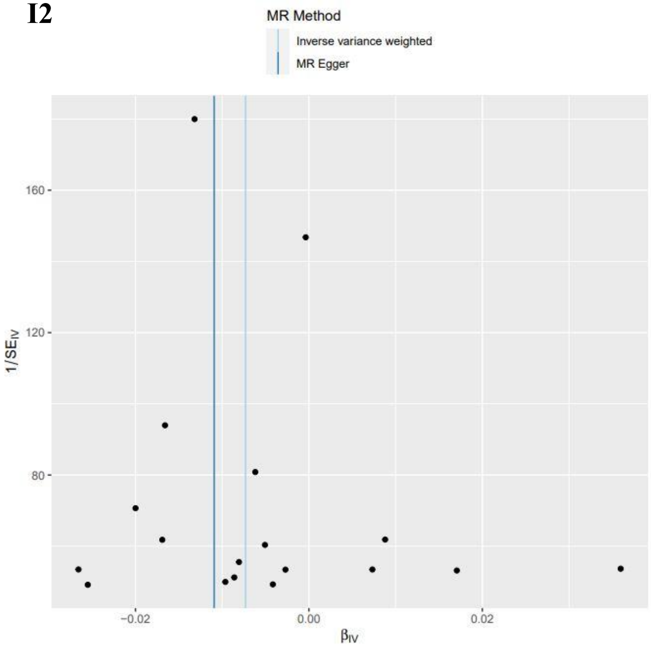

I3

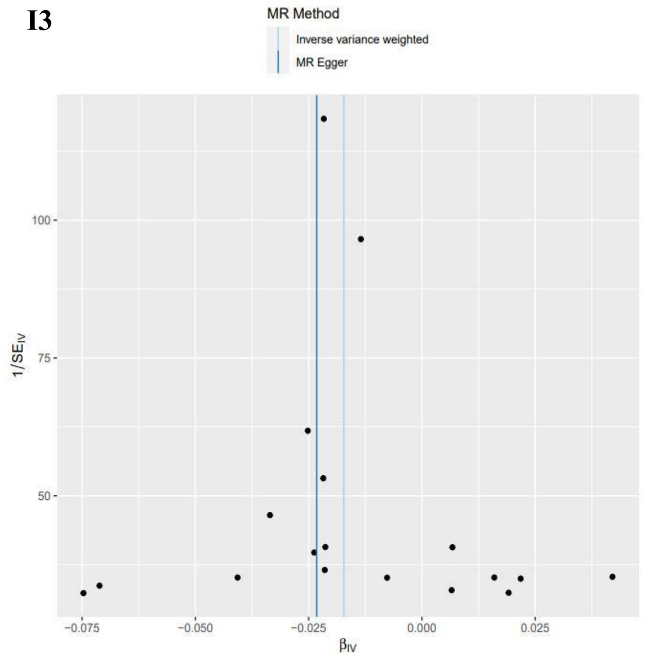

Figure 12. Funnel plots of MR-analysis in addictive behaviors and the risk of GSD

A. Lifetime smoking (A1. Diagnosed cholelithiasis, A2. Self-reported gallstones, A3. Patients underwent checystectomy);

B. Ever smoking (B1. Diagnosed cholelithiasis, B2. Self-reported gallstones, B3. Checystectomy);

C. Current smoking (C1. Diagnosed cholelithiasis, C2. Self-reported gallstones, C3. Checystectomy);

D. Smoking cessation (D1. Diagnosed cholelithiasis, D2. Self-reported gallstones, D3. Checystectomy);

E. Common alcohol use (E1. Diagnosed cholelithiasis, E2. Self-reported gallstones, E3. Checystectomy);

F. Problematic alcohol use (F1. Diagnosed cholelithiasis, F2. Self-reported gallstones, F3. Checystectomy);

G. Caffeine intake (G1. Diagnosed cholelithiasis, G2. Self-reported gallstones, G3. Checystectomy);

H. Coffee consumption (H1. Diagnosed cholelithiasis, H2. Self-reported gallstones, H3. Checystectomy);

I. Tea consumption (I1. Diagnosed cholelithiasis, I2. Self-reported gallstones, I3. Checystectomy).

PRISMA 2020 Checklist

Section and Topic	Item #	Checklist item	Location where item is reported
**TITLE**			
Title	1	Identify the report as a systematic review, meta-analysis, or both.	Title
**ABSTRACT**			
Abstract	2	See the PRISMA 2020 for Abstracts checklist.	First page
**INTRODUCTION**			
Rationale	3	Describe the rationale for the review in the context of existing knowledge.	Introduction
Objectives	4	Provide an explicit statement of the objective(s) or question(s) the review addresses.	Introduction
**METHODS**			
Eligibility criteria	5	Specify the inclusion and exclusion criteria for the review and how studies were grouped for the syntheses.	First paragraph of the methods
Information sources	6	Specify all databases, registers, websites, organisations, reference lists and other sources searched or consulted to identify studies. Specify the date when each source was last searched or consulted.	First paragraph of the method
Search strategy	7	Present the full search strategies for all databases, registers and websites, including any filters and limits used.	Supplementary Table 1
Selection process	8	Specify the methods used to decide whether a study met the inclusion criteria of the review, including how many reviewers screened each record and each report retrieved, whether they worked independently, and if applicable, details of automation tools used in the process.	Second paragraph of the methods
Data collection process	9	Specify the methods used to collect data from reports, including how many reviewers collected data from each report, whether they worked independently, any processes for obtaining or confirming data from study investigators, and if applicable, details of automation tools used in the process.	Second paragraph of the methods
Data items	10a	List and define all outcomes for which data were sought. Specify whether all results that were compatible with each outcome domain in each study were sought (e.g. for all measures, time points, analyses), and if not, the methods used to decide which results to collect.	Second paragraph of the methods
	10b	List and define all other variables for which data were sought (e.g. participant and intervention characteristics, funding sources). Describe any assumptions made about any missing or unclear information.	Third paragraph of the methods
Study risk of bias assessment	11	Specify the methods used to assess risk of bias in the included studies, including details of the tool(s) used, how many reviewers assessed each study and whether they worked independently, and if applicable, details of automation tools used in the process.	Third paragraph of the methods
Effect measures	12	Specify for each outcome the effect measure(s) (e.g. risk ratio, mean difference) used in the synthesis or presentation of results.	Second & third paragraphs of the methods
Synthesis methods	13a	Describe the processes used to decide which studies were eligible for each synthesis (e.g. tabulating the study intervention characteristics and comparing against the planned groups for each synthesis (item #5)).	Third paragraph of the methods & Supplementary Figure 1
	13b	Describe any methods required to prepare the data for presentation or synthesis, such as handling of missing summary statistics, or data conversions.	
	13c	Describe any methods used to tabulate or visually display results of individual studies and syntheses.	
	13d	Describe any methods used to synthesize results and provide a rationale for the choice(s). If meta-analysis was performed, describe the model(s), method(s) to identify the presence and extent of statistical heterogeneity, and software package(s) used.	
	13e	Describe any methods used to explore possible causes of heterogeneity among study results (e.g. subgroup analysis, meta-regression).	
	13f	Describe any sensitivity analyses conducted to assess robustness of the synthesized results.	
Reporting bias assessment	14	Describe any methods used to assess risk of bias due to missing results in a synthesis (arising from reporting biases).	Third paragraph of the methods

PRISMA 2020 Checklist

Section and Topic	Item #	Checklist item	Location where item is reported
Certainty assessment	15	Describe any methods used to assess certainty (or confidence) in the body of evidence for an outcome.	Third paragraph of the methods
**RESULTS**			
Study selection	16a	Describe the results of the search and selection process, from the number of records identified in the search to the number of studies included in the review, ideally using a flow diagram.	Supplementary Figure 1
	16b	Cite studies that might appear to meet the inclusion criteria, but which were excluded, and explain why they were excluded.	
Study characteristics	17	Cite each included study and present its characteristics.	Supplementary Table 2
Risk of bias in studies	18	Present assessments of risk of bias for each included study.	Supplementary Figure 9
Results of individual studies	19	For all outcomes, present, for each study: (a) summary statistics for each group (where appropriate) and (b) an effect estimate and its precision (e.g. confidence/credible interval), ideally using structured tables or plots.	Supplementary Figure 2 & Table 1
Results of syntheses	20a	For each synthesis, briefly summarise the characteristics and risk of bias among contributing studies.	Supplementary Figure 2
	20b	Present results of all statistical syntheses conducted. If meta-analysis was done, present for each the summary estimate and its precision (e.g. confidence/credible interval) and measures of statistical heterogeneity. If comparing groups, describe the direction of the effect.	Table 1
	20c	Present results of all investigations of possible causes of heterogeneity among study results.	Supplementary Figure 8
	20d	Present results of all sensitivity analyses conducted to assess the robustness of the synthesized results.	Supplementary Figures 3-9
Reporting biases	21	Present assessments of risk of bias due to missing results (arising from reporting biases) for each synthesis assessed.	Supplementary Figure 9
Certainty of evidence	22	Present assessments of certainty (or confidence) in the body of evidence for each outcome assessed.	Table 1 & Supplementary Figure 2
**DISCUSSION**			
Discussion	23a	Provide a general interpretation of the results in the context of other evidence.	First paragraph of the discussion
	23b	Discuss any limitations of the evidence included in the review.	Discussion part
	23c	Discuss any limitations of the review processes used.	Fifth paragraph of the discussion
	23d	Discuss implications of the results for practice, policy, and future research.	Last one paragraph of the discussion
**OTHER INFORMATION**			
Registration and protocol	24a	Provide registration information for the review, including register name and registration number, or state that the review was not registered.	CRD42020179076
	24b	Indicate where the review protocol can be accessed, or state that a protocol was not prepared.	First paragraph of the methods
	24c	Describe and explain any amendments to information provided at registration or in the protocol.	None

PRISMA 2020 Checklist

Section and Topic	Item #	Checklist item	Location where item is reported
Support	25	Describe sources of financial or non-financial support for the review, and the role of the funders or sponsors in the review.	Funding
Competing interests	26	Declare any competing interests of review authors.	Declaration of interests
Availability of data, code and other materials	27	Report which of the following are publicly available and where they can be found: template data collection forms; data extracted from included studies; data used for all analyses; analytic code; any other materials used in the review.	None

From: Page MJ, McKenzie JE, Bossuyt PM, Boutron I, Hoffmann TC, Mulrow CD, et al. The PRISMA 2020 statement: an updated guideline for reporting systematic reviews. BMJ 2021;372:n71. doi: 10.1136/bmj.n71

For more information, visit: http://www.prisma-statement.org/
